# Comparative effectiveness of exercise modalities and nutritional supplementation for sarcopenic obesity in older adults: a network meta-analysis based on randomized controlled trials

**DOI:** 10.3389/fpubh.2026.1775783

**Published:** 2026-02-23

**Authors:** Jiacheng Yu, Xinchun Li, Hao Yu, Yijun Huang

**Affiliations:** School of Physical Education, Ludong University, Yantai, China

**Keywords:** multicomponent training, network meta-analysis, nutritional supplementation, older adults, resistance training, sarcopenic obesity

## Abstract

**Introduction:**

Sarcopenic obesity is highly prevalent among older adults and is associated with adverse clinical outcomes. However, direct comparative evidence on the relative efficacy and safety of different exercise-based rehabilitation strategies, with or without nutritional supplementation or high-protein intake, remains limited. This study aimed to compare and rank the effects of diverse rehabilitation interventions using a systematic review and network meta-analysis.

**Methods:**

PubMed, Embase, the Cochrane Library, and Web of Science were systematically searched from database inception to November 1, 2025, without language restrictions. Both Medical Subject Headings and free-text terms were used. Primary outcomes included body mass index (BMI), handgrip strength (GRIP), fat mass (FM), percentage body fat (PBF), and skeletal muscle index (SMI). A systematic review and network meta-analysis were conducted. Risk of bias was assessed using the Cochrane Risk of Bias 2 (ROB 2) tool, and the certainty of evidence was evaluated using the CINeMA framework. The study protocol was prospectively registered in PROSPERO (CRD420251270452).

**Results:**

Twenty-four randomized controlled trials involving 1,298 participants and nine distinct exercise- and nutrition-related rehabilitation strategies were included. For BMI, only multicomponent training (MC) significantly reduced BMI compared with usual care (UC) (MD = −1.08, 95% CI −1.86 to −0.29) and ranked highest (SUCRA 85.1%). For handgrip strength, both resistance training (RT) (MD = 3.96, 95% CI 2.15–5.77) and MC (MD = 2.13, 95% CI 0.25–4.01) were superior to UC, with RT ranking first (SUCRA 90.9%). For fat mass, only RT significantly reduced FM compared with UC (MD = −2.30, 95% CI −3.63 to −0.98) and achieved the highest ranking (SUCRA 79.0%). For PBF, both MC (MD = −3.53, 95% CI −5.70 to −1.36) and RT (MD = −2.30, 95% CI −3.98 to −0.62) were effective, with MC ranking highest (SUCRA 77.1%). No intervention demonstrated a statistically significant advantage over UC for SMI; however, MC combined with nutritional supplementation ranked relatively favorably (SUCRA 74.1%). Global consistency testing supported overall network coherence. Sensitivity analyses confirmed the robustness of the findings, and comparison-adjusted funnel plots indicated no clear evidence of publication bias.

**Conclusion:**

In older adults with sarcopenic obesity, exercise-centered interventions yield clinically meaningful benefits across several key rehabilitation outcomes. Overall, resistance training appears particularly effective for improving muscle strength and reducing adiposity-related measures, whereas multicomponent training shows greater advantages in reducing BMI and PBF. Evidence for improvements in SMI remains limited and uncertain, highlighting the need for larger, well-designed randomized trials with longer follow-up and direct head-to-head comparisons to clarify long-term benefits and identify optimal intervention combinations.

**Systematic review registration:**

https://www.crd.york.ac.uk/PROSPERO/view/CRD420251270452, PROSPERO: CRD420251270452.

## 1 Introduction

Sarcopenic obesity is an increasingly recognized composite phenotype in older adults, defined by the coexistence of sarcopenia and excess adiposity and associated with additive adverse health risks (1). Sarcopenia is commonly linked to insufficient physical activity, chronic low-grade inflammation, inadequate nutritional intake, and anabolic resistance. The concomitant presence of obesity further aggravates insulin resistance and systemic inflammation, while promoting abnormal fat infiltration within and around skeletal muscle, thereby accelerating declines in muscle strength and functional capacity (2). Among individuals aged 65 years and older, sarcopenia is strongly associated with disability, reduced quality of life, and premature mortality; when accompanied by obesity, the risks of adverse cardiovascular outcomes and metabolic complications increase substantially (2, 3). With advancing age, fat mass typically increases, whereas muscle mass and strength progressively decline, creating a biological environment conducive to the development of sarcopenic obesity. Prevalence estimates vary widely across studies, reflecting marked heterogeneity in diagnostic criteria, definitions of obesity, assessment methods, and the ethnic and comorbidity profiles of the populations examined (4–6). A growing body of research suggests that when sarcopenic obesity and knee osteoarthritis team up, they can lead to metabolic syndrome, stiffer arteries, and trouble with everyday tasks, potentially even bumping up the odds of mortality from any cause. Consequently, the early identification and targeted management of sarcopenic obesity are of considerable clinical and public health importance (7–11).

At present, no specific or consistently effective pharmacological therapy exists for sarcopenic obesity, and non-pharmacological strategies remain the foundation of management. Unlike isolated obesity or sarcopenia, therapeutic goals in sarcopenic obesity prioritize fat mass reduction while preserving skeletal muscle, with the dual objective of improving body composition and maintaining or enhancing muscle strength and physical function. Consequently, integrated interventions combining exercise and nutritional strategies are generally required. From an exercise perspective, RT improves muscle mass and strength through mechanisms that include satellite cell activation, stimulation of muscle protein synthesis, and attenuation of proteolysis, and may also exert favorable effects on resting energy expenditure and overall body composition (12). In contrast, aerobic training (AT) primarily enhances cardiorespiratory fitness and lipid metabolism (13). MC typically integrates resistance, balance, flexibility, and aerobic elements and is therefore more closely aligned with the functional demands of older adults. From a dietary perspective, getting enough protein—especially supplements packed with leucine-rich essential amino acids—gives your body the building blocks it needs to build muscle tissue and might help counteract the natural tendency for protein synthesis to slow down with age. Meanwhile, taking vitamin D could be your ticket to preventing tumbles and broken bones, thanks to its positive effects on how your muscles work and your bones process nutrients. In addition, certain dietary supplements, such as soy isoflavone–containing preparations, have been suggested to exert beneficial effects on lipid metabolism, vascular function, and glycemic regulation (14–16). Importantly, sarcopenic obesity in older adults is frequently accompanied by multiple comorbidities and polypharmacy. Excessive caloric restriction may accelerate the loss of lean mass, high-protein strategies require careful consideration of renal function and overall dietary balance, and exercise prescriptions should be individualized to minimize the risks of musculoskeletal injury and falls. Achieving an appropriate balance between efficacy and safety is therefore central to the selection and implementation of intervention strategies.

Although randomized controlled trials and several meta-analyses suggest that RT and MC may be effective in improving body composition and functional outcomes, direct head-to-head comparisons remain limited. Most available trials have evaluated these interventions only against usual care or general health advice, resulting in a paucity of robust comparative evidence (17, 18). In this context, a network meta-analysis is particularly warranted, as it allows the integration of both direct and indirect evidence to facilitate a comparative evaluation and ranking of different exercise modalities and nutritional strategies. Where assumptions of comparability are met, this approach enables a comprehensive assessment of their relative effects on BMI, adiposity-related outcomes (FM and PBF), and indices of muscle strength and muscle mass (GRIP and SMI). Such evidence synthesis is intended to provide a more clinically actionable and evidence-based framework to support rehabilitation decision-making and intervention selection in older adults with sarcopenic obesity.

## 2 Materials and methods

This study was conducted as a network meta-analysis (NMA) and reported in accordance with the PRISMA extension for network meta-analyses (PRISMA-NMA) (Supplementary Table S1). To enhance methodological transparency and reproducibility, the study protocol was prospectively registered in the PROSPERO database (CRD420251270452).

### 2.1 Data sources and search strategy

A comprehensive literature search was performed in PubMed, Embase, the Cochrane Library, and Web of Science. The search strategy combined controlled vocabulary terms with free-text keywords and covered the period from database inception to November 1, 2025, with no language restrictions applied. Search terms included *“sarcopenia,” “sarcopenias,” “obesity,” “aged,” “older adults,” “sports,” “athletics,” “resistance training,” “physical exercise,” “proteins,” “protein gene products,”* and *“randomized controlled trial”* (Supplementary Table S2).

### 2.2 Selection criteria

Inclusion Criteria:

1. Randomized controlled trials (RCTs) enrolling older adults with sarcopenic obesity were eligible. Sarcopenia was defined as appendicular skeletal muscle mass (kg) divided by body weight (kg) × 100%, with cut-off values of ≤32.5% for men and ≤25.7% for women. Obesity was defined as a BMI ≥ 25 kg/m^2^ (19).
2. Eligible exercise interventions included aerobic training, resistance training, combined aerobic and resistance training, and multicomponent training, defined as integrated programs comprising two or more exercise modalities (most commonly resistance, aerobic, balance, and flexibility training). Nutritional interventions included high-protein diets and nutritional supplementation, such as isoflavones and vitamin D. Control conditions consisted of usual care, health education, habitual diet, or other non-specific interventions, as defined by the original investigators.
3. Research needed to document a minimum of the following results: BMI, calculated using the standard formula (kg/m^2^); FM, defined as the total mass of adipose tissue, including subcutaneous, visceral, and essential fat; GRIP, measured using an electronic or spring-type dynamometer, with assessment procedures conducted in accordance with the AWGS 2019 recommendations (20). PBF, expressed as body fat percentage (BF%) (21). SMI, calculated as muscle mass relative to height (e.g., appendicular skeletal muscle mass/height^2^, kg/m^2^), as defined in the original study.

Exclusion criteria:

1. When the same study population was reported in multiple publications or across different follow-up periods, only the most recent and most comprehensive report was included; all other versions were excluded.
2. Research papers that failed to present quantifiable results—such as averages, measures of dispersion, variance metrics, or other transformable statistical information—were systematically omitted from this analysis, particularly when attempts to acquire the necessary figures directly from the original investigators proved fruitless.
3. Scholarly works such as case–control studies, cohort analyses, case series, and individual case reports were set aside, alongside reviews and conference presentations that failed to provide comprehensive data.

### 2.3 Data extraction

Two investigators independently extracted data from eligible randomized controlled trials in accordance with the Preferred Reporting Items for Systematic Reviews and Meta-Analyses (PRISMA) framework, with all entries cross-checked for accuracy. Any discrepancies were resolved through discussion with a third author until consensus was reached. From each study, the following information was extracted: first author, year of publication, mean participant age, geographic location, follow-up duration, intervention protocols for the experimental and control groups, and the corresponding outcome measures. For continuous outcomes (BMI, GRIP, FM, PBF, and SMI), priority was given to extracting the mean change from baseline and its standard deviation (SD). When only baseline and post-intervention values were reported, change scores and their SDs were calculated using standardized formulas. If the within-participant correlation coefficient (r) was not reported and could not be derived, an assumed value of *r* = 0.5 was applied. The robustness of this assumption was subsequently evaluated through sensitivity analyses.


$$\begin{array}{l} \text{MEANchange}=\text{Endpoint}\;\text{Mean}-\text{Baseline Mean}\\ \begin{array}{l} \text{SDchange}=\\ \sqrt{\text{Baseline}\;{\mathrm{SD}}^{2}+\text{Endpoint}\;\;{\mathrm{SD}}^{2}-2R\cdot \text{Baseline}\;\mathrm{SD}\cdot \text{Endpoint}\;\mathrm{SD}} \end{array} \end{array}$$

### 2.4 Quality assessment

The methodological quality of the included randomized controlled trials was assessed using the Cochrane Risk of Bias 2.0 (ROB 2) tool. This instrument evaluates potential sources of bias across five domains: the randomization process, deviations from intended interventions, missing outcome data, measurement of the outcome, and selection of the reported result. Each domain, as well as the overall risk of bias, was classified in accordance with Cochrane guidance as low risk, some concerns, or high risk.

### 2.5 Statistical analysis

The analyses of all networks meta-data were executed using Stata MP version 17.0. For outcomes that were measured using the same scale and units across various studies, we computed the mean differences (MDs) along with their respective 95% confidence intervals (CIs). In cases where the outcomes were measured on different scales or with varying units, we utilized standardized mean differences (SMDs) with their 95% CIs. The core analyses were conducted assuming consistency and employed random-effects models. The between-study variance (τ^2^) was estimated using the restricted maximum likelihood (REML) technique. In networks with closed loops, we evaluated global inconsistency through the design-by-treatment interaction model. On the other hand, we assessed local inconsistency using the node-splitting method. When the *p* value fell below 0.10, this raised a red flag about potential inconsistency. To dig deeper into loop-specific consistency, we turned to the inconsistency factor (IF); if the 95% CI for the IF included zero, we took that as a green light indicating no statistically significant disagreement between direct and indirect evidence. To bring the network geometry to life, we created network plots where the size of each node corresponded to the total sample size for a given intervention, while the thickness of the edges showed how many studies directly compared different interventions. To really separate the wheat from the chaff among competing interventions, we applied multiple ranking metrics—SUCRA, PreBest, and mean rank—which gave our results both more robustness and clearer interpretation. When we had more than 10 studies in the mix, we used comparison-adjusted funnel plots to sniff out any publication bias or small-study effects. Our sensitivity analyses employed a leave-one-out approach, where we systematically removed each study one at a time and re-estimated the random-effects consistency model, keeping a close eye on whether the direction and size of our pooled effect estimates changed—a telltale sign of result stability. Finally, we conducted univariable network meta-regression analyses to see how study-level covariates might be influencing treatment effects. The results are presented as regression coefficients complete with 95% CIs and Wald test *p* values, with anything below 0.05 hitting the statistical significance mark and suggesting a real effect-modifying association.

### 2.6 GRADE assessment

The certainty of evidence for outcomes derived from the network meta-analysis was evaluated using the GRADE framework, operationalized through the Confidence in Network Meta-Analysis (CINeMA) approach. As all included studies were randomized controlled trials, evidence certainty was initially rated as high and subsequently assessed across six domains: within-study bias, indirectness, imprecision, heterogeneity, inconsistency, and across-study bias (including publication bias and small-study effects). Within-study bias was assessed on a domain-specific basis using the RoB 2.0 tool and summarized using the CINeMA contribution matrix, which weights each study’s risk of bias according to its contribution to the corresponding network effect estimate. Indirectness was evaluated under the assumption of transitivity, with *a priori* consideration of potential effect modifiers, including baseline disease severity, intervention intensity, and follow-up duration. Imprecision was judged with reference to the minimal important difference (MID). For continuous outcomes, a standardized mean difference (SMD) threshold of 0.5 was applied, and judgments were based on the position of the 95% confidence interval relative to the null line or MID threshold. Heterogeneity was evaluated using the between-study variance (τ^2^) from random-effects models, together with the location of the prediction interval relative to the MID. When closed loops were present within the network, inconsistency between direct and indirect evidence was examined using node-splitting (SIDE) and/or design-by-treatment interaction models. To evaluate potential across-study bias, we cast a wide net by pulling together data from trial registration records, digging through grey literature, and taking a close look at comparison-adjusted funnel plots to sniff out any small-study effects. For each domain, we gave it a clean bill of health, raised some red flags, or sounded the alarm—translating to zero, one, or notches down the credibility ladder, respectively, following the GRADE playbook. In the final analysis, we slapped a high, moderate, low, or very low label on the overall certainty of evidence for each outcome.

## 3 Results

### 3.1 Systematic review and characteristics of the included studies

**Figure 1:**
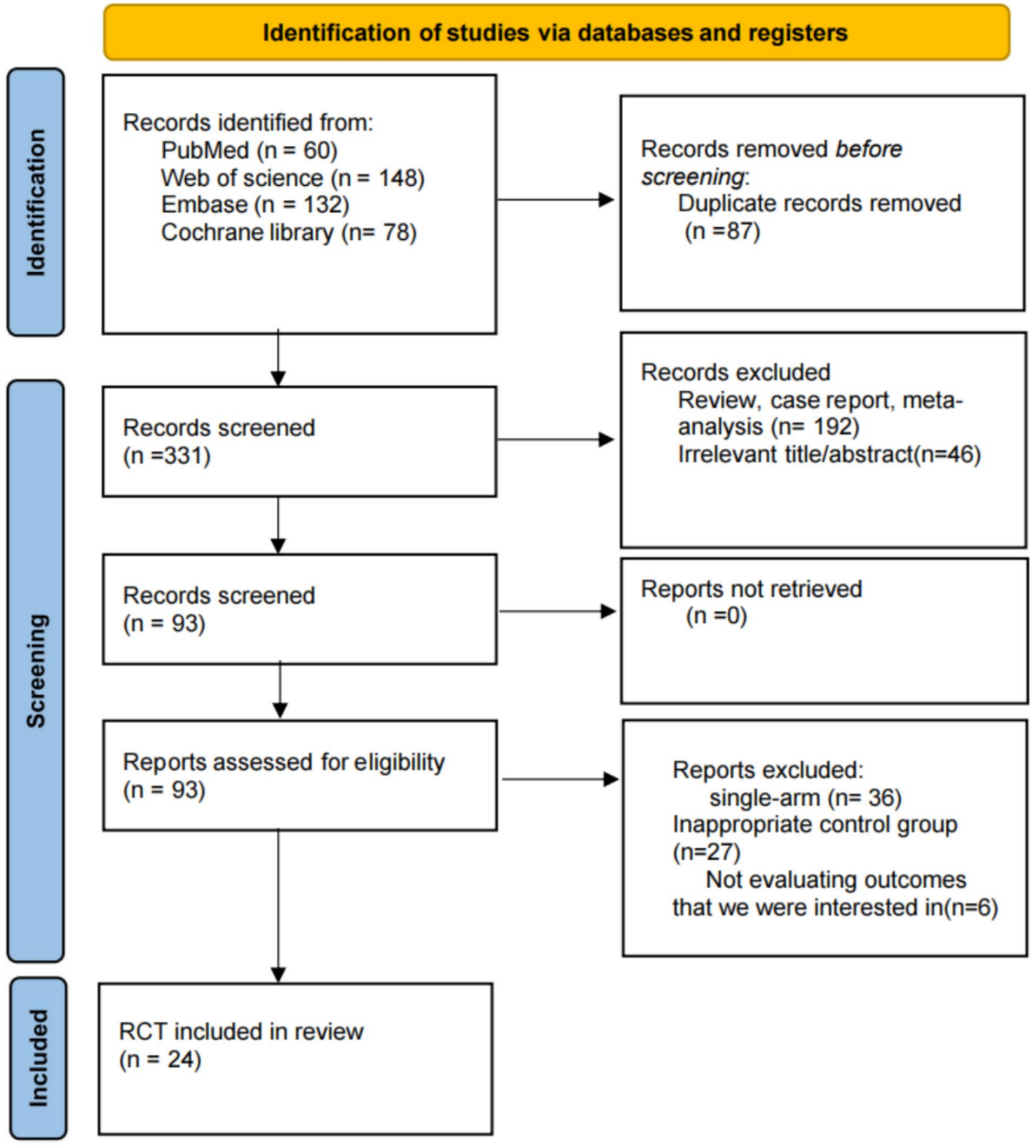
PRISMA flow diagram of study selection.

The initial literature search yielded 418 records. After removal of duplicates and screening of titles and abstracts, 331 articles proceeded to full-text assessment. Of these, 24 randomized controlled trials met the eligibility criteria and were included in the network meta-analysis (Figure 1). The included studies comprised a total of 1,298 participants and evaluated nine exercise- and nutrition-related intervention strategies: multicomponent training combined with nutritional supplementation (MC-NS), nutritional supplementation alone (NS), high-protein supplementation (HP), resistance training (RT), aerobic training (AT), combined resistance and aerobic training (RT-AT), multicomponent training (MC), resistance training combined with high-protein supplementation (RT-HP), and usual care (UC). Across the included trials, mean participant age ranged from 55 to 79.9 years. Studies were conducted in 12 countries, with a relatively high proportion originating from China. Follow-up durations ranged from 3 to 9 months. Detailed characteristics of the included studies are presented in Table 1. Overall, baseline BMI levels were broadly comparable across the included studies. (Supplementary Tables S43–S44).

**Table 1:** Characteristics of the included studies.

First author	Year	Mean age (years)	Country	Follow-up	Experimental group (*n*)	Control group (*n*)	Experimental intervention	Control intervention	Outcomes
Aubertin-Leheudre et al. (33)	2007	58	Canada	6 M	12	6	Daily supplementation with 70 mg isoflavones	Four placebo capsules daily	BMI, FM
Banitalebi et al. (34)	2021	64	Iran	3 M	32	31	Three sessions per week: 10-min warm-up, 60-min resistance training, followed by cool-down	Maintenance of usual daily activities (no regular exercise)	BMI, FM
Chen et al. (35)	2017	70	China	3 M	15/15/15	10	RT: twice weekly, 60 min/session at 60–70% 1RM (progressive loading); AT: twice weekly, 60 min/session of moderate-intensity aerobic exercise; RT + AT: once weekly each, with ≥48 h between sessions	Usual lifestyle and habitual diet; participation in structured exercise was prohibited	BMI, FM, GRIP, PBF
MEDICA (36)	2017	70	China	3 M	18	17	Three sessions per week, 55 min/session (10-min warm-up, 40-min resistance training, 5-min cool-down)	One educational lecture plus an informational booklet	BMI, FM, PBF
									SMI
Liao et al. (37)	2018	67.3	China	9 M	33	23	Three sessions per week, 55 min/session (10-min warm-up, 40-min resistance training, 5-min cool-down)	Usual lifestyle (diet and physical activity); no specific exercise intervention permitted	PBF, SMI
Mathieu et al. (38)	2016	65	Canada	4 M	8/8	10	Resistance training plus supplementation, three sessions per week, 60 min/session: post-exercise dairy-based protein (chocolate milk + milk powder) or non-dairy protein (soy-based drink + commercial EAA powder)	Usual lifestyle (diet and physical activity); no structured exercise intervention	BMI, FM
Muscariello et al. (39)	2016	65	Italy	3 M	54	50	Hypocaloric diet: 20–25 kcal/kg desirable body weight (DBW)/day with protein intake of 1.2 g/kg DBW/day	Hypocaloric diet: 20–25 kcal/kg DBW/day with protein intake of 0.8 g/kg DBW/day	BMI, FM, GRIP
Nabuco et al. (40)	2019	60	USA	4 M	13	13	Three sessions per week; ingestion of 35 g hydrolyzed whey protein immediately after resistance training	Three sessions per week; ingestion of 35 g maltodextrin after resistance training	FM, PBF
Park et al. (41)	2017	74.1	Korea	6 M	25	25	Five sessions per week, 50–80 min/session (10-min warm-up, 20–30 min resistance training, 30–50 min aerobic training, 10-min cool-down)	Usual physical activity without lifestyle modification; two sessions of health and family-life education during the intervention period	GRIP, PBF
Sammarco et al. (42)	2017	55	Italy	4 M	9	9	Protein intake of 1.2–1.4 g/kg desirable body weight (DBW)/day	Protein intake of 0.8–1.0 g/kg DBW/day	FM, PBF
Balachandran et al. (43)	2014	60	USA	3.25 M	9	8	Pneumatic resistance training, twice weekly, 55–60 min/session	Multicomponent training, twice weekly, 40–45 min/session, comprising 11 exercises	GRIP, PBF, SMI
Chiu et al. (44)	2018	79.9	China	3 M	33	31	Chair-based resistance training, twice weekly, approximately 60 min/session	Usual lifestyle and standard care	GRIP, PBF
Kim et al. (45)	2016	70	Japan	3 M	36/35/35	34	Multicomponent exercise–nutrition intervention: (1) Combined program—integrated exercise twice weekly, 60 min/session plus daily supplementation with essential amino acids (EAA) 3.0 g (leucine-enriched), vitamin D 20 μg, and tea catechins 540 mg (dissolved in 350 mL tea); (2) Exercise-only—same exercise protocol; (3) Nutrition-only—daily EAA (3 g) + vitamin D (20 μg) + tea catechins (540 mg)	Health education sessions every 2 weeks on topics of interest to older adults (e.g., cognitive function, long-term care insurance)	FM, GRIP, PBF, SMI
Liao et al. (46)	2017	67	China	3 M	25	21	Elastic-band resistance training, three sessions per week, 45–50 min/session (10-min warm-up, 35–40 min resistance training, cool-down)	Usual lifestyle: no resistance training permitted	PBF, GRIP
Banitalebi et al. (47)	2020	64	Iran	3 M	32	31	Elastic-band resistance training, three sessions per week, approximately 70 min/session (10-min warm-up, 60 min resistance training, cool-down)	Usual lifestyle; no additional structured exercise	BMI, PBF
Cunha et al. (48)	2017	68	Brazil	3 M	21/20	21	Whole-body resistance training, three sessions per week, approximately 30 min/session	No participation in any form of physical exercise during the study; maintenance of habitual lifestyle	PBF
Gadelha et al. (49)	2016	67	Brazil	6 M	69	64	Progressive resistance training, three sessions per week	Maintenance of habitual lifestyle (including diet and physical activity)	BMI, PBF
Lee et al. (50)	2021	75	China	6 M	15	12	Progressive elastic-band resistance training, three sessions per week, 55 min/session (10-min warm-up, 40-min band-based resistance training, 5-min cool-down)	Single-session health education lecture (40 min)	GRIP, PBF, SMI
Magtouf et al. (51)	2023	76	France	4 M	25	25	TMP exercise program, three sessions per week, 60 min/session	Usual lifestyle: no resistance training permitted	BMI, GRIP, PBF
Ferhi et al. (52)	2023	75	Tunisia	6 M	20	20	PSM program, twice weekly, 75 min/session (10-min warm-up, 60-min main training, 5-min cool-down)	Usual daily activities; no regular structured exercise	BMI, GRIP, PBF
Jung et al. (53)	2022	75	Korea	3 M	14	14	Multicomponent training, three sessions per week, 45–75 min/session (10-min warm-up, 25–55 min main training, 10-min cool-down)	Maintenance of usual diet and activity plus nutritional education	BMI, PBF
Hajj et al. (54)	2019	73	Lebanon	6 M	60	55	Vitamin D₃ supplementation, 30,000 IU once weekly	No specific exercise or dietary intervention	BMI, FM, GRIP, PBF
Polo-Ferrero et al. (55)	2025	77	Spain	8 M	11/14	12	Resistance training (3×/week, 50 min/session) or multicomponent training (3×/week, 50 min/session)	Continuation of usual lifestyle; advised not to initiate new structured exercise programs during the study	BMI, GRIP, PBF
Guo et la. (56)	2025	65	China	6 M	13	12	Individualized resistance training, three sessions per week, 40 min/session (5-min warm-up, 30-min main training, 5-min cool-down)	Maintenance of habitual diet and lifestyle	BMI, FM, GRIP, PBF

### 3.2 Risk of Bias assessment

**Figure 2:**
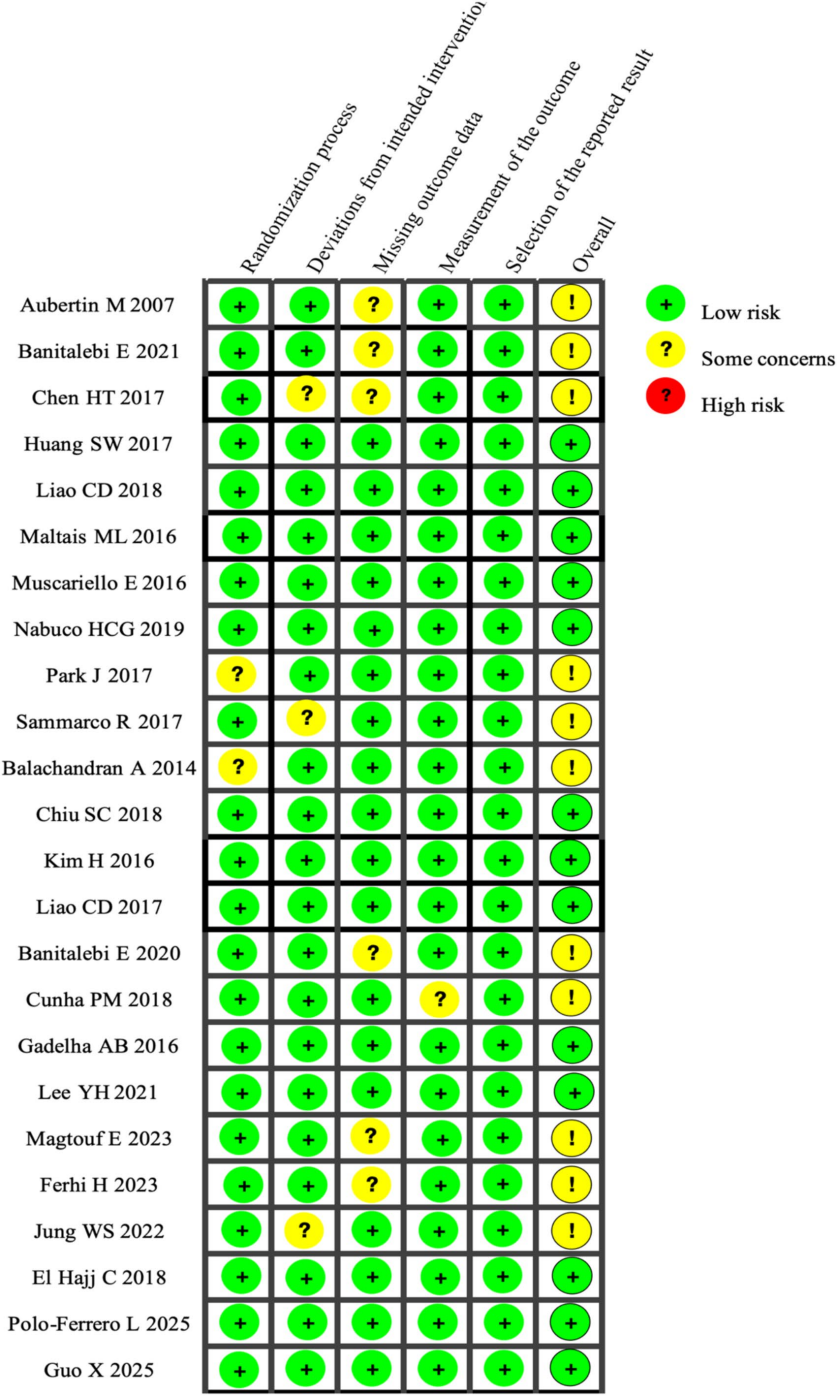
ROB2 risk of bias assessment.

Study quality was assessed using the Cochrane Risk of Bias 2.0 (ROB 2) tool. Of the 24 randomized controlled trials included, 13 were judged to be at low overall risk of bias, while 11 were rated as having some concerns. Overall, the methodological quality of the included studies was considered acceptable. Most trials reported clearly defined randomization procedures, appropriate intervention implementation, relatively complete outcome data, and objective outcome measures. With respect to the randomization process, the majority of studies described adequate allocation concealment and employed random number tables or computer-generated randomization methods. However, a small number of trials did not clearly report sequence generation or allocation concealment procedures, introducing potential selection bias. Regarding deviations from intended interventions, most studies explicitly adopted an intention-to-treat (ITT) approach, thereby reducing bias related to non-adherence or protocol deviations. Nonetheless, several trials did not implement participant blinding and relied on single-blind designs, leaving open the possibility of performance bias related to behavioral modification. When it comes to the issue of data gaps, the completeness of outcomes was mostly on point, and ITT-based strategies were often employed to tackle any missing info, which kept the threat of attrition bias to a minimum. Yet, a few studies fell short in giving the nitty-gritty details on how they dealt with data holes. As for measuring outcomes, the majority of the trials relied on objective assessment gadgets and ensured blinding at the very least during the outcome evaluation phase, which kept the chance of detection bias low. When you look at the big picture, the risk of bias across the board was quite low, and the evidence’s quality was considered up to snuff, giving us a lot of faith in the compiled findings. For a deeper dive into the risk-of-bias assessments, check out Figure 2.

### 3.3 Network meta-analyses

#### 3.3.1 Assessment of consistency and inconsistency

**Figure 3:**
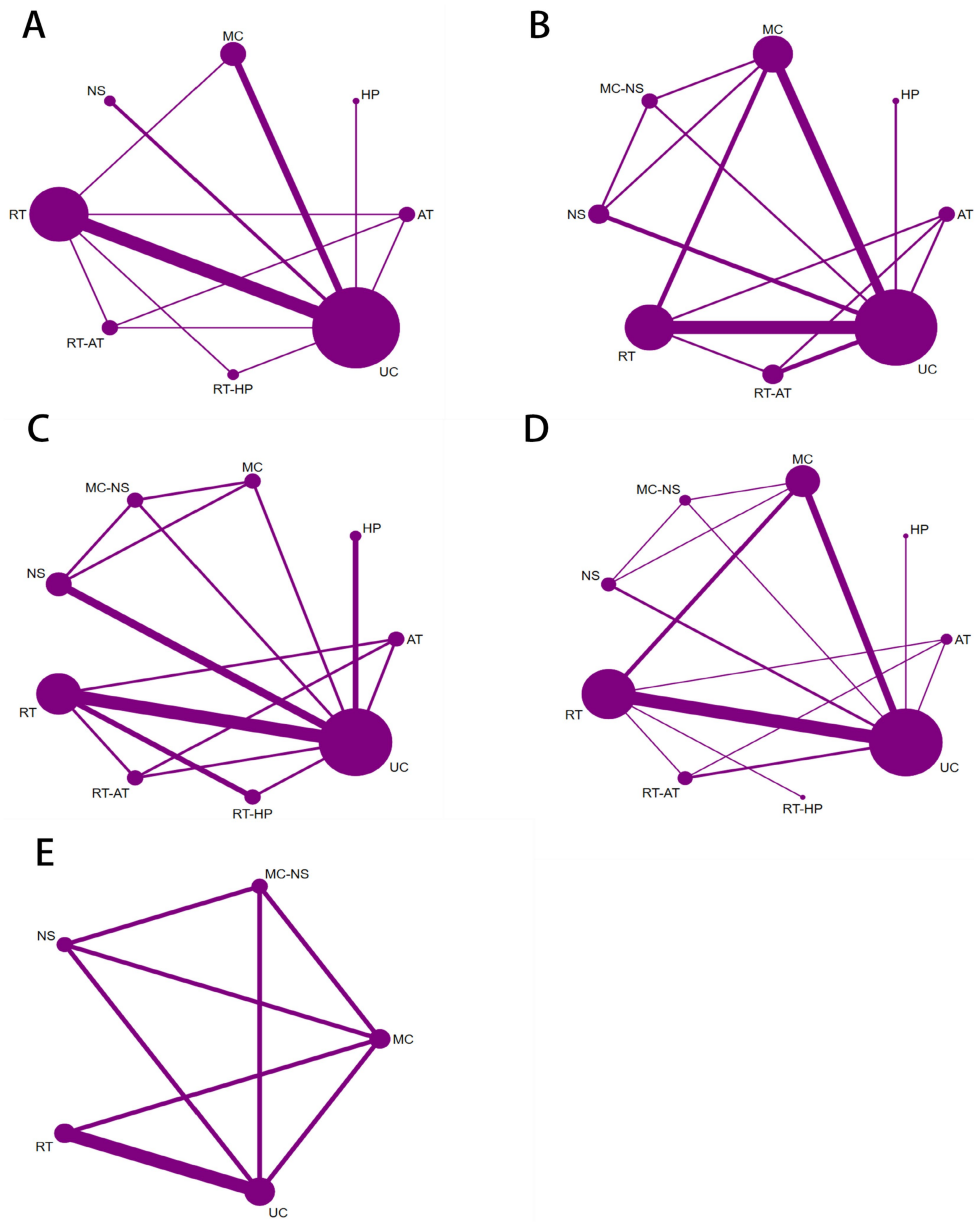
Network maps showing connections between various physical activity and dietary approaches for seniors with muscle-depleting obesity: **(A)** BMI; **(B)** GRIP; **(C)** FM; **(D)** PBF; **(E)** SMI.

In this study, SMI and GRIP were prespecified as the primary outcomes, whereas BMI, FM, and PBF were treated as secondary outcomes. For all outcomes, the corresponding network geometries formed closed loops (Figure 3), permitting formal assessment of network consistency. Global inconsistency was evaluated for each outcome, with all tests yielding *p* values > 0.05 (Supplementary Table S3), indicating that the assumption of overall network consistency was not rejected. Local inconsistency was further examined using the node-splitting approach, and none of the pairwise comparisons showed statistically significant inconsistency, with all *p* values exceeding 0.05 (Supplementary Tables S4–S8). In addition, loop-specific inconsistency analyses were conducted to assess agreement between direct and indirect evidence. For the PBF outcome, the MC–RT–UC loop produced an inconsistency factor whose confidence interval did not include zero, suggesting the presence of potential loop inconsistency. By contrast, for all other outcomes, the confidence intervals of the inconsistency factors crossed zero, providing no statistical evidence of disagreement between direct and indirect comparisons. Taken together, these findings indicate overall good consistency of the network models (Supplementary Figures S1–S5).

#### 3.3.2 BMI

**Figure 4:**
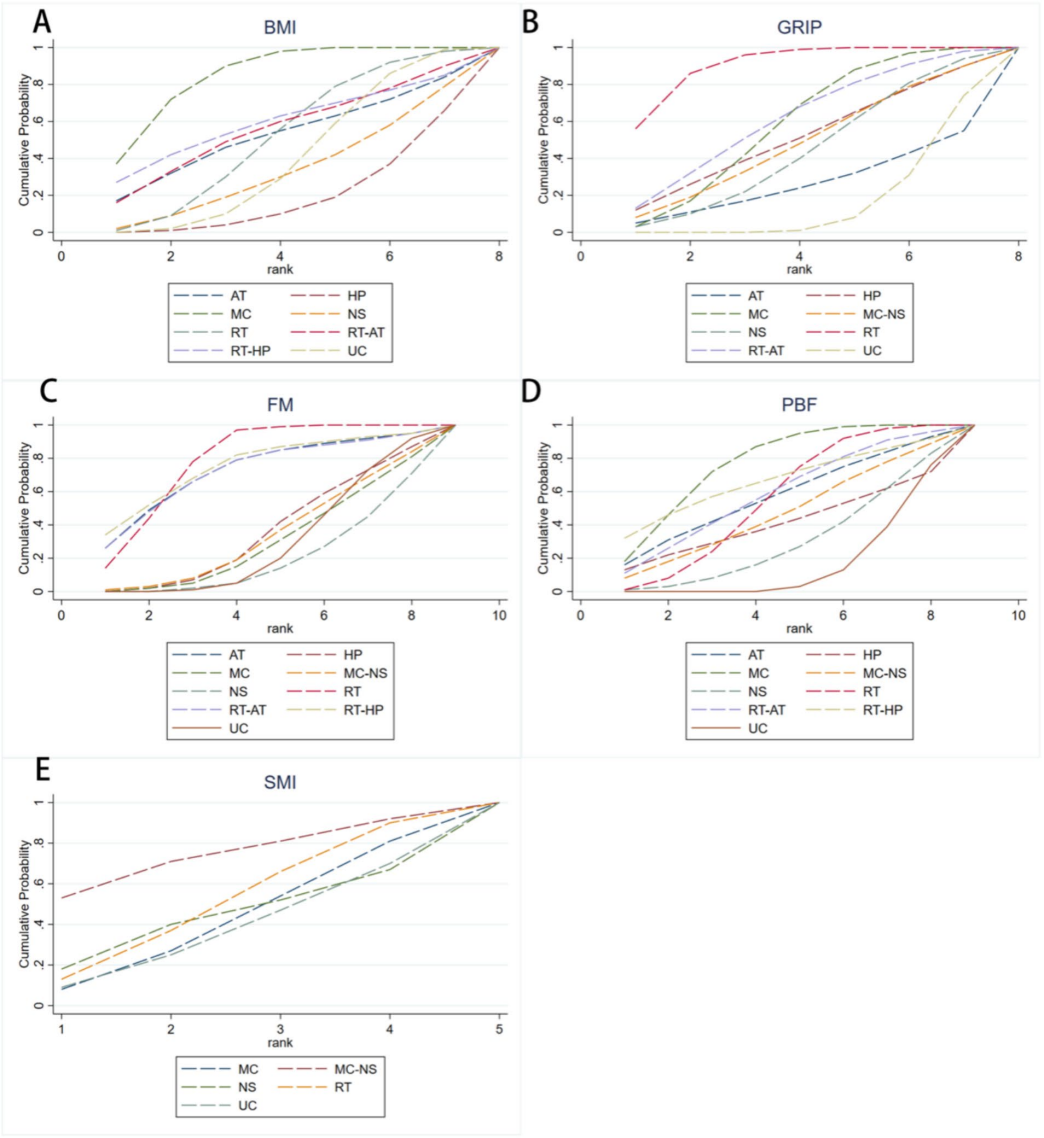
League table comparing exercise interventions, nutritional supplementation, and high-protein intake in older adults with sarcopenic obesity. Treatment effects for BMI are displayed in the lower-left triangle (yellow shading), and effects for GRIP are shown in the upper-right triangle (blue shading). The certainty of evidence is indicated according to GRADE ratings, where * denotes high certainty, & denotes moderate certainty, # denotes low certainty, and + denotes very low certainty.

**Figure 5:**
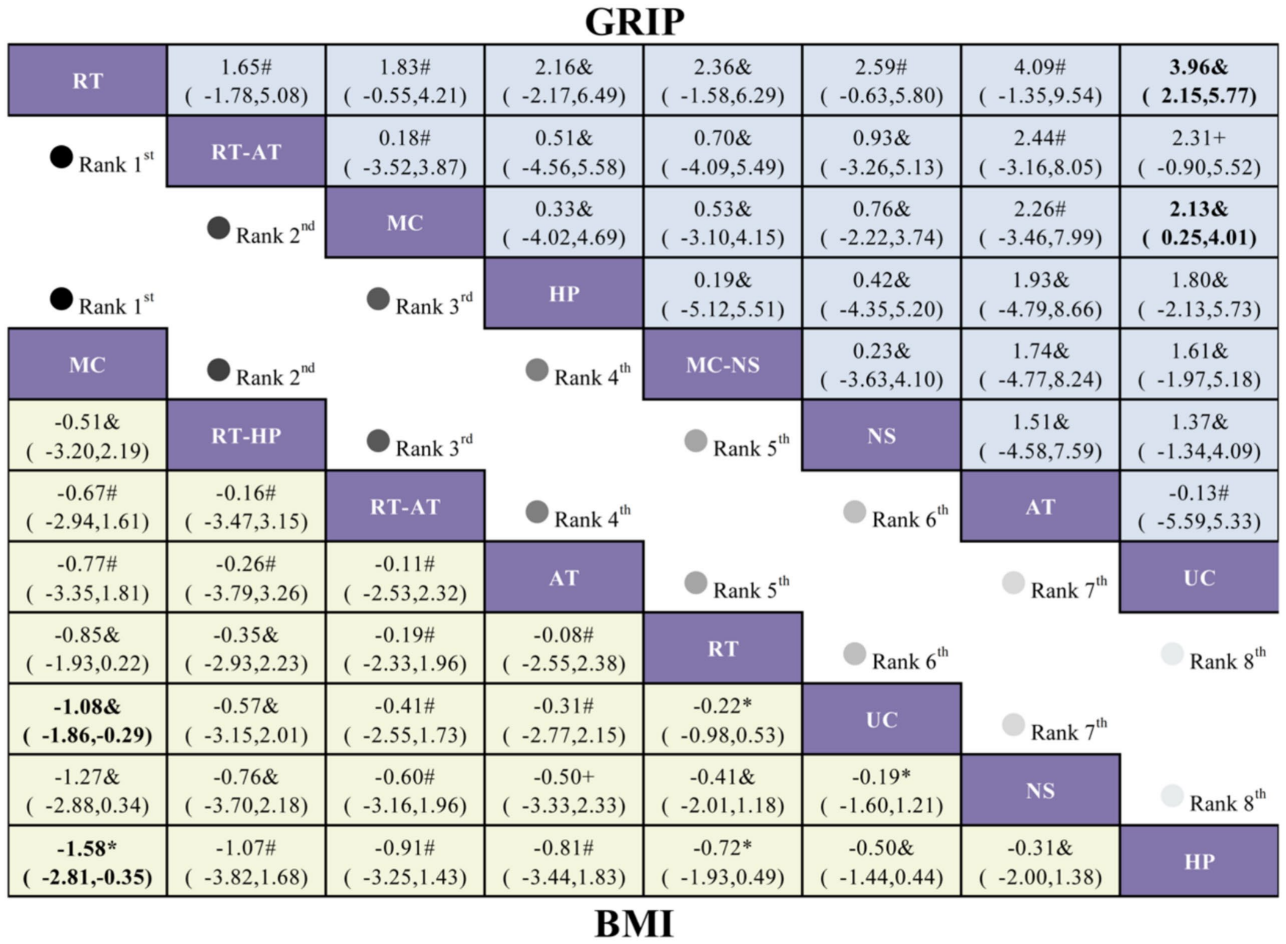
SUCRA ranking curves for exercise interventions, nutritional supplementation, and high-protein intake across outcome measures in older adults with sarcopenic obesity: **(A)** BMI; **(B)** GRIP; **(C)** FM; **(D)** PBF; **(E)** SMI.

For the BMI outcome, 13 randomized controlled trials encompassing eight intervention strategies were included (Figure 3A). Compared with UC, MC was the only intervention associated with a statistically significant reduction in BMI among older adults with sarcopenic obesity (MD = −1.08, 95% CI −1.86 to −0.29; Figure 4). RT-HP (MD = −0.57, 95% CI −3.15 to 2.01) and RT-AT (MD = −0.41, 95% CI −2.55 to 1.73) showed non-significant trends toward BMI reduction. According to SUCRA rankings, MC had the highest probability of being the most effective intervention (85.1%), followed by RT-HP (59.3%) and RT-AT (56.3%) (Figure 5; Supplementary Table S9).

#### 3.3.3 Grip

For handgrip strength, 13 randomized controlled trials involving eight intervention strategies were analyzed (Figure 3B). Compared with UC, both RT (MD = 3.96, 95% CI 2.15–5.77) and MC (MD = 2.13, 95% CI 0.25–4.01) significantly improved GRIP in older adults with sarcopenic obesity (Figure 4). Although RT-AT (MD = 2.31, 95% CI −0.90 to 5.52) and HP (MD = 1.80, 95% CI −2.13 to 5.73) were associated with numerical improvements, these effects did not reach statistical significance. SUCRA-based ranking indicated that RT was most likely to be the optimal intervention for improving GRIP (SUCRA = 90.9%), followed by RT-AT (62.1%) and MC (59.3%) (Figure 5; Supplementary Table S10).

#### 3.3.4 FM

**Figure 6:**
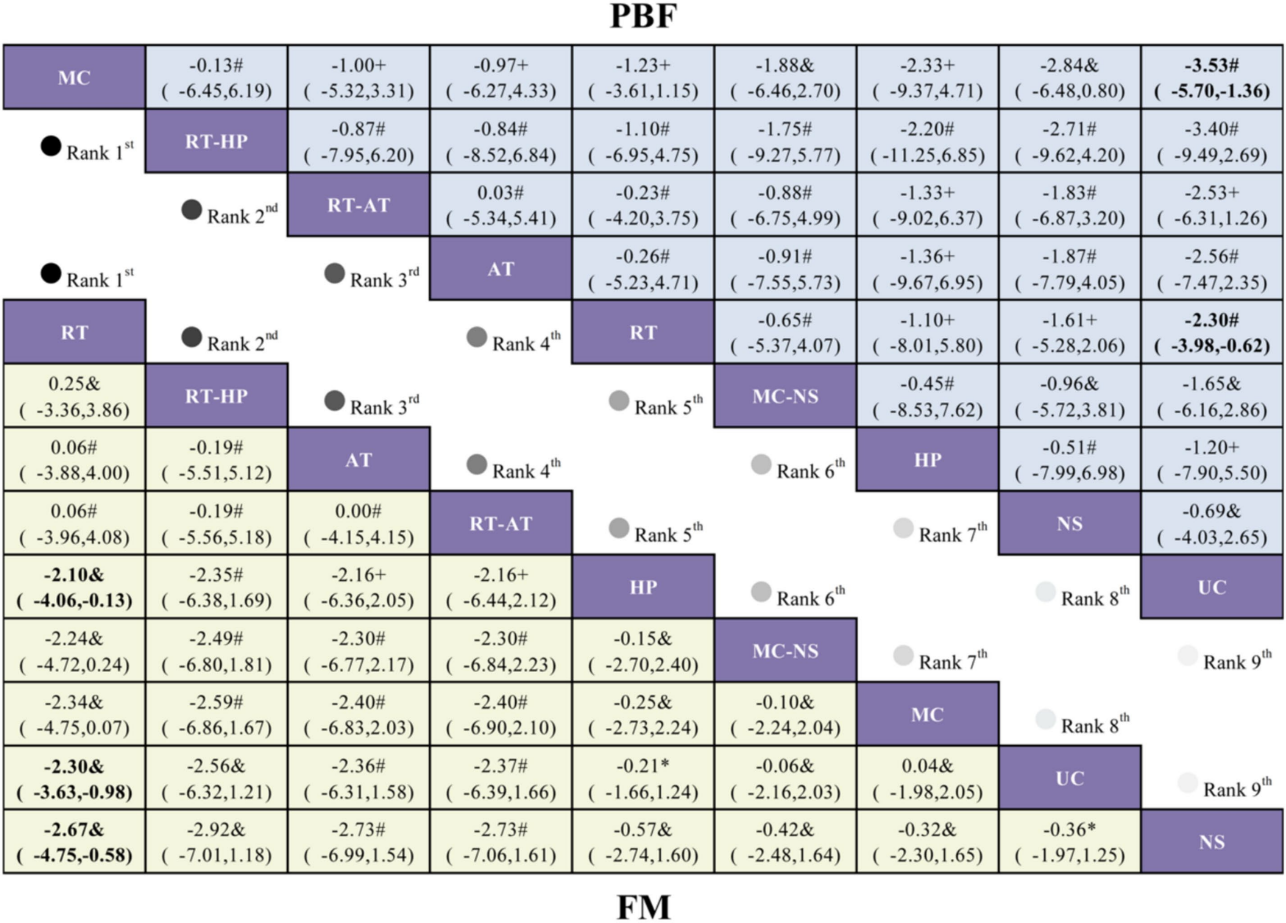
League table comparing exercise interventions, nutritional supplementation, and high-protein intake in older adults with sarcopenic obesity. Treatment effects for FM are presented in the lower-left triangle (yellow shading), while effects for PBF are shown in the upper-right triangle (blue shading). The certainty of evidence, assessed using the GRADE framework, is denoted as follows: * high certainty, & moderate certainty, # low certainty, and + very low certainty.

For the FM outcome, 11 randomized controlled trials evaluating nine intervention strategies were included (Figure 3C). Compared with UC, only RT was associated with a statistically significant reduction in fat mass (MD = −2.30, 95% CI −3.63 to −0.98; Figure 6). Although RT-HP (MD = −2.56, 95% CI −6.32 to 1.21) and aerobic training (AT) (MD = −2.36, 95% CI −6.31 to 1.58) showed favorable trends, these differences were not statistically significant. SUCRA rankings showed that RT ranked highest (79.0%), followed by RT-HP (75.5%) and AT (72.5%) (Figure 5; Supplementary Table S11).

#### 3.3.5 PBF

For percentage body fat, 20 randomized controlled trials assessing nine intervention strategies were included (Figure 3D). Compared with UC, both MC (MD = −3.53, 95% CI −5.70 to −1.36) and RT (MD = −2.30, 95% CI −3.98 to −0.62) resulted in statistically significant reductions in PBF (Figure 6). In contrast, RT-HP (MD = −3.40, 95% CI −9.49 to 2.69) and RT-AT (MD = −2.53, 95% CI −6.31 to 1.26) were associated with non-significant reductions. Based on SUCRA values, MC was most likely to be the optimal intervention (77.1%), followed by RT-HP (66.3%) and RT-AT (58.6%) (Figure 5; Supplementary Table S12).

#### 3.3.6 SMI

**Figure 7:**
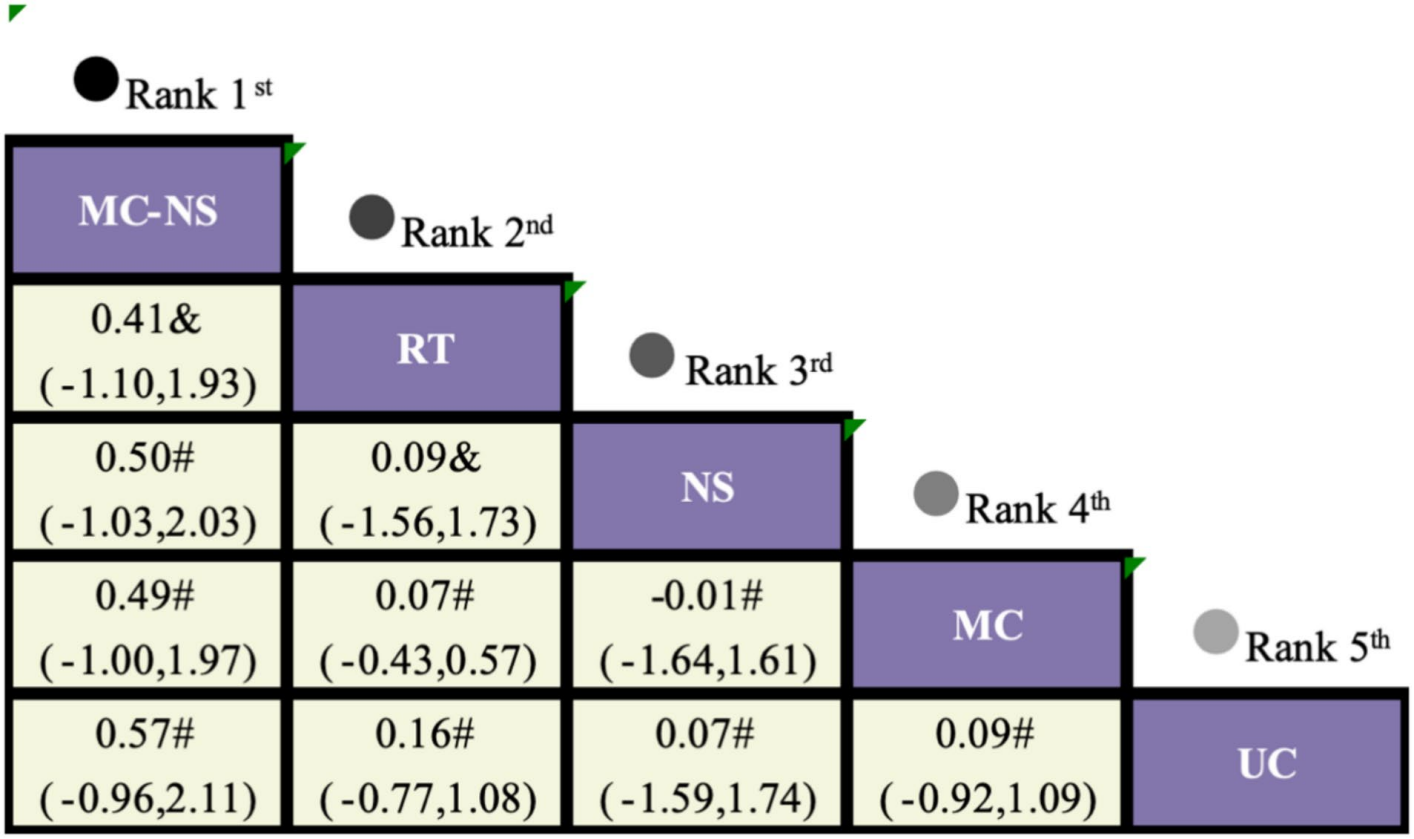
League table comparing exercise interventions, nutritional supplementation, and high-protein intake for SMI in older adults with sarcopenic obesity. The certainty of evidence, assessed according to the GRADE framework, is indicated as follows: * high certainty, & moderate certainty, # low certainty, and + very low certainty.

For the SMI outcome, five randomized controlled trials involving five intervention strategies were included (Figure 3E). Compared with UC, MC-NS showed a non-significant increase in SMI (MD = 0.41, 95% CI −1.10 to 1.93; Figure 7). SUCRA-based ranking suggested that MC-NS had the highest probability of being the most effective intervention (SUCRA = 74.1%), followed by RT (51.4%) and NS alone (44.1%) (Figure 5; Supplementary Table S13).

### 3.4 Sensitivity analysis, meta-regression, and publication bias

To assess the impact of individual studies on the pooled effect estimates, we performed leave-one-out sensitivity analyses across all outcomes. This approach involved systematically removing one study at a time and re-running the network meta-analysis on the remaining data under a random-effects model while maintaining the consistency assumption throughout each iteration. Across all analyses, the direction of pooled effects for exercise and nutritional interventions relative to UC remained unchanged. Variations in effect size magnitude were modest, confidence intervals overlapped substantially, and inferences regarding statistical significance were not materially altered, indicating robustness of the findings (Supplementary Tables S14–S18). To explore potential sources of heterogeneity, univariable network meta-regression analyses were performed for the five outcomes, with geographic region, follow-up duration, and mean participant age included as study-level covariates. None of these covariates showed a statistically significant association with the relative treatment effects of any intervention compared with UC (Supplementary Tables S19–S33).

Moreover, we crafted funnel plots that accounted for comparisons to evaluate BMI, grip strength, forearm muscle, percentage body fat, and skeletal muscle index, as well as overall effectiveness. A quick glance revealed a roughly balanced spread with no striking anomalies, indicating a slim chance of significant publication bias (refer to Supplementary Figures S6–S10).

### 3.5 GRADE assessment

The certainty of evidence for the primary outcomes was evaluated using the CINeMA framework, revealing variability across outcomes. Detailed evaluations are provided in Supplementary Tables S34–S38. For BMI, among the 28 pairwise comparisons, 4 (14%) were rated as high certainty, 10 (36%) as moderate certainty, 13 (46%) as low certainty, and 1 (4%) as very low certainty. For GRIP, also across 28 comparisons, 19 (68%) were judged moderate certainty, 8 (28%) low certainty, and 1 (4%) very low certainty. For FM (36 comparisons), 2 (6%) were high certainty, 16 (44%) moderate certainty, 16 (44%) low certainty, and 2 (6%) very low certainty. The overall certainty for PBF (36 comparisons) was comparatively lower, with 5 (14%) rated moderate certainty, 21 (58%) low certainty, and 10 (28%) very low certainty. For SMI (10 comparisons), 2 (20%) were moderate certainty, while the remaining 8 (80%) were low certainty.

## 4 Discussion

This network meta-analysis synthesized evidence from 24 randomized controlled trials involving 1,298 older adults with sarcopenic obesity, comparing nine categories of exercise- and nutrition-related interventions. Using a consistency-based random-effects network meta-analytic framework, we quantitatively compared and ranked their effects on BMI, GRIP, FM, PBF, and SMI. The included studies were conducted across 12 countries, with mean participant ages ranging from 55 to 79.9 years and follow-up durations predominantly spanning 3–9 months. Accordingly, the present findings primarily reflect short- to mid-term intervention effects.

### 4.1 Main findings and clinical interpretation

With respect to BMI, MC was the only intervention that achieved a statistically significant reduction compared with UC (MD = −1.08, 95% CI −1.86 to −0.29). Although RT-HP and RT-AT demonstrated downward trends, these effects did not reach statistical significance. This pattern suggests that, in older adults with sarcopenic obesity, single-modality exercise interventions may be insufficient to elicit consistent improvements in weight-related indices, whereas multicomponent programs—by concurrently integrating aerobic, resistance, and functional training elements—may be more likely to produce measurable reductions in BMI (22, 23), Importantly, BMI changes alone provide an incomplete representation of body composition. In sarcopenic obesity, clinically meaningful improvement is defined by fat loss accompanied by preservation or accretion of muscle mass, underscoring the necessity of interpreting BMI alongside FM, PBF, and SMI.

In terms of muscle strength, both RT and MC significantly improved handgrip strength, with RT demonstrating the larger effect size (RT: MD = 3.96, 95% CI 2.15–5.77; MC: MD = 2.13, 95% CI 0.25–4.01) and ranking highest in SUCRA analyses. These findings are biologically plausible, as resistance training enhances muscle strength through externally imposed loads, progressive overload, and varied contraction velocities, thereby promoting neuromuscular adaptation and increasing force-generating capacity (24, 25). Given the well-established associations between handgrip strength and fall risk, functional decline, and mortality in older adults, resistance training should be regarded as a foundational component of exercise prescriptions for individuals with sarcopenic obesity.

For adiposity-related outcomes, the FM analysis indicated that only RT, relative to UC, produced a statistically significant reduction in fat mass (MD = −2.30, 95% CI −3.63 to −0.98) and ranked prominently among competing interventions. In contrast, for PBF, both MC and RT were associated with significant reductions (MC: MD = −3.53, 95% CI −5.70 to −1.36; RT: MD = −2.30, 95% CI −3.98 to −0.62). These results suggest that both intervention types may meaningfully contribute to reductions in relative adiposity. From a mechanistic standpoint, RT may decrease fat mass indirectly by increasing lean tissue and improving metabolic efficiency, whereas MC may exert broader effects on body fat percentage through its more comprehensive and functionally oriented training structure (22, 23, 26). Nevertheless, the detection of a potential inconsistency within the MC–RT–UC closed loop in the PBF network necessitates caution when interpreting rankings and effect estimates for this outcome. Accordingly, the certainty of evidence for PBF was downgraded in the network GRADE assessment. To explore potential sources of this inconsistency, a clinical heterogeneity assessment was undertaken focusing on exercise intensity and baseline participant characteristics across studies contributing to the MC–RT–UC loop. Although interventions were broadly categorized as resistance training or multicomponent training, substantial variability existed in prescribed exercise intensity. Some RT and MC programs employed moderate-intensity protocols according to ACSM criteria (e.g., RPE 12–13 or 40–60% 1RM), whereas others adopted moderate-to-vigorous or high-intensity regimens (e.g., ≥70–80% 1RM or higher perceived exertion), potentially leading to differential effects on body fat percentage. In addition, baseline BMI distribution varied markedly across studies, ranging from overweight or mildly obese populations (mean BMI ≈ 25–27 kg/m^2^) to individuals with established obesity (mean BMI ≥ 33–35 kg/m^2^). Given that baseline adiposity level may modify the responsiveness of PBF to exercise interventions, such heterogeneity in participant characteristics may violate the transitivity assumption and partly explain the observed inconsistency within the MC–RT–UC closed loop for PBF outcomes.

Evidence regarding muscle mass outcomes was comparatively limited. Only five trials reported SMI, and although MC-NS showed a non-significant trend toward increasing SMI relative to UC (MD = 0.41, 95% CI −1.10 to 1.93) and ranked favorably, the available evidence does not support definitive conclusions (27). Improvements in skeletal muscle mass typically require longer intervention durations, adequate and sustained protein intake, and progressively increasing training loads. The short follow-up periods, modest sample sizes, sparse network connections, and imprecision of effect estimates in the included trials likely contributed to the absence of statistically significant findings for SMI. In the present network meta-analysis, evidence regarding changes in SMI was limited, and none of the evaluated interventions produced statistically significant improvements in this outcome. Although MC-NS ranked favorably and showed a non-significant upward trend in SMI relative to usual care, the magnitude and robustness of this effect remain uncertain. These findings are likely attributable to a combination of physiological and methodological factors. A principal biological explanation is age-related anabolic resistance, a well-recognized phenomenon whereby older adults exhibit a blunted skeletal muscle protein synthesis response to both resistance exercise and protein intake (28). Accordingly, in older adults with sarcopenic obesity, the protein doses used in many trials may have been insufficient to overcome this physiological barrier (29). Moreover, prior meta-analyses have indicated that longer intervention durations and sufficiently high training loads are critical for inducing muscle hypertrophy, whereas the relatively short intervention periods and heterogeneity in training protocols across most included trials may have limited the detectability of changes in SMI (30).

### 4.2 Relationship to previous Meta-analyses

The results of this study clearly demonstrate that older adults battling sarcopenic obesity can significantly boost their handgrip strength and trim down their body fat percentage by engaging in exercise programs, with resistance training proving to be particularly effective in this regard. These results are broadly consistent with those reported by Chen et al., who likewise observed favorable effects of exercise on muscle strength and adiposity-related outcomes (31). However, their meta-analysis included only 12 trials and focused exclusively on resistance training, without considering alternative exercise modalities. In contrast, the current network meta-analysis incorporated a wider spectrum of exercise-based strategies and explicitly evaluated the potential modifying roles of nutritional supplementation and high-protein intake. This broader analytical framework enabled a more comprehensive comparison of rehabilitation approaches for sarcopenic obesity. Similarly, Contillo et al. reported that exercise interventions were associated with reductions in body fat and improvements in handgrip strength (32). Nevertheless, their analysis did not distinguish the relative effectiveness of specific exercise modalities. By applying a network approach with SUCRA-based ranking, the present study extends prior work by demonstrating that resistance training is most consistently associated with superior outcomes in both adiposity reduction and muscle strength enhancement in this population. In the meta-analysis conducted by Contillo et al., interventions were primarily categorized according to exercise modality (AT, RT, RT-AT) and the presence or absence of concomitant protein or amino acid supplementation. Building on this framework, the present network meta-analysis extends the scope of comparative evaluation by incorporating not only direct contrasts between different exercise modalities, but also a systematic comparison of multicomponent training combined with nutritional supplementation against alternative intervention strategies. This expanded approach allows for a more comprehensive assessment of the relative effectiveness of integrated intervention models. With respect to outcome measures, the two analyses demonstrate substantial concordance, with both focusing predominantly on muscle strength and body composition outcomes, including handgrip strength, skeletal muscle mass, fat mass, and percentage body fat. These measures are widely regarded as core clinical endpoints for evaluating intervention efficacy in sarcopenic obesity. From a methodological perspective, the present network meta-analysis further strengthens the interpretability of its findings by applying the GRADE framework to systematically rate the certainty of evidence across key outcomes. This approach enhances transparency, improves clinical credibility, and provides a structured hierarchy of evidence to better inform clinical decision-making.

### 4.3 Strengths and implications for clinical practice

When the totality of outcomes is considered, the present findings suggest that resistance training yields more consistent benefits for improving GRIP and reducing FM, whereas multicomponent training appears more advantageous for improving BMI and PBF. Given the consistent advantages of MC in reducing BMI and PBF, it is clinically appropriate to translate these findings into practical recommendations. For older adults with sarcopenic obesity, MC may reasonably integrate aerobic and resistance training within a single intervention framework, with each component accounting for approximately 40–60% of the total training volume to maximize their complementary effects. Aerobic exercise may be prescribed at moderate intensity (approximately 60–75% of maximal heart rate) for 20–30 min per session to enhance energy expenditure and promote fat loss, whereas resistance training may be performed 2–3 times per week using progressive loads corresponding to roughly 60–80% of one-repetition maximum, targeting major muscle groups. In clinical practice, MC programs should be individualized according to baseline functional status and comorbid conditions, and progressively advanced to ensure both safety and adequate training stimulus. MC may be particularly appropriate for individuals in whom adiposity reduction is a primary therapeutic objective, whereas resistance training alone should remain the cornerstone intervention for patients with pronounced muscle weakness or functional impairment. From a clinical perspective, resistance training may therefore be regarded as a foundational intervention, particularly for older adults with sarcopenic obesity who exhibit pronounced muscle weakness or functional impairment. Aerobic and functional components may be incorporated as appropriate to augment energy expenditure and enhance performance in activities of daily living.

Nutritional supplementation and high-protein intake are biologically plausible adjuncts for preserving or augmenting muscle mass; however, substantial heterogeneity exists across studies with respect to supplement type, dosage, adherence, and dietary context. As a result, the current evidence base supports cautious consideration rather than strong clinical endorsement of these strategies. Exercise prescriptions should also be tailored to individual comorbidity profiles and risk factors—such as osteoporosis, knee or hip osteoarthritis, and cardiopulmonary limitations—and implemented with graded intensity, appropriate exercise selection, and safety monitoring, preferably under professional supervision.

The strengths of this study include its focus on sarcopenic obesity as a distinct and clinically burdensome phenotype, the inclusion of a relatively large number of trials spanning multiple countries, and the use of network meta-analysis to enable systematic comparison and ranking of multiple interventions. We further conducted comprehensive assessments of consistency and inconsistency, along with leave-one-out sensitivity analyses and network meta-regression, to confirm the robustness of the findings. Evaluation of evidence certainty using the CINeMA framework revealed substantial variability across outcomes, with particularly low certainty for PBF and SMI, underscoring the need for cautious interpretation of these results.

### 4.4 Limitations

Several limitations merit consideration. First, strict double blinding is inherently difficult in exercise-based interventions, potentially introducing performance and detection bias; however, most primary outcomes were objectively measured, which likely mitigated this concern. Second, some comparisons were limited by small sample sizes and sparse network connectivity, particularly for SMI, which was reported in only five trials, resulting in imprecise effect estimates. Third, heterogeneity in exercise intensity, training frequency, and nutritional supplementation protocols may have influenced the transitivity assumption and the reliability of indirect comparisons. Fourth, the detection of potential loop inconsistency within the PBF network highlights the need for additional head-to-head trials to clarify these relationships. Finally, most studies had follow-up durations of 3–9 months, restricting assessment of long-term sustainability, adverse events, and adherence; moreover, safety and compliance were not consistently reported.

## 5 Conclusion

In summary, among older adults with sarcopenic obesity, resistance training demonstrates the most consistent benefits for improving handgrip strength and reducing fat mass, whereas multicomponent training appears more effective for improving BMI and PBF. MC-NS may offer potential benefits for increasing muscle mass, but current evidence remains insufficient to support definitive conclusions. Larger, well-designed randomized controlled trials with standardized outcome definitions, longer follow-up periods, and direct head-to-head comparisons of key interventions are needed to strengthen the certainty of evidence and to inform optimal rehabilitation strategies for this growing and clinically important population.

## Data Availability

The original contributions presented in the study are included in the article/Supplementary material, further inquiries can be directed to the corresponding author.
