# Supplementary material for: Comparative effectiveness of exercise modalities and nutritional supplementation for sarcopenic obesity in older adults: a network meta-analysis based on randomized controlled trials
Source: Front Public Health. 2026 Feb 23;14:1775783. doi: 10.3389/fpubh.2026.1775783 (PMC12967945; doi:10.3389/fpubh.2026.1775783)
Supplement: Supplementary file 1 [file Supplementary_file_1.docx]

Comparative Effectiveness of Exercise Modalities and Nutritional Supplementation for Sarcopenic Obesity in Elderly: A Network Meta-Analysis Based on Randomized Controlled Trials

| **Table of Contents** | | |
| --- | --- | --- |
| Title | Content | page |
| Table S1 | PRISMA NMA Checklist of Items to Include When Reporting a Systematic Review Involving a Network Meta-analysis | 2-5 |
| Table S2 | Literature Search Strategy | 5-11 |
| Table S3 | Global inconsistency table for BMI, GRIP, FM, PBF, and SMI outcomes in older adults with sarcopenic obesity | 11 |
| Table S4 | Node-splitting analysis for the BMI outcome in older adults with sarcopenic obesity | 11 |
| Table S5 | Node-splitting analysis for the GRIP outcome in older adults with sarcopenic obesity | 12 |
| Table S6 | Node-splitting analysis for the FM outcome in older adults with sarcopenic obesity | 12 |
| Table S7 | Node-splitting analysis for the PBF outcome in older adults with sarcopenic obesity | 13 |
| Table S8 | Node-splitting analysis for the SMI outcome in older adults with sarcopenic obesity | 13 |
| Figure S1 | Loop inconsistency plot for the BMI outcome in older adults with sarcopenic obesity | 14 |
| Figure S2 | Loop inconsistency plot for the GRIP outcome in older adults with sarcopenic obesity | 14 |
| Figure S3 | Loop inconsistency plot for the FM outcome in older adults with sarcopenic obesity | 15 |
| Figure S4 | Loop inconsistency plot for the PBF outcome in older adults with sarcopenic obesity | 15 |
| Figure S5 | Loop inconsistency plot for the SMI outcome in older adults with sarcopenic obesity | 16 |
| Table S9 | SUCRA ranking table for the BMI outcome in older adults with sarcopenic obesity | 16 |
| Table S10 | SUCRA ranking table for the GRIP outcome in older adults with sarcopenic obesity | 16 |
| Table S11 | SUCRA ranking table for the FM outcome in older adults with sarcopenic obesity | 17 |
| Table S12 | SUCRA ranking table for the PBF outcome in older adults with sarcopenic obesity | 17 |
| Table S13 | SUCRA ranking table for the SMI outcome in older adults with sarcopenic obesity | 17 |
| Table S14 | Sensitivity analysis for the BMI outcome in older adults with sarcopenic obesity | 18-20 |
| Table S15 | Sensitivity analysis for the GRIP outcome in older adults with sarcopenic obesity | 20-22 |
| Table S16 | Sensitivity analysis for the FM outcome in older adults with sarcopenic obesity | 22-24 |
| Table S17 | Sensitivity analysis for the PBF outcome in older adults with sarcopenic obesity | 24-28 |
| Table S18 | Sensitivity analysis for the SMI outcome in older adults with sarcopenic obesity | 28-29 |
| Table S19 | Meta-regression analysis of the BMI outcome with country as a moderator in older adults with sarcopenic obesity | 29 |
| Table S20 | Meta-regression analysis of the BMI outcome with follow-up duration as a moderator in older adults with sarcopenic obesity | 29-30 |
| Table S21 | Meta-regression analysis of the BMI outcome with mean age as a moderator in older adults with sarcopenic obesity | 30 |
| Table S22 | Meta-regression analysis of the GRIP outcome with country as a moderator in older adults with sarcopenic obesity | 30 |
| Table S23 | Meta-regression analysis of the GRIP outcome with follow-up duration as a moderator in older adults with sarcopenic obesity | 31 |
| Table S24 | Meta-regression analysis of the GRIP outcome with mean age as a moderator in older adults with sarcopenic obesity | 31 |
| Table S25 | Meta-regression analysis of the FM outcome with country as a moderator in older adults with sarcopenic obesity | 32 |
| Table S26 | Meta-regression analysis of the FM outcome with follow-up duration as a moderator in older adults with sarcopenic obesity | 32 |
| Table S27 | Meta-regression analysis of the FM outcome with mean age as a moderator in older adults with sarcopenic obesity | 33 |
| Table S28 | Meta-regression analysis of the PBF outcome with country as a moderator in older adults with sarcopenic obesity | 33 |
| Table S29 | Meta-regression analysis of the PBF outcome with follow-up duration as a moderator in older adults with sarcopenic obesity | 34 |
| Table S30 | Meta-regression analysis of the PBF outcome with mean age as a moderator in older adults with sarcopenic obesity | 34-35 |
| Table S31 | Meta-regression analysis of the SMI outcome with country as a moderator in older adults with sarcopenic obesity | 35 |
| Table S32 | Meta-regression analysis of the SMI outcome with follow-up duration as a moderator in older adults with sarcopenic obesity | 35 |
| Table S33 | Meta-regression analysis of the SMI outcome with mean age as a moderator in older adults with sarcopenic obesity | 35 |
| Figure S6 | Comparison-adjusted funnel plot for the BMI outcome in older adults with sarcopenic obesity | 36 |
| Figure S7 | Comparison-adjusted funnel plot for the GRIP outcome in older adults with sarcopenic obesity | 36 |
| Figure S8 | Comparison-adjusted funnel plot for the FM outcome in older adults with sarcopenic obesity | 37 |
| Figure S9 | Comparison-adjusted funnel plot for the PBF outcome in older adults with sarcopenic obesity | 37 |
| Figure S10 | Comparison-adjusted funnel plot for the SMI outcome in older adults with sarcopenic obesity | 38 |
| Table S34 | GRADE certainty of evidence for the BMI outcome in older adults with sarcopenic obesity | 38-39 |
| Table S35 | GRADE certainty of evidence for the GRIP outcome in older adults with sarcopenic obesity | 39-40 |
| Table S36 | GRADE certainty of evidence for the FM outcome in older adults with sarcopenic obesity | 40-41 |
| Table S37 | GRADE certainty of evidence for the PBF outcome in older adults with sarcopenic obesity | 41-42 |
| Table S38 | GRADE certainty of evidence for the SMI outcome in older adults with sarcopenic obesity | 42 |
| Table S39 | Exercise intensity and baseline BMI varied across the included studies in older adults with sarcopenic obesity | 43-44 |

Table S1 PRISMA NMA Checklist of Items to Include When Reporting a Systematic Review Involving a Network Meta-analysis

| Section/Topic | Item # | Checklist Item | Reported on Page # |
| --- | --- | --- | --- |
| TITLE |  |  |  |
| Title | 1 | Identify the report as a systematic review *incorporating*  anetwork meta-analysis (or related form of meta-analysis). | 1 |
|  |  |  |  |
| ABSTRACT |  |  |  |
| Structured summary | 2 | Provide a structured summary including, as applicable:  **Background:** main objectives  **Methods:** data sources; study eligibility criteria, participants, and interventions; study appraisal; and *synthesis methods, such as network meta-analysis.*  **Results:** number of studies and participants identified; summary estimates with corresponding confidence/credible intervals; treatment rankings may also be discussed. Authors may choose to summarize pairwise comparisons against a chosen treatment included in their analyses for brevity.  **Discussion/Conclusions:** limitations; conclusions and implications of findings.  **Other:** systematic review registration number with registry name. | 1-2 |
|  |  |  |  |
| INTRODUCTION |  |  |  |
| Rationale | 3 | Describe the rationale for the review in the context of what is already known*, including mention of why a network meta-analysis has been conducted.* | 2 |
| Objectives | 4 | Provide an explicit statement of questions being addressed, with reference to participants, interventions, comparisons, outcomes, and study design (PICOS). | 3 |
|  |  |  |  |
| METHODS |  |  |  |
| Protocol and registration | 5 | Indicate whether a review protocol exists and if and where it can be accessed (e.g., Web address); and, if available, provide registration information, including registration number. | 3 |
| Eligibility criteria | 6 | Specify study characteristics (e.g., PICOS, length of follow-up) and report characteristics (e.g., years considered, language, publication status) used as criteria for eligibility, giving rationale. *Clearly describe eligible treatments included in the treatment network, and note whether any have been clustered or merged into the same node (with justification).* | 3-4 |
| Information sources | 7 | Describe all information sources (e.g., databases with dates of coverage, contact with study authors to identify additional studies) in the search and date last searched. | 4 |
| Search | 8 | Present full electronic search strategy for at least one database, including any limits used, such that it could be repeated. | 3，Supplementary  TableS2 |
| Study selection | 9 | State the process for selecting studies (i.e., screening, eligibility, included in systematic review, and, if applicable, included in the meta-analysis). | 3-4，Table 1 |
| Data collection process | 10 | Describe method of data extraction from reports (e.g., piloted forms, independently, in duplicate) and any processes for obtaining and confirming data from investigators. | 4 |
| Data items | 11 | List and define all variables for which data were sought (e.g., PICOS, funding sources) and any assumptions and simplifications made. | 3-4 |
| Geometry of the network | S1 | Describe methods used to explore the geometry of the treatment network under study and potential biases related to it. This should include how the evidence base has been graphically summarized for presentation, and what characteristics were compiled and used to describe the evidence base to readers. | 4 |
| Risk of bias within individual studies | 12 | Describe methods used for assessing risk of bias of individual studies (including specification of whether this was done at the study or outcome level), and how this information is to be used in any data synthesis. | 5 |
| Summary measures | 13 | State the principal summary measures (e.g., risk ratio, difference in means). Also describe the use of additional summary measures assessed, such as treatment rankings and surface under the cumulative ranking curve (SUCRA) values, as well as modified approaches used to present summary findings from meta-analyses. | 5 |
| Planned methods of analysis | 14 | Describe the methods of handling data and combining results of studies for each network meta-analysis. This should include, but not be limited to:   - Handling of multi-arm trials; - Selection of variance structure; - Selection of prior distributions in Bayesian analyses; and - Assessment of model fit. | 5 |
| Assessment of Inconsistency | S2 | Describe the statistical methods used to evaluate the agreement of direct and indirect evidence in the treatment network(s) studied. Describe efforts taken to address its presence when found. | 5 |
| Risk of bias across studies | 15 | Specify any assessment of risk of bias that may affect the cumulative evidence (e.g., publication bias, selective reporting within studies). | 5 |
| Additional analyses | 16 | Describe methods of additional analyses if done, indicating which were pre-specified. This may include, but not be limited to, the following:   - Sensitivity or subgroup analyses; - Meta-regression analyses; - Alternative formulations of the treatment network; and - Use of alternative prior distributions for Bayesian analyses (if applicable). | 5 |
| RESULTS† |  |  |  |
| Study selection | 17 | Give numbers of studies screened, assessed for eligibility, and included in the review, with reasons for exclusions at each stage, ideally with a flow diagram. | 6-8，Fig1 |
| Presentation of network structure | S3 | Provide a network graph of the included studies to enable visualization of the geometry of the treatment network. | 11，14，Fig3and 6 |
| Summary of network geometry | S4 | Provide a brief overview of characteristics of the treatment network. This may include commentary on the abundance of trials and randomized patients for the different interventions and pairwise comparisons in the network, gaps of evidence in the treatment network, and potential biases reflected by the network structure. | 10-13 |
| Study characteristics | 18 | For each study, present characteristics for which data were extracted (e.g., study size, PICOS, follow-up period) and provide the citations. | 5-7,Table 1 |
| Risk of bias within studies | 19 | Present data on risk of bias of each study and, if available, any outcome level assessment. | 10,Figure 2 |
| Results of individual studies | 20 | For all outcomes considered (benefits or harms), present, for each study: 1) simple summary data for each intervention group, and 2) effect estimates and confidence intervals. *Modified approaches may be needed to deal with information from larger networks.* | 10-16 |
| Synthesis of results | 21 | Present results of each meta-analysis done, including confidence/credible intervals. In larger networks, authors may focus on comparisons versus a particular comparator (e.g. placebo or standard care), with full findings presented in an appendix. League tables and forest plots may be considered to summarize pairwise comparisons. If additional summary measures were explored (such as treatment rankings), these should also be presented. | 10-16 |
| Exploration for inconsistency | S5 | Describe results from investigations of inconsistency. This may include such information as measures of model fit to compare consistency and inconsistency models, *P* values from statistical tests, or summary of inconsistency estimates from different parts of the treatment network. | 17 |
| Risk of bias across studies | 22 | Present results of any assessment of risk of bias across studies for the evidence base being studied. | 16,Supplementary Figure S7-12 |
| Results of additional analyses | 23 | Give results of additional analyses, if done (e.g., sensitivity or subgroup analyses, meta-regression analyses*, alternative network geometries studied, alternative choice of prior distributions for Bayesian analyses,* and so forth). | 17 |
|  |  |  |  |
| DISCUSSION |  |  |  |
| Summary of evidence | 24 | Summarize the main findings, including the strength of evidence for each main outcome; consider their relevance to key groups (e.g., healthcare providers, users, and policy-makers). | 17-18 |
| Limitations | 25 | Discuss limitations at study and outcome level (e.g., risk of bias), and at review level (e.g., incomplete retrieval of identified research, reporting bias). *Comment on the validity of the assumptions, such as transitivity and consistency. Comment on any concerns regarding network geometry (e.g., avoidance of certain comparisons).* | 19-20 |
| Conclusions | 26 | Provide a general interpretation of the results in the context of other evidence, and implications for future research. | 21 |
|  |  |  |  |
| FUNDING |  |  |  |
| Funding | 27 | Describe sources of funding for the systematic review and other support (e.g., supply of data); role of funders for the systematic review. This should also include information regarding whether funding has been received from manufacturers of treatments in the network and/or whether some of the authors are content experts with professional conflicts of interest that could affect use of treatments in the network. | 22 |

PICOS = population, intervention, comparators, outcomes, study design.

* Text in italics indicate S wording specific to reporting of network meta-analyses that has been added to guidance from the PRISMA statement.

† Authors may wish to plan for use of appendices to present all relevant information in full detail for items in this section.

| **Table S2.Literature Search Strategy** | |
| --- | --- |
| Pubmed | ("Sports"[MeSH Terms] OR "Sports"[Title/Abstract] OR "Athletics"[Title/Abstract] OR "Athletic"[Title/Abstract] OR ("Resistance Training"[MeSH Terms] OR "Resistance Training"[Title/Abstract] OR "training resistance"[Title/Abstract] OR "strength training"[Title/Abstract] OR "training strength"[Title/Abstract] OR (("Weight-Lifting"[MeSH Terms] OR ("Weight"[All Fields] AND "Lifting"[All Fields]) OR "Weight-Lifting"[All Fields]) AND "strengthening program"[Title/Abstract]) OR ((("strengthen"[All Fields] OR "strengthened"[All Fields] OR "Strengthening"[All Fields] OR "strengthens"[All Fields]) AND ("Program"[All Fields] OR "program s"[All Fields] OR "programe"[All Fields] OR "programed"[All Fields] OR "programes"[All Fields] OR "programing"[All Fields] OR "programmability"[All Fields] OR "programmable"[All Fields] OR "programmably"[All Fields] OR "programme"[All Fields] OR "programme s"[All Fields] OR "programmed"[All Fields] OR "programmer"[All Fields] OR "programmer s"[All Fields] OR "programmers"[All Fields] OR "programmes"[All Fields] OR "programming"[All Fields] OR "programmings"[All Fields] OR "Programs"[All Fields])) AND "Weight-Lifting"[Title/Abstract]) OR ((("strengthen"[All Fields] OR "strengthened"[All Fields] OR "Strengthening"[All Fields] OR "strengthens"[All Fields]) AND ("Program"[All Fields] OR "program s"[All Fields] OR "programe"[All Fields] OR "programed"[All Fields] OR "programes"[All Fields] OR "programing"[All Fields] OR "programmability"[All Fields] OR "programmable"[All Fields] OR "programmably"[All Fields] OR "programme"[All Fields] OR "programme s"[All Fields] OR "programmed"[All Fields] OR "programmer"[All Fields] OR "programmer s"[All Fields] OR "programmers"[All Fields] OR "programmes"[All Fields] OR "programming"[All Fields] OR "programmings"[All Fields] OR "Programs"[All Fields])) AND "Weight-Lifting"[Title/Abstract]) OR (("Weight-Lifting"[MeSH Terms] OR ("Weight"[All Fields] AND "Lifting"[All Fields]) OR "Weight-Lifting"[All Fields]) AND "strengthening program"[Title/Abstract]) OR (("Weight-Lifting"[MeSH Terms] OR ("Weight"[All Fields] AND "Lifting"[All Fields]) OR "Weight-Lifting"[All Fields]) AND "strengthening programs"[Title/Abstract]) OR "weight lifting exercise program"[Title/Abstract] OR ((("Exercise"[MeSH Terms] OR "Exercise"[All Fields] OR "Exercises"[All Fields] OR "exercise therapy"[MeSH Terms] OR ("Exercise"[All Fields] AND "therapy"[All Fields]) OR "exercise therapy"[All Fields] OR "exercising"[All Fields] OR "exercise s"[All Fields] OR "exercised"[All Fields] OR "exerciser"[All Fields] OR "exercisers"[All Fields]) AND ("Program"[All Fields] OR "program s"[All Fields] OR "programe"[All Fields] OR "programed"[All Fields] OR "programes"[All Fields] OR "programing"[All Fields] OR "programmability"[All Fields] OR "programmable"[All Fields] OR "programmably"[All Fields] OR "programme"[All Fields] OR "programme s"[All Fields] OR "programmed"[All Fields] OR "programmer"[All Fields] OR "programmer s"[All Fields] OR "programmers"[All Fields] OR "programmes"[All Fields] OR "programming"[All Fields] OR "programmings"[All Fields] OR "Programs"[All Fields])) AND "Weight-Lifting"[Title/Abstract]) OR ((("Exercise"[MeSH Terms] OR "Exercise"[All Fields] OR "Exercises"[All Fields] OR "exercise therapy"[MeSH Terms] OR ("Exercise"[All Fields] AND "therapy"[All Fields]) OR "exercise therapy"[All Fields] OR "exercising"[All Fields] OR "exercise s"[All Fields] OR "exercised"[All Fields] OR "exerciser"[All Fields] OR "exercisers"[All Fields]) AND ("Program"[All Fields] OR "program s"[All Fields] OR "programe"[All Fields] OR "programed"[All Fields] OR "programes"[All Fields] OR "programing"[All Fields] OR "programmability"[All Fields] OR "programmable"[All Fields] OR "programmably"[All Fields] OR "programme"[All Fields] OR "programme s"[All Fields] OR "programmed"[All Fields] OR "programmer"[All Fields] OR "programmer s"[All Fields] OR "programmers"[All Fields] OR "programmes"[All Fields] OR "programming"[All Fields] OR "programmings"[All Fields] OR "Programs"[All Fields])) AND "Weight-Lifting"[Title/Abstract]) OR "weight lifting exercise program"[Title/Abstract] OR "weight lifting exercise programs"[Title/Abstract] OR "weight bearing strengthening program"[Title/Abstract] OR "strengthening programs weight bearing"[Title/Abstract] OR ((("strengthen"[All Fields] OR "strengthened"[All Fields] OR "Strengthening"[All Fields] OR "strengthens"[All Fields]) AND ("Program"[All Fields] OR "program s"[All Fields] OR "programe"[All Fields] OR "programed"[All Fields] OR "programes"[All Fields] OR "programing"[All Fields] OR "programmability"[All Fields] OR "programmable"[All Fields] OR "programmably"[All Fields] OR "programme"[All Fields] OR "programme s"[All Fields] OR "programmed"[All Fields] OR "programmer"[All Fields] OR "programmer s"[All Fields] OR "programmers"[All Fields] OR "programmes"[All Fields] OR "programming"[All Fields] OR "programmings"[All Fields] OR "Programs"[All Fields])) AND "Weight-Bearing"[Title/Abstract]) OR "weight bearing strengthening program"[Title/Abstract] OR "weight bearing strengthening programs"[Title/Abstract] OR "weight bearing exercise program"[Title/Abstract] OR "exercise programs weight bearing"[Title/Abstract] OR ((("Exercise"[MeSH Terms] OR "Exercise"[All Fields] OR "Exercises"[All Fields] OR "exercise therapy"[MeSH Terms] OR ("Exercise"[All Fields] AND "therapy"[All Fields]) OR "exercise therapy"[All Fields] OR "exercising"[All Fields] OR "exercise s"[All Fields] OR "exercised"[All Fields] OR "exerciser"[All Fields] OR "exercisers"[All Fields]) AND ("Program"[All Fields] OR "program s"[All Fields] OR "programe"[All Fields] OR "programed"[All Fields] OR "programes"[All Fields] OR "programing"[All Fields] OR "programmability"[All Fields] OR "programmable"[All Fields] OR "programmably"[All Fields] OR "programme"[All Fields] OR "programme s"[All Fields] OR "programmed"[All Fields] OR "programmer"[All Fields] OR "programmer s"[All Fields] OR "programmers"[All Fields] OR "programmes"[All Fields] OR "programming"[All Fields] OR "programmings"[All Fields] OR "Programs"[All Fields])) AND "Weight-Bearing"[Title/Abstract]) OR "weight bearing exercise program"[Title/Abstract] OR "weight bearing exercise programs"[Title/Abstract]) OR ("Exercise"[MeSH Terms] OR "Exercise"[Title/Abstract] OR "Exercises"[Title/Abstract] OR "exercise physical"[Title/Abstract] OR "exercises physical"[Title/Abstract] OR "physical exercise"[Title/Abstract] OR "physical exercises"[Title/Abstract] OR "exercise aerobic"[Title/Abstract] OR "aerobic exercise"[Title/Abstract] OR "aerobic exercises"[Title/Abstract] OR "exercises aerobic"[Title/Abstract] OR "exercise isometric"[Title/Abstract] OR "exercises isometric"[Title/Abstract] OR "isometric exercises"[Title/Abstract] OR "isometric exercise"[Title/Abstract] OR "acute exercise"[Title/Abstract] OR "acute exercises"[Title/Abstract] OR "exercise acute"[Title/Abstract] OR "exercises acute"[Title/Abstract] OR "exercise training"[Title/Abstract] OR "exercise trainings"[Title/Abstract] OR "training exercise"[Title/Abstract] OR (("education"[MeSH Subheading] OR "education"[All Fields] OR "Training"[All Fields] OR "education"[MeSH Terms] OR "train"[All Fields] OR "train s"[All Fields] OR "trained"[All Fields] OR "training s"[All Fields] OR "Trainings"[All Fields] OR "trains"[All Fields]) AND "Exercise"[Title/Abstract]) OR "physical activity"[Title/Abstract] OR "activities physical"[Title/Abstract] OR "activity physical"[Title/Abstract] OR "physical activities"[Title/Abstract]) OR ("Proteins"[MeSH Terms] OR "Proteins"[Title/Abstract] OR "Protein"[Title/Abstract] OR "gene products protein"[Title/Abstract] OR "protein gene products"[Title/Abstract] OR "gene proteins"[Title/Abstract] OR "proteins gene"[Title/Abstract])) AND ((((("Sarcopenia"[Mesh]) OR (Sarcopenias[Title/Abstract])) OR (Sarcopenia[Title/Abstract])) AND (("Obesity"[Mesh]) OR (Obesity[Title/Abstract]))) AND ((("Aged"[Mesh]) OR (Elderly[Title/Abstract])) OR (Aged[Title/Abstract]))) |
|  |  |
|  |  |
|  |  |
|  |  |
|  |  |
|  |  |
| Web of Science | 1: ((TS=(Sarcopenia or Sarcopenias)) AND TS=(Obesity)) AND TS=(Aged or Elderly)  2: TS=("Resistance Training" OR "Training, Resistance" OR "Strength Training" OR "Training, Strength" OR "Weight-Lifting Strengthening Program" OR "Strengthening Programs, Weight-Lifting" OR "Strengthening Program, Weight-Lifting" OR "Weight Lifting Strengthening Program" OR "Weight-Lifting Strengthening Programs" OR "Weight-Lifting Exercise Program" OR "Exercise Programs, Weight-Lifting" OR "Exercise Program, Weight-Lifting" OR "Weight Lifting Exercise Program" OR "Weight-Lifting Exercise Programs" OR "Weight-Bearing Strengthening Program" OR "Strengthening Programs, Weight-Bearing" OR "Strengthening Program, Weight-Bearing" OR "Weight Bearing Strengthening Program" OR "Weight-Bearing Strengthening Programs" OR "Weight-Bearing Exercise Program" OR "Exercise Programs, Weight-Bearing" OR "Exercise Program, Weight-Bearing" OR "Weight Bearing Exercise Program" OR "Weight-Bearing Exercise Programs")  3: TS=("Resistance Training" OR "Training, Resistance" OR "Strength Training" OR "Training, Strength" OR "Weight-Lifting Strengthening Program" OR "Strengthening Programs, Weight-Lifting" OR "Strengthening Program, Weight-Lifting" OR "Weight Lifting Strengthening Program" OR "Weight-Lifting Strengthening Programs" OR "Weight-Lifting Exercise Program" OR "Exercise Programs, Weight-Lifting" OR "Exercise Program, Weight-Lifting" OR "Weight Lifting Exercise Program" OR "Weight-Lifting Exercise Programs" OR "Weight-Bearing Strengthening Program" OR "Strengthening Programs, Weight-Bearing" OR "Strengthening Program, Weight-Bearing" OR "Weight Bearing Strengthening Program" OR "Weight-Bearing Strengthening Programs" OR "Weight-Bearing Exercise Program" OR "Exercise Programs, Weight-Bearing" OR "Exercise Program, Weight-Bearing" OR "Weight Bearing Exercise Program" OR "Weight-Bearing Exercise Programs")  4: (TS=("Resistance Training" OR "Training, Resistance" OR "Strength Training" OR "Training, Strength" OR "Weight-Lifting Strengthening Program" OR "Strengthening Programs, Weight-Lifting" OR "Strengthening Program, Weight-Lifting" OR "Weight Lifting Strengthening Program" OR "Weight-Lifting Strengthening Programs" OR "Weight-Lifting Exercise Program" OR "Exercise Programs, Weight-Lifting" OR "Exercise Program, Weight-Lifting" OR "Weight Lifting Exercise Program" OR "Weight-Lifting Exercise Programs" OR "Weight-Bearing Strengthening Program" OR "Strengthening Programs, Weight-Bearing" OR "Strengthening Program, Weight-Bearing" OR "Weight Bearing Strengthening Program" OR "Weight-Bearing Strengthening Programs" OR "Weight-Bearing Exercise Program" OR "Exercise Programs, Weight-Bearing" OR "Exercise Program, Weight-Bearing" OR "Weight Bearing Exercise Program" OR "Weight-Bearing Exercise Programs")) OR TS=(Proteins OR Protein OR "Gene Products, Protein" OR "Protein Gene Products" OR Gene Proteins OR "Proteins, Gene")  5: TS=(sports or Athletics or Athletic)  6: TS=("Exercise" OR "Exercises" OR "Exercise, Physical" OR "Exercises, Physical" OR "Physical Exercise" OR "Physical Exercises" OR "Exercise, Aerobic" OR "Aerobic Exercise" OR "Aerobic Exercises" OR "Exercises, Aerobic" OR "Exercise, Isometric" OR "Exercises, Isometric" OR "Isometric Exercises" OR "Isometric Exercise" OR "Acute Exercise" OR "Acute Exercises" OR "Exercise, Acute" OR "Exercises, Acute" OR "Exercise Training" OR "Exercise Trainings" OR "Training, Exercise" OR "Trainings, Exercise" OR "Physical Activity" OR "Activities, Physical" OR "Activity, Physical" OR "Physical Activities")  7: #2 OR #4 OR #5 OR #6  8: #2 OR #4 OR #5 OR #6  9: #8 AND #1  10: #8 AND #1  11: #6 OR #5 OR #4 OR #3  12: #6 OR #5 OR #4 OR #3  13: TS=( random* OR randomi* OR randomly OR placebo* OR sham OR trial OR "clinical trial" OR "controlled clinical trial" OR "double blind" OR "single blind" OR "triple blind" OR crossover OR "cross over" OR "parallel group" OR cluster random* OR RCT)  14: #13 AND #9  15: TS=( random* OR randomi* OR randomly OR placebo* OR sham OR trial OR "clinical trial" OR "controlled clinical trial" OR "double blind" OR "single blind" OR "triple blind" OR crossover OR "cross over" OR "parallel group" OR cluster random* OR RCT)  16: #13 AND #9 |
|  |  |
|  |  |
|  |  |
|  |  |
|  |  |
|  |  |
|  |  |
| Cochrane | ID Search Hits  #1 MeSH descriptor: [Sarcopenia] explode all trees  #2 (sarcopenia):ti,ab,kw OR (saicopenias):ti,ab,kw  #3 #1or#2 3181  #4 MeSH descriptor: [Obesity] explode all trees  #5 MeSH descriptor: [Aged] explode all trees  #6 (aged):ti,ab,kw OR (Elderly):ti,ab,kw  #7 #5or#6  #8 #3and#4and#7  #9 MeSH descriptor: [Sports] explode all trees  #10 (sports):ti,ab,kw OR (Sport):ti,ab,kw OR (Athletic):ti,ab,kw OR (Athletics):ti,ab,kw  #11 #9or#10  #12 MeSH descriptor: [Exercise] explode all trees  #13 (Activities or Physical or Physical Activities or Activity, Physical or Physical Activity or Exercises, Isometric or Exercise, Isometric or Isometric Exercises or Isometric Exercise or Exercise Trainings or Exercise Training or Trainings, Exercise or Training, Exercise or Exercise, Aerobic or Aerobic Exercise or Aerobic Exercises or Exercises, Aerobic or Acute Exercises or Acute Exercise or Exercise, Acute or Exercises, Acute or Physical Exercises or Exercise, Physical or Exercises, Physical or Exercises or Physical Exercise):ti,ab,kw  #14 #12or#13  #15 MeSH descriptor: [Resistance Training] explode all trees  #16 Training, Resistance or Strength Training or Training, Strength or Weight-Bearing Exercise Program or Weight Bearing Exercise Program or Weight-Bearing Strengthening Program or Weight-Bearing Exercise Programs or Exercise Program, Weight-Bearing or Strengthening Program, Weight-Bearing or Strengthening Programs, Weight-Bearing or Weight Bearing Strengthening Program or Exercise Programs, Weight-Bearing or Weight-Bearing Strengthening Programs or Weight-Lifting Exercise Programs or Weight Lifting Exercise Program or Exercise Program, Weight-Lifting or Weight-Lifting Exercise Program or Weight Lifting Strengthening Program or Weight-Lifting Strengthening Program; Strengthening Programs, Weight-Lifting; Weight-Lifting Strengthening Programs; Exercise Programs, Weight-Lifting or Strengthening Program, Weight-Lifting  #17 #15or#16  #18 MeSH descriptor: [Proteins] explode all trees  #19 Gene Products, Protein or Protein Gene Products or Gene Proteins or Proteins, Gene  #20 #18or#19  #21 #11or#14or#17or#20  #22 #8and# |
|  |  |
|  |  |
|  |  |
| Embase | 'sarcopenia'/exp OR 'age-related muscle atrophy':ti,ab,kw OR 'age-related muscle decline':ti,ab,kw OR 'age-related muscular decline':ti,ab,kw OR 'age-related muscular degeneration':ti,ab,kw OR 'ageing-related muscle atrophy':ti,ab,kw OR 'geriatric muscular atrophy':ti,ab,kw OR 'sarcopenia':ti,ab,kw AND 'obesity'/exp OR 'adipose tissue hyperplasia':ti,ab,kw OR 'adipositas':ti,ab,kw OR 'adiposity':ti,ab,kw OR 'alimentary obesity':ti,ab,kw OR 'body weight, excess':ti,ab,kw OR 'corpulency':ti,ab,kw OR 'fat overload syndrome':ti,ab,kw OR 'nutritional obesity':ti,ab,kw OR 'obesitas':ti,ab,kw OR 'overweight':ti,ab,kw OR 'obesity':ti,ab,kw AND 'aged'/exp OR 'aged patient':ti,ab,kw OR 'aged people':ti,ab,kw OR 'aged person':ti,ab,kw OR 'aged subject':ti,ab,kw OR 'elderly':ti,ab,kw OR 'elderly patient':ti,ab,kw OR 'elderly people':ti,ab,kw OR 'elderly person':ti,ab,kw OR 'elderly subject':ti,ab,kw OR 'senior citizen':ti,ab,kw OR 'senium':ti,ab,kw OR 'aged':ti,ab,kw AND 'sport'/exp OR 'competitive gymnastics':ti,ab,kw OR 'competitive sport':ti,ab,kw OR 'sports':ti,ab,kw OR 'sport':ti,ab,kw OR 'aerobic exercise'/exp OR 'aerobic dance':ti,ab,kw OR 'aerobic dancing':ti,ab,kw OR 'aerobics':ti,ab,kw OR 'aerobics exercise':ti,ab,kw OR 'dancing, aerobic':ti,ab,kw OR 'exercise, aerobic':ti,ab,kw OR 'low impact aerobic exercise':ti,ab,kw OR 'low impact aerobics':ti,ab,kw OR 'step aerobics':ti,ab,kw OR 'aerobic exercise':ti,ab,kw OR 'resistance training'/exp OR 'resistance exercise':ti,ab,kw OR 'resistance exercise training':ti,ab,kw OR 'resistance-type exercise':ti,ab,kw OR 'resistance-type training':ti,ab,kw OR 'strength training':ti,ab,kw OR 'strength-type exercise':ti,ab,kw OR 'strength-type training':ti,ab,kw OR 'resistance training':ti,ab,kw OR 'protein'/exp OR 'alpha protein':ti,ab,kw OR 'cationic protein':ti,ab,kw OR 'delta protein':ti,ab,kw OR 'endogenous protein':ti,ab,kw OR 'kationic protein':ti,ab,kw OR 'protein accumulation':ti,ab,kw OR 'protein particle':ti,ab,kw OR 'proteins':ti,ab,kw OR 'soluble protein':ti,ab,kw OR 'specific protein':ti,ab,kw OR 'total protein':ti,ab,kw OR 'protein':ti,ab,kw AND 'randomized controlled trial'/de |
|  |  |
|  |  |
|  |  |
|  |  |
|  |  |
|  |  |
|  |  |
|  |  |
|  |  |
|  |  |

Supplementary Table3. Global inconsistency table for BMI, GRIP, FM, PBF, and SMI outcomes in older adults with sarcopenic obesity

| **结局指标名称** | **全局不一致性分析的P值** |
| --- | --- |
| BMI | 0.2779 |
| FM | 0.8674 |
| GRIP | 0.8533 |
| PBF | 0.0643 |
| SMI | 0.2026 |

Supplementary Table4. Node-splitting analysis for the BMI outcome in older adults with sarcopenic obesity

| **Side** | **Coef.** | **Std. Err.** | **Coef.** | **Std. Err.** | **Coef.** | **Std. Err.** | **P>z** | **tau** | |
| --- | --- | --- | --- | --- | --- | --- | --- | --- | --- |
| A E | 0.0029839 | 1.51065 | 0.2637163 | 2.503627 | -0.2607324 | 2.999666 | 0.931 | | 1.822385 |
| A F | -0.1 | 1.249247 | -0.4891579 | 11.80956 | 0.3891579 | 11.913 | 0.974 | | 1.730662 |
| A H | 0.3997216 | 1.476285 | 0.0676009 | 2.542121 | 0.3321207 | 2.986422 | 0.911 | | 1.801376 |
| B H | -0.4999999 | 0.4814128 | 0.6320426 | 145.2419 | -1.132043 | 145.2424 | 0.994 | | 1.719036 |
| C E | -1.514761 | 1.22237 | 1.185594 | 0.4087467 | -2.700355 | 1.273371 | 0.034 | | 1.46e-07 |
| C H | 1.161399 | 0.3929784 | -2.696638 | 1.831657 | 3.858037 | 1.830708 | 0.035 | | 2.26e-06 |
| D H | -0.1921322 | 0.7164984 | 0.6912459 | 109.5983 | -0.883378 | 109.6025 | 0.994 | | 1.719105 |
| E F | -0.1 | 1.355516 | -0.4217112 | 2.643899 | 0.3217112 | 3.204313 | 0.92 | | 1.828207 |
| E G | -0.7 | 1.474812 | 0.903139 | 2.601725 | -1.603139 | 2.890769 | 0.579 | | 2.425302 |
| E H | 0.1942852 | 0.381315 | 2.864439 | 2.322898 | -2.670154 | 2.310107 | 0.248 | | 1.452749 |
| F H | 0.5 | 1.316864 | 0.1511566 | 2.695191 | 0.3488434 | 3.198779 | 0.913 | | 1.820158 |
| G H | 0.2 | 1.491583 | 1.803156 | 2.572889 | -1.603156 | 2.890774 | 0.579 | | 2.42529 |

Supplementary Table5. Node-splitting analysis for the GRIP outcome in older adults with sarcopenic obesity

|  | **Coef.** | **Std. Err.** | **Coef.** | **Std. Err.** | **Coef.** | **Std. Err.** | **P>z** | **tau** |
| --- | --- | --- | --- | --- | --- | --- | --- | --- |
| A F | 5.901878 | 3.135696 | -1.821557 | 5.565814 | 7.723434 | 6.307297 | 0.221 | 1.718189 |
| A G | 0.4986698 | 3.175272 | 9.740996 | 6.08391 | -9.242326 | 6.835995 | 0.176 | 1.691465 |
| A H | 0.2073443 | 3.930934 | 0.0480895 | 5.376856 | 0.1592547 | 7.338312 | 0.983 | 1.787102 |
| B H | -1.8 | 2.005051 | 0.3344535 | 318.9434 | -2.134453 | 318.9492 | 0.995 | 1.745324 |
| C D | 0.6 | 1.958101 | -4.943198 | 3.75911 | 5.543198 | 4.157883 | 0.182 | 1.63416 |
| C E | 0.7568746 | 2.019596 | -2.53918 | 2.18939 | 3.296055 | 2.958926 | 0.265 | 1.702762 |
| C F | 0.1989159 | 1.919462 | 2.834173 | 1.49111 | -2.635257 | 2.431394 | 0.278 | 1.687172 |
| C H | -1.835668 | 1.050292 | -3.973252 | 2.646072 | 2.137584 | 2.85778 | 0.454 | 1.776585 |
| D E | 0.1 | 2.237562 | -2.129379 | 5.393045 | 2.229379 | 5.842624 | 0.703 | 1.86605 |
| D H | -0.7 | 2.102527 | -4.380247 | 3.623342 | 3.680247 | 4.140254 | 0.374 | 1.766962 |
| E H | -0.7846691 | 1.397109 | -6.326234 | 3.943832 | 5.541565 | 4.157478 | 0.183 | 1.634109 |
| F G | -5.391743 | 3.097666 | 0.0030007 | 2.063423 | -5.394744 | 3.717767 | 0.147 | 1.704412 |
| F H | -3.952795 | 1.02029 | -3.799603 | 2.846315 | -0.1531921 | 3.050319 | 0.96 | 1.835975 |
| G H | -3.057871 | 1.699873 | 4.739903 | 5.984392 | -7.797774 | 6.362966 | 0.22 | 1.718833 |

Supplementary Table6. Node-splitting analysis for the FM outcome in older adults with sarcopenic obesity

|  | **Coef.** | **Std.Err.** | **Coef.** | **Std.Err.** | **Coef.** | **Std. Err.** | **P>z** | **tau** |
| --- | --- | --- | --- | --- | --- | --- | --- | --- |
| A F | 0.5000915 | 2.366447 | -1.434079 | 4.695307 | 1.934171 | 5.491359 | 0.725 | 1.38E-07 |
| A G | -4.83E-09 | 2.119119 | -3.287339 | 103.221 | 3.287339 | 103.2413 | 0.975 | 1.47E-06 |
| A I | 1.901729 | 2.415401 | 3.808004 | 4.620249 | -1.906275 | 5.491366 | 0.728 | 1.96E-06 |
| B I | 0.2082886 | 0.7400869 | 4.717344 | 227.643 | -4.509055 | 227.6443 | 0.984 | 3.30E-08 |
| C D | . | . | . | . | . | . | . | . |
| C E | 0.2 | 1.06998 | 1.335419 | 3.111163 | -1.135419 | 3.301717 | 0.731 | 0.0001892 |
| C I | 0.1 | 1.103962 | -1.034703 | 3.075224 | 1.134703 | 3.30161 | 0.731 | 0.00002 |
| D E | 0.3 | 1.109826 | 1.43542 | 3.125091 | -1.13542 | 3.301717 | 0.731 | 0.0001931 |
| D I | 0.2 | 1.142623 | -0.9347032 | 3.089314 | 1.134703 | 3.30161 | 0.731 | 0.0000197 |
| E I | -0.3619353 | 0.8229678 | 4.814511 | 198.3157 | -5.176446 | 198.3181 | 0.979 | 1.61E-08 |
| F G | -0.5 | 2.399028 | 1.435824 | 4.727722 | -1.935824 | 5.506967 | 0.725 | 8.24E-09 |
| F H | -0.7843493 | 1.919738 | 5.515883 | 6.130403 | -6.300232 | 6.386473 | 0.324 | 1.46E-08 |
| F I | 2.32512 | 0.6781354 | -0.331668 | 7.74738 | 2.656788 | 7.779392 | 0.733 | 1.78E-07 |
| G I | 1.9 | 2.447379 | 3.826474 | 4.652778 | -1.926474 | 5.506966 | 0.726 | 6.93E-06 |
| H I | 0.0893589 | 3.54884 | 3.538025 | 2.258139 | -3.448666 | 4.1718 | 0.408 | 6.45E-06 |

Supplementary Table7.Node-splitting analysis for the PBF outcome in older adults with sarcopenic obesity

|  | **Coef.** | **Std. Err.** | **Coef.** | **Std. Err.** | **Coef.** | **Std. Err.** | **P>z** | **tau** |
| --- | --- | --- | --- | --- | --- | --- | --- | --- |
| A F | 0.5069536 | 3.183218 | -0.2831202 | 4.717696 | 0.7900738 | 5.817261 | 0.892 | 2.510212 |
| A G | 0.0091072 | 3.153177 | 0.1229134 | 6.423231 | -0.1138062 | 7.222751 | 0.987 | 2.521805 |
| A I | 2.607296 | 3.135143 | 2.464954 | 4.610046 | 0.1423424 | 5.661584 | 0.98 | 2.511415 |
| B I | 1.2 | 3.418249 | 5.123176 | 320.1883 | -3.923176 | 320.2036 | 0.99 | 2.439669 |
| C D | -0.1000001 | 2.521873 | 8.548556 | 4.645625 | -8.648557 | 5.281586 | 0.102 | 2.291601 |
| C E | 0.2278305 | 2.524536 | 5.497534 | 2.560259 | -5.269703 | 3.605265 | 0.144 | 2.345437 |
| C F | -1.659986 | 1.558655 | 3.687773 | 1.463261 | -5.34776 | 2.133006 | 0.012 | 2.048251 |
| C I | 5.04049 | 1.104608 | -0.5862236 | 1.713011 | 5.626714 | 2.029841 | 0.006 | 1.911019 |
| D E | 0.3 | 2.683167 | 4.957554 | 6.519016 | -4.657554 | 7.016237 | 0.507 | 2.51046 |
| D I | 0.3 | 2.721212 | 5.412548 | 4.573399 | -5.112548 | 5.354153 | 0.34 | 2.469886 |
| E I | -0.2932196 | 1.71783 | 8.353584 | 4.954187 | -8.646803 | 5.279467 | 0.101 | 2.291513 |
| F G | -0.4989549 | 3.303782 | -0.0342154 | 2.693552 | -0.4647394 | 4.272839 | 0.913 | 2.529676 |
| F H | -1.1 | 2.987112 | -0.5190944 | 277.4287 | -0.5809057 | 277.4461 | 0.998 | 2.439686 |
| F I | 1.752479 | 0.8725556 | 5.652747 | 2.185189 | -3.900269 | 2.348764 | 0.097 | 2.264118 |
| G I | 2.483312 | 2.09681 | 2.898237 | 5.901028 | -0.4149249 | 6.258314 | 0.947 | 2.520137 |

Supplementary Table8. Node-splitting analysis for the SMI outcome in older adults with sarcopenic obesity

|  | **Coef.** | **Std. Err.** | **Coef.** | **Std. Err.** | **Coef.** | **Std. Err.** | **P>z** | **tau** |
| --- | --- | --- | --- | --- | --- | --- | --- | --- |
| A B | 1 | 0.8602325 | -2.091008 | 2.159437 | 3.091008 | 2.425525 | 0.203 | 1.83e-09 |
| A C | 0.5 | 0.9219523 | -2.591008 | 2.184758 | 3.091008 | 2.425525 | 0.203 | 6.99e-09 |
| A D | -5.96E-11 | 0.2599012 | 1.544864 | 1.184327 | -1.544864 | 1.212509 | 0.203 | 1.49e-09 |
| A E | 1.1 | 1.063015 | -0.4455038 | 0.5837744 | 1.545504 | 1.212762 | 0.203 | 1.48e-09 |
| B C | . | . | . | . | . | . | . |  |
| B E | 0.1 | 0.9433982 | -2.991008 | 2.052601 | 3.091008 | 2.425525 | 0.203 | 2.58e-11 |
| C E | 0.6 | 0.9999981 | -2.491008 | 2.079223 | 3.091008 | 2.425525 | 0.203 | 1.42e-09 |
| D E | -0.4455159 | 0.5227405 | 1.099886 | 1.094276 | -1.545401 | 1.212721 | 0.203 | 1.49e-09 |


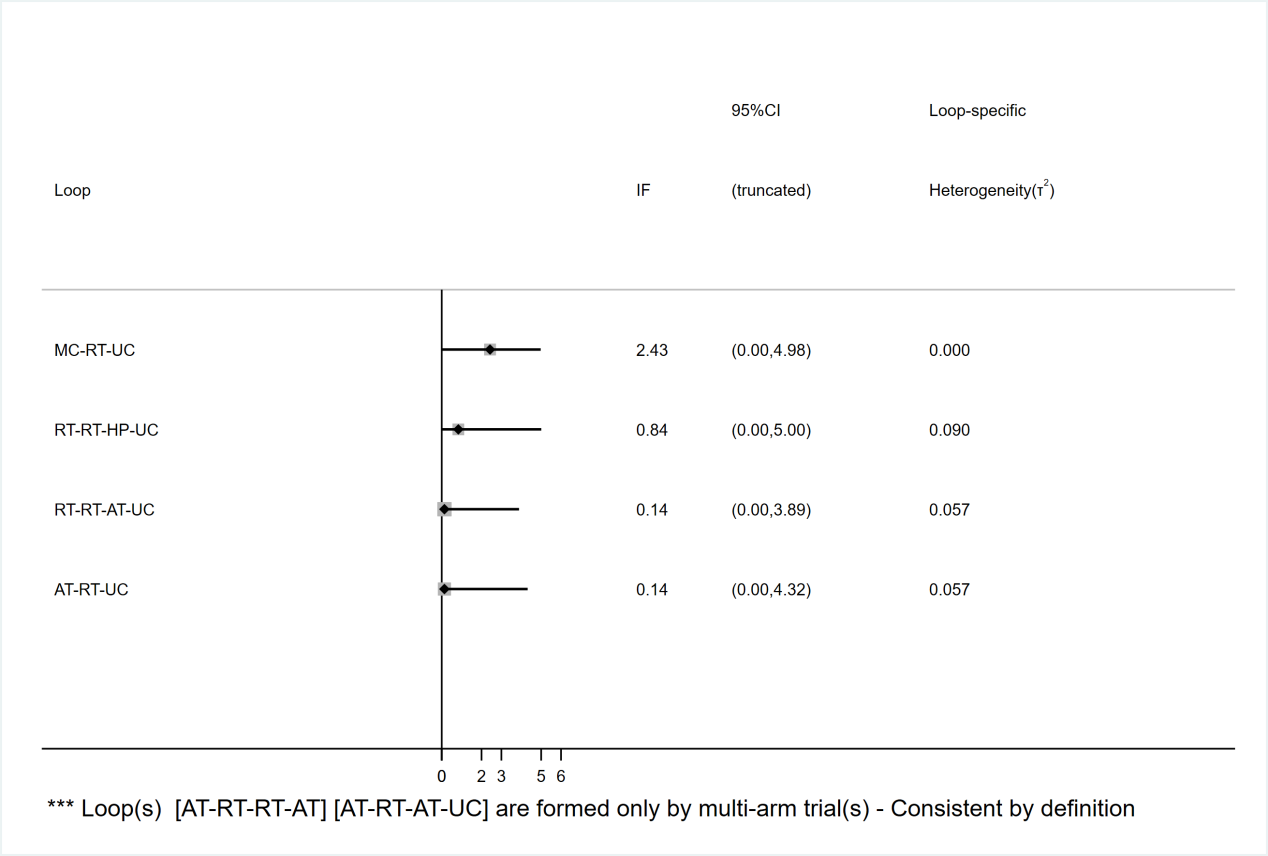


Supplementary Figure1. Loop inconsistency plot for the BMI outcome in older adults with sarcopenic obesity
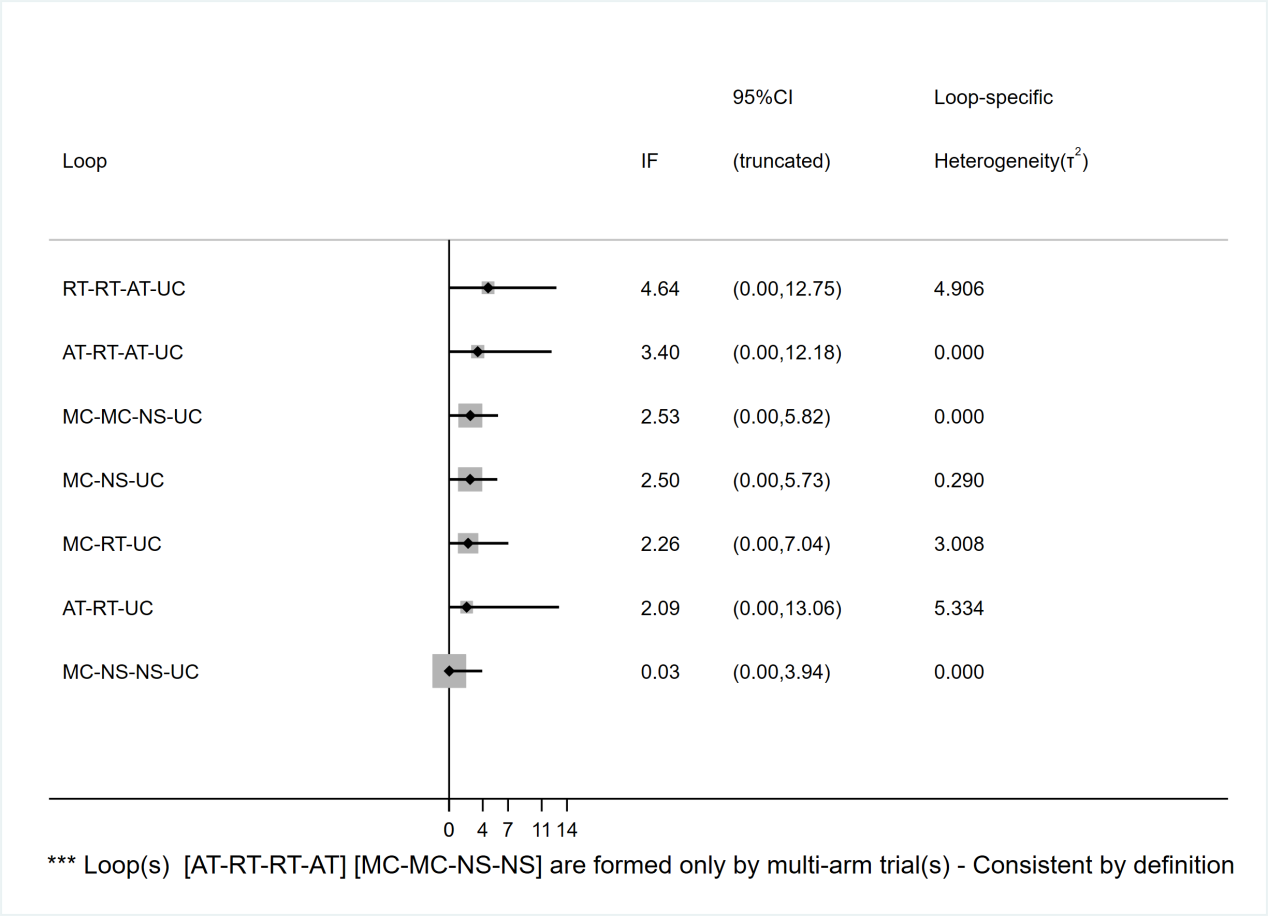
Supplementary Figure2. Loop inconsistency plot for the GRIP outcome in older adults with sarcopenic obesity


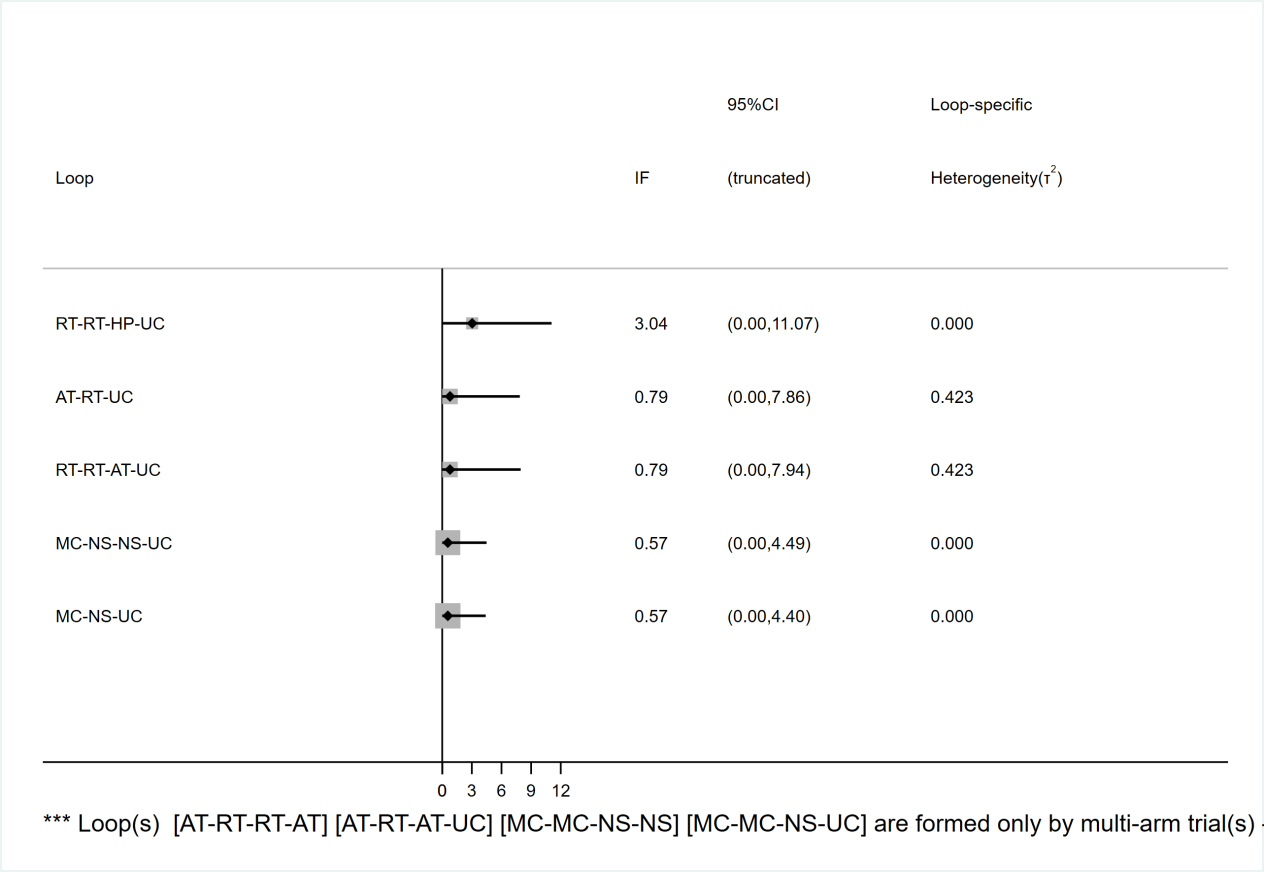


Supplementary Figure3. Loop inconsistency plot for the FM outcome in older adults with sarcopenic obesity


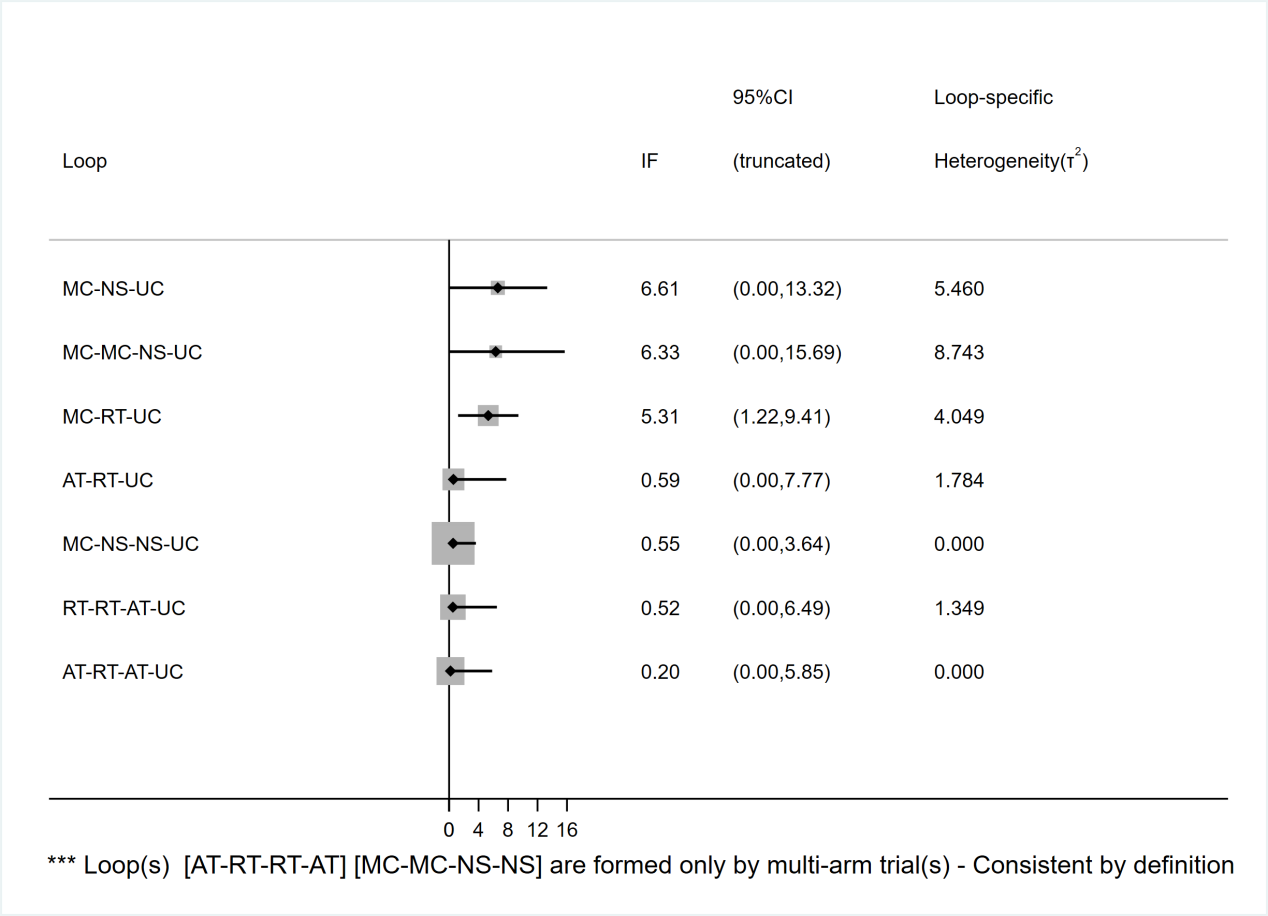


Supplementary Figure4. Loop inconsistency plot for the PBF outcome in older adults with sarcopenic obesity


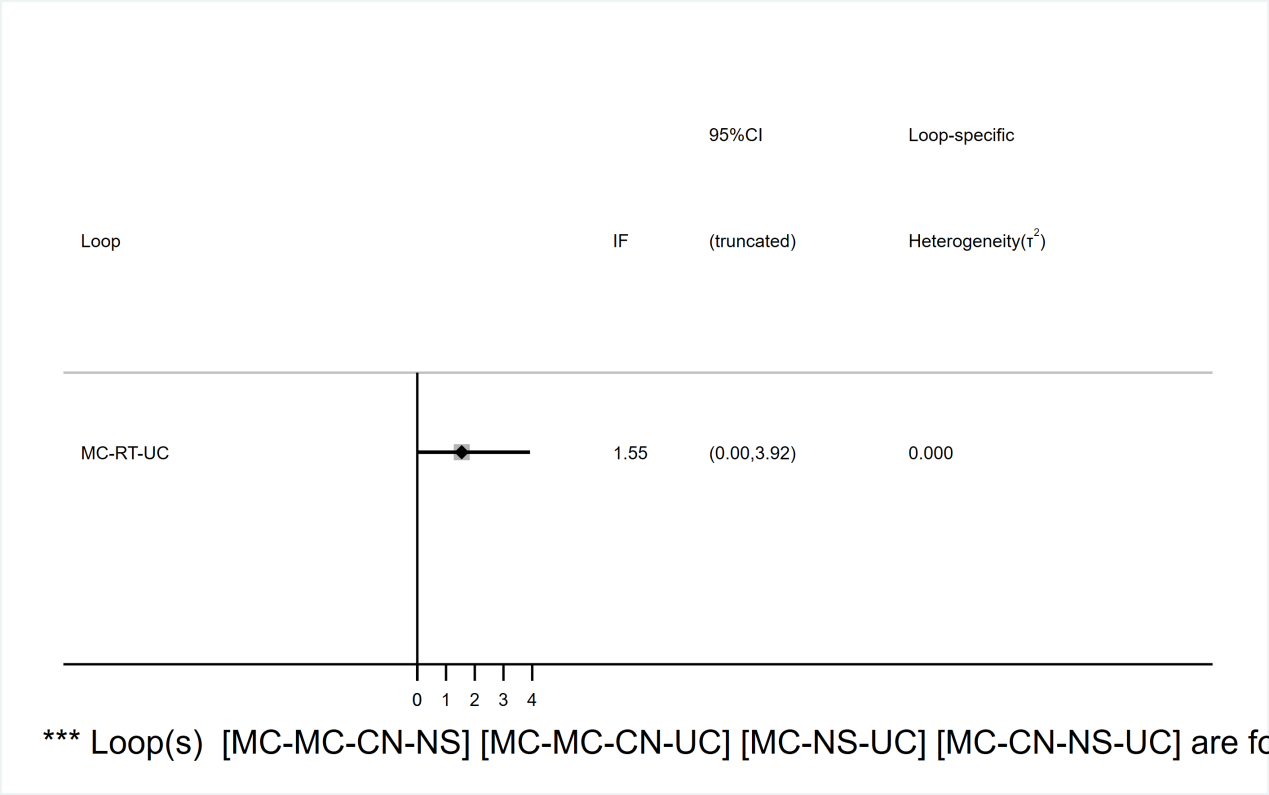


Supplementary Figure5. Loop inconsistency plot for the SMI outcome in older adults with sarcopenic obesity

Supplementary Table9. SUCRA ranking table for the BMI outcome in older adults with sarcopenic obesity

| **Treatm~t** | **SUCRA** | **PrBest** | **MeanRank** |
| --- | --- | --- | --- |
| MC | 85.1 | 36.6 | 2 |
| RT-HP | 59.3 | 26.7 | 3.8 |
| RT-AT | 56.3 | 16 | 4.1 |
| AT | 52.6 | 17.3 | 4.3 |
| RT | 52.2 | 1 | 4.3 |
| UC | 40.6 | 0 | 5.2 |
| NS | 34.3 | 2.4 | 5.6 |
| HP | 19.6 | 0.1 | 6.6 |

Supplementary Table10. SUCRA ranking table for the GRIP outcome in older adults with sarcopenic obesity

| **Treatm~t** | **SUCRA** | **PrBest** | **MeanRank** |
| --- | --- | --- | --- |
| RT | 90.9 | 56.2 | 1.6 |
| RT-AT | 62.1 | 13.2 | 3.7 |
| MC | 59.3 | 2.8 | 3.8 |
| HP | 51.5 | 12.4 | 4.4 |
| MC-NS | 48.7 | 7.7 | 4.6 |
| NS | 44.2 | 2.8 | 4.9 |
| AT | 26.7 | 4.9 | 6.1 |
| UC | 16.4 | 0 | 6.9 |

Supplementary Table11.SUCRA ranking table for the FM outcome in older adults with sarcopenic obesity

| **Treatm~t** | **SUCRA** | **PrBest** | **MeanRank** |
| --- | --- | --- | --- |
| RT | 79 | 13.5 | 2.7 |
| RT-HP | 75.3 | 33.8 | 3 |
| AT | 72.5 | 25.5 | 3.2 |
| RT-AT | 72.4 | 26 | 3.2 |
| HP | 35.9 | 0.3 | 6.1 |
| MC-NS | 34.2 | 0.5 | 6.3 |
| MC | 30.7 | 0.4 | 6.5 |
| UC | 29.5 | 0 | 6.6 |
| NS | 20.5 | 0 | 7.4 |

Supplementary Table12. SUCRA ranking table for the PBF outcome in older adults with sarcopenic obesity

| **Treatm~t** | **SUCRA** | **PrBest** | **MeanRank** |
| --- | --- | --- | --- |
| MC | 77.1 | 17.9 | 2.8 |
| RT-HP | 66.3 | 32.4 | 3.7 |
| RT-AT | 58.6 | 10.8 | 4.3 |
| AT | 57.2 | 16 | 4.4 |
| RT | 55.8 | 0.9 | 4.5 |
| MC-NS | 46.9 | 8.2 | 5.2 |
| HP | 41.4 | 12.8 | 5.7 |
| NS | 30.2 | 1.1 | 6.6 |
| UC | 16.5 | 0 | 7.7 |

Supplementary Table13.SUCRA ranking table for the SMI outcome in older adults with sarcopenic obesity

| **Treatm~t** | **SUCRA** | **PrBest** | **MeanRank** |
| --- | --- | --- | --- |
| MC-CN | 74.1 | 52.5 | 2 |
| RT | 51.4 | 12.8 | 2.9 |
| NS | 44.1 | 17.7 | 3.2 |
| MC | 42.5 | 7.7 | 3.3 |
| UC | 37.8 | 9.4 | 3.5 |

Supplementary Table14.Sensitivity analysis for the BMI outcome in older adults with sarcopenic obesity

| **dropped_id** | **comparison** | **eff** | **lci** | **uci** | **connected** |
| --- | --- | --- | --- | --- | --- |
| M Aubertin-Leheudre | ATvsUC | -0.24909608 | -0.9667971 | 0.468605 | 1 |
| M Aubertin-Leheudre | HPvsUC | 0.21454728 | -0.2857278 | 0.7148223 | 1 |
| M Aubertin-Leheudre | MCvsUC | -0.43115693 | -0.7973402 | -0.0649737 | 1 |
| M Aubertin-Leheudre | NSvsUC | 0.07436016 | -0.4108098 | 0.5595301 | 1 |
| M Aubertin-Leheudre | RTvsUC | -0.34919096 | -0.629051 | -0.0693309 | 1 |
| M Aubertin-Leheudre | RT-ATvsUC | -0.27590246 | -0.993699 | 0.4418941 | 1 |
| M Aubertin-Leheudre | RT-HPvsUC | -0.2938689 | -1.180767 | 0.5930293 | 1 |
| Ebrahim Banitalebi | ATvsUC | -0.26303764 | -0.9887674 | 0.4626922 | 1 |
| Ebrahim Banitalebi | HPvsUC | 0.21454728 | -0.2882045 | 0.7172991 | 1 |
| Ebrahim Banitalebi | MCvsUC | -0.43389907 | -0.8017638 | -0.0660343 | 1 |
| Ebrahim Banitalebi | NSvsUC | 0.0607765 | -0.3803981 | 0.5019511 | 1 |
| Ebrahim Banitalebi | RTvsUC | -0.37308968 | -0.6959711 | -0.0502082 | 1 |
| Ebrahim Banitalebi | RT-ATvsUC | -0.28984749 | -1.015676 | 0.4359811 | 1 |
| Ebrahim Banitalebi | RT-HPvsUC | -0.30465118 | -1.195271 | 0.5859686 | 1 |
| Hung-Ting Chen | ATvsUC | 0.21454728 | -0.2633654 | 0.6924599 | 1 |
| Hung-Ting Chen | HPvsUC | -0.43598495 | -0.7947816 | -0.0771884 | 1 |
| Hung-Ting Chen | MCvsUC | 0.06169361 | -0.3594126 | 0.4827999 | 1 |
| Hung-Ting Chen | NSvsUC | -0.37991316 | -0.6662278 | -0.0935985 | 1 |
| Hung-Ting Chen | RTvsUC | -0.30698964 | -1.184871 | 0.5708922 | 1 |
| shih-Wei huanG | ATvsUC | -0.2928378 | -0.9813753 | 0.3956997 | 1 |
| shih-Wei huanG | HPvsUC | 0.21454728 | -0.2250103 | 0.6541049 | 1 |
| shih-Wei huanG | MCvsUC | -0.44262616 | -0.7889976 | -0.0962547 | 1 |
| shih-Wei huanG | NSvsUC | 0.06315187 | -0.3262509 | 0.4525547 | 1 |
| shih-Wei huanG | RTvsUC | -0.41991631 | -0.7036926 | -0.1361401 | 1 |
| shih-Wei huanG | RT-ATvsUC | -0.31965876 | -1.008297 | 0.3689797 | 1 |
| shih-Wei huanG | RT-HPvsUC | -0.32367993 | -1.185978 | 0.5386183 | 1 |
| Mathieu L | ATvsUC | -0.28028152 | -0.9739397 | 0.4133766 | 1 |
| Mathieu L | HPvsUC | 0.21454728 | -0.2399372 | 0.6690318 | 1 |
| Mathieu L | MCvsUC | -0.43951031 | -0.7903528 | -0.0886679 | 1 |
| Mathieu L | NSvsUC | 0.06257887 | -0.3392591 | 0.4644168 | 1 |
| Mathieu L | RTvsUC | -0.39965318 | -0.671742 | -0.1275644 | 1 |
| Mathieu L | RT-ATvsUC | -0.30709826 | -1.000855 | 0.3866583 | 1 |
| espedita Muscariello | ATvsUC | -0.254025 | -0.9582018 | 0.4501518 | 1 |
| espedita Muscariello | HPvsUC | -0.43330679 | -0.7911375 | -0.0754761 | 1 |
| espedita Muscariello | MCvsUC | 0.06177637 | -0.3574986 | 0.4810514 | 1 |
| espedita Muscariello | NSvsUC | -0.3563651 | -0.6294527 | -0.0832775 | 1 |
| espedita Muscariello | RTvsUC | -0.28083371 | -0.9851072 | 0.4234398 | 1 |
| espedita Muscariello | RT-ATvsUC | -0.2964023 | -1.172601 | 0.5797964 | 1 |
| Andr ́e Bonadias | ATvsUC | -0.16721712 | -0.8291563 | 0.4947221 | 1 |
| Andr ́e Bonadias | HPvsUC | 0.21454728 | -0.1712696 | 0.6003641 | 1 |
| Andr ́e Bonadias | MCvsUC | -0.42130937 | -0.7499101 | -0.0927087 | 1 |
| Andr ́e Bonadias | NSvsUC | 0.06525721 | -0.2776265 | 0.408141 | 1 |
| Andr ́e Bonadias | RTvsUC | -0.20726215 | -0.4876143 | 0.07309 | 1 |
| Andr ́e Bonadias | RT-ATvsUC | -0.19400451 | -0.8560448 | 0.4680358 | 1 |
| Andr ́e Bonadias | RT-HPvsUC | -0.22801875 | -1.071032 | 0.6149948 | 1 |
| Elmoetez Magtouf | ATvsUC | -0.24703193 | -0.9711632 | 0.4770994 | 1 |
| Elmoetez Magtouf | HPvsUC | 0.21454728 | -0.2971132 | 0.7262077 | 1 |
| Elmoetez Magtouf | MCvsUC | -0.43129617 | -0.8801414 | 0.0175491 | 1 |
| Elmoetez Magtouf | NSvsUC | 0.06045347 | -0.3877911 | 0.508698 | 1 |
| Elmoetez Magtouf | RTvsUC | -0.34621153 | -0.6295848 | -0.0628382 | 1 |
| Elmoetez Magtouf | RT-ATvsUC | -0.27383731 | -0.9980635 | 0.4503889 | 1 |
| Elmoetez Magtouf | RT-HPvsUC | -0.2928431 | -1.184863 | 0.5991766 | 1 |
| Hamza Ferhi | ATvsUC | -0.24895176 | -0.9561747 | 0.4582711 | 1 |
| Hamza Ferhi | HPvsUC | 0.21454728 | -0.2662753 | 0.6953699 | 1 |
| Hamza Ferhi | MCvsUC | -0.3191247 | -0.7367962 | 0.0985468 | 1 |
| Hamza Ferhi | NSvsUC | 0.06158497 | -0.3618653 | 0.4850353 | 1 |
| Hamza Ferhi | RTvsUC | -0.34800633 | -0.6243591 | -0.0716535 | 1 |
| Hamza Ferhi | RT-ATvsUC | -0.27575892 | -0.9830784 | 0.4315606 | 1 |
| Hamza Ferhi | RT-HPvsUC | -0.29280674 | -1.171303 | 0.5856891 | 1 |
| Won-Sang Jung | ATvsUC | -0.24807844 | -0.966864 | 0.4707071 | 1 |
| Won-Sang Jung | HPvsUC | 0.21454728 | -0.2875535 | 0.7166481 | 1 |
| Won-Sang Jung | MCvsUC | -0.41231782 | -0.8227559 | -0.0018797 | 1 |
| Won-Sang Jung | NSvsUC | 0.06080023 | -0.3798184 | 0.5014188 | 1 |
| Won-Sang Jung | RTvsUC | -0.34754339 | -0.6284096 | -0.0666772 | 1 |
| Won-Sang Jung | RT-ATvsUC | -0.27488449 | -0.9937655 | 0.4439965 | 1 |
| Won-Sang Jung | RT-HPvsUC | -0.29318026 | -1.18092 | 0.5945596 | 1 |
| Cynthia El Hajj | ATvsUC | -0.24909608 | -0.9667971 | 0.468605 | 1 |
| Cynthia El Hajj | HPvsUC | 0.21454728 | -0.2857278 | 0.7148224 | 1 |
| Cynthia El Hajj | MCvsUC | -0.43115693 | -0.7973402 | -0.0649737 | 1 |
| Cynthia El Hajj | NSvsUC | 7.02E-14 | -1.030447 | 1.030447 | 1 |
| Cynthia El Hajj | RTvsUC | -0.34919096 | -0.629051 | -0.0693309 | 1 |
| Cynthia El Hajj | RT-ATvsUC | -0.27590246 | -0.993699 | 0.4418941 | 1 |
| Cynthia El Hajj | RT-HPvsUC | -0.2938689 | -1.180767 | 0.5930293 | 1 |
| Xian Guo | ATvsUC | -0.2795064 | -0.976954 | 0.4179413 | 1 |
| Xian Guo | HPvsUC | 0.21454728 | -0.2451283 | 0.6742228 | 1 |
| Xian Guo | MCvsUC | -0.43912927 | -0.7918343 | -0.0864243 | 1 |
| Xian Guo | NSvsUC | 0.06238116 | -0.3437664 | 0.4685287 | 1 |
| Xian Guo | RTvsUC | -0.39864962 | -0.6788071 | -0.1184921 | 1 |
| Xian Guo | RT-ATvsUC | -0.30632266 | -1.003869 | 0.3912238 | 1 |
| Xian Guo | RT-HPvsUC | -0.31481807 | -1.184898 | 0.5552615 | 1 |
| Luis Polo-Ferrero | ATvsUC | -0.20907189 | -0.9343249 | 0.5161812 | 1 |
| Luis Polo-Ferrero | HPvsUC | 0.21454728 | -0.2922565 | 0.721351 | 1 |
| Luis Polo-Ferrero | MCvsUC | -0.55827795 | -0.9751947 | -0.1413612 | 1 |
| Luis Polo-Ferrero | NSvsUC | 0.06062918 | -0.3837117 | 0.50497 | 1 |
| Luis Polo-Ferrero | RTvsUC | -0.2811829 | -0.5917097 | 0.0293439 | 1 |
| Luis Polo-Ferrero | RT-ATvsUC | -0.2358678 | -0.9612172 | 0.4894816 | 1 |
| Luis Polo-Ferrero | RT-HPvsUC | -0.26354271 | -1.155237 | 0.6281515 | 1 |

Supplementary Table15.Sensitivity analysis for the GRIP outcome in older adults with sarcopenic obesity

| **dropped_id** | **comparison** | **eff** | **lci** | **uci** | **connected** |
| --- | --- | --- | --- | --- | --- |
| Hung-Ting Chen | ATvsUC | 0.35247612 | -0.9835134 | 1.688466 | 1 |
| Hung-Ting Chen | HPvsUC | 0.61868596 | -0.0251868 | 1.262559 | 1 |
| Hung-Ting Chen | MCvsUC | 0.39860759 | -0.7828932 | 1.580108 | 1 |
| Hung-Ting Chen | MC-NSvsUC | 0.32102021 | -0.5798075 | 1.221848 | 1 |
| Hung-Ting Chen | NSvsUC | 0.93804541 | 0.3245162 | 1.551575 | 1 |
| Hung-Ting Chen | RTvsUC | 1.3904587 | -0.0315578 | 2.812475 | 1 |
| espedita Muscariello | ATvsUC | 0.28665973 | -0.9720379 | 1.545357 | 1 |
| espedita Muscariello | HPvsUC | 0.62586537 | -0.0072373 | 1.258968 | 1 |
| espedita Muscariello | MCvsUC | 0.40164896 | -0.7644078 | 1.567706 | 1 |
| espedita Muscariello | MC-NSvsUC | 0.32293288 | -0.5659541 | 1.21182 | 1 |
| espedita Muscariello | NSvsUC | 0.96680594 | 0.4109668 | 1.522645 | 1 |
| espedita Muscariello | RTvsUC | 0.71192947 | -0.2436659 | 1.667525 | 1 |
| Jinkee Park | ATvsUC | 0.06440057 | -1.198196 | 1.326997 | 1 |
| Jinkee Park | HPvsUC | 0.35247612 | -0.914619 | 1.619571 | 1 |
| Jinkee Park | MCvsUC | 0.60922751 | -0.0033111 | 1.221766 | 1 |
| Jinkee Park | MC-NSvsUC | 0.39446182 | -0.7283054 | 1.517229 | 1 |
| Jinkee Park | NSvsUC | 0.31796846 | -0.5372317 | 1.173169 | 1 |
| Jinkee Park | RTvsUC | 0.90973825 | 0.3647746 | 1.454702 | 1 |
| Jinkee Park | RT-ATvsUC | 0.12926478 | -1.132679 | 1.391209 | 1 |
| Anoop Balachandran | ATvsUC | 0.29935319 | -1.003388 | 1.602095 | 1 |
| Anoop Balachandran | HPvsUC | 0.35247611 | -1.019528 | 1.72448 | 1 |
| Anoop Balachandran | MCvsUC | 0.58203455 | -0.1148433 | 1.278912 | 1 |
| Anoop Balachandran | RTvsUC | 0.99868238 | 0.40028 | 1.597085 | 1 |
| Anoop Balachandran | RT-ATvsUC | 0.71929815 | -0.2703313 | 1.708928 | 1 |
| Shu-Ching Chiu | ATvsUC | 0.17108094 | -0.7697444 | 1.111906 | 1 |
| Shu-Ching Chiu | HPvsUC | 0.35247612 | -0.5347579 | 1.23971 | 1 |
| Shu-Ching Chiu | MCvsUC | 0.53489505 | 0.0728823 | 0.9969078 | 1 |
| Shu-Ching Chiu | MC-NSvsUC | 0.36174351 | -0.4421649 | 1.165652 | 1 |
| Shu-Ching Chiu | NSvsUC | 0.29353069 | -0.3122117 | 0.8992731 | 1 |
| Shu-Ching Chiu | RTvsUC | 0.67147287 | 0.2200682 | 1.122878 | 1 |
| Shu-Ching Chiu | RT-ATvsUC | 0.65019132 | -0.0543112 | 1.354694 | 1 |
| Hunkyung Kim PhD | ATvsUC | 0.30347106 | -1.014698 | 1.62164 | 1 |
| Hunkyung Kim PhD | HPvsUC | 0.35247611 | -1.041281 | 1.746233 | 1 |
| Hunkyung Kim PhD | MCvsUC | 0.83438908 | 0.0669964 | 1.601782 | 1 |
| Hunkyung Kim PhD | MC-NSvsUC | 0.13240488 | -1.255536 | 1.520346 | 1 |
| Hunkyung Kim PhD | NSvsUC | 1.0092183 | 0.4219787 | 1.596458 | 1 |
| Hunkyung Kim PhD | RTvsUC | 0.72164366 | -0.2804405 | 1.723728 | 1 |
| Yu-Hao Lee | ATvsUC | 0.34125948 | -0.9135746 | 1.596094 | 1 |
| Yu-Hao Lee | HPvsUC | 0.35247612 | -0.955626 | 1.660578 | 1 |
| Yu-Hao Lee | MCvsUC | 0.65830654 | 0.0263527 | 1.29026 | 1 |
| Yu-Hao Lee | MC-NSvsUC | 0.41549239 | -0.7422991 | 1.573284 | 1 |
| Yu-Hao Lee | NSvsUC | 0.33196092 | -0.5504419 | 1.214364 | 1 |
| Yu-Hao Lee | RTvsUC | 1.0898503 | 0.493333 | 1.686368 | 1 |
| Yu-Hao Lee | RT-ATvsUC | 0.74783628 | -0.2038724 | 1.699545 | 1 |
| Elmoetez Magtouf | ATvsUC | 0.27303509 | -0.9937276 | 1.539798 | 1 |
| Elmoetez Magtouf | HPvsUC | 0.35247612 | -0.9756518 | 1.680604 | 1 |
| Elmoetez Magtouf | MCvsUC | 0.47919982 | -0.2357032 | 1.194103 | 1 |
| Elmoetez Magtouf | MC-NSvsUC | 0.33900283 | -0.8436201 | 1.521626 | 1 |
| Elmoetez Magtouf | NSvsUC | 0.28188471 | -0.6181572 | 1.181927 | 1 |
| Elmoetez Magtouf | RTvsUC | 0.93651829 | 0.3733371 | 1.499699 | 1 |
| Elmoetez Magtouf | RT-ATvsUC | 0.70282999 | -0.2590328 | 1.664693 | 1 |
| Hamza Ferhi | ATvsUC | 0.28682341 | -1.031349 | 1.604996 | 1 |
| Hamza Ferhi | HPvsUC | 0.35247612 | -1.041485 | 1.746438 | 1 |
| Hamza Ferhi | MCvsUC | 0.65185538 | -0.0915892 | 1.3953 | 1 |
| Hamza Ferhi | MC-NSvsUC | 0.41286015 | -0.8257915 | 1.651512 | 1 |
| Hamza Ferhi | NSvsUC | 0.33062929 | -0.6129475 | 1.274206 | 1 |
| Hamza Ferhi | RTvsUC | 0.97142857 | 0.3854801 | 1.557377 | 1 |
| Hamza Ferhi | RT-ATvsUC | 0.71072789 | -0.2913569 | 1.712813 | 1 |
| Cynthia El Hajj | ATvsUC | 0.28673333 | -1.030117 | 1.603584 | 1 |
| Cynthia El Hajj | HPvsUC | 0.35247612 | -1.040177 | 1.745129 | 1 |
| Cynthia El Hajj | MCvsUC | 0.65047675 | -0.0242678 | 1.325221 | 1 |
| Cynthia El Hajj | MC-NSvsUC | 0.4640369 | -0.8082469 | 1.736321 | 1 |
| Cynthia El Hajj | NSvsUC | 0.48531286 | -0.7882048 | 1.75883 | 1 |
| Cynthia El Hajj | RTvsUC | 0.97115496 | 0.3891915 | 1.553118 | 1 |
| Cynthia El Hajj | RT-ATvsUC | 0.71068997 | -0.2904298 | 1.71181 | 1 |
| Xian Guo | ATvsUC | 0.25643214 | -1.039694 | 1.552559 | 1 |
| Xian Guo | HPvsUC | 0.35247611 | -1.009024 | 1.713977 | 1 |
| Xian Guo | MCvsUC | 0.60933807 | -0.044242 | 1.262918 | 1 |
| Xian Guo | MC-NSvsUC | 0.39464846 | -0.8084353 | 1.597732 | 1 |
| Xian Guo | NSvsUC | 0.31853302 | -0.5990819 | 1.236148 | 1 |
| Xian Guo | RTvsUC | 0.90072981 | 0.2886786 | 1.512781 | 1 |
| Xian Guo | RT-ATvsUC | 0.69135944 | -0.2927752 | 1.675494 | 1 |
| Luis Polo-Ferrero | ATvsUC | 0.3247987 | -1.020824 | 1.670422 | 1 |
| Luis Polo-Ferrero | HPvsUC | 0.35247612 | -1.070936 | 1.775888 | 1 |
| Luis Polo-Ferrero | MCvsUC | 0.63130823 | -0.1273491 | 1.389966 | 1 |
| Luis Polo-Ferrero | MC-NSvsUC | 0.40411543 | -0.8598973 | 1.668128 | 1 |
| Luis Polo-Ferrero | NSvsUC | 0.32500009 | -0.6382173 | 1.288217 | 1 |
| Luis Polo-Ferrero | RTvsUC | 1.0592441 | 0.4151698 | 1.703318 | 1 |
| Luis Polo-Ferrero | RT-ATvsUC | 0.73517383 | -0.287466 | 1.757814 | 1 |
| Chun-De Liao | ATvsUC | 0.34058615 | -0.9215844 | 1.602757 | 1 |
| Chun-De Liao | HPvsUC | 0.35247612 | -0.9641379 | 1.66909 | 1 |
| Chun-De Liao | MCvsUC | 0.65834404 | 0.0225699 | 1.294118 | 1 |
| Chun-De Liao | MC-NSvsUC | 0.41552187 | -0.7495216 | 1.580565 | 1 |
| Chun-De Liao | NSvsUC | 0.33202315 | -0.5560098 | 1.220056 | 1 |
| Chun-De Liao | RTvsUC | 1.0888717 | 0.4826713 | 1.695072 | 1 |
| Chun-De Liao | RT-ATvsUC | 0.74724419 | -0.2101138 | 1.704602 | 1 |

Supplementary Table16.Sensitivity analysis for the FM outcome in older adults with sarcopenic obesity

| **dropped_id** | **comparison** | **eff** | **lci** | **uci** | **connected** |
| --- | --- | --- | --- | --- | --- |
| M Aubertin-Leheudre | ATvsUC | -0.37707053 | -1.088746 | 0.3346045 | 1 |
| M Aubertin-Leheudre | HPvsUC | -0.05212521 | -0.4677862 | 0.3635358 | 1 |
| M Aubertin-Leheudre | MCvsUC | 0.0077997 | -0.4863282 | 0.5019276 | 1 |
| M Aubertin-Leheudre | MC-NSvsUC | -0.01367998 | -0.5047372 | 0.4773773 | 1 |
| M Aubertin-Leheudre | NSvsUC | 0.07938821 | -0.263932 | 0.4227084 | 1 |
| M Aubertin-Leheudre | RTvsUC | -0.35164375 | -0.7096233 | 0.0063358 | 1 |
| M Aubertin-Leheudre | RT-ATvsUC | -0.37707053 | -1.088746 | 0.3346045 | 1 |
| M Aubertin-Leheudre | RT-HPvsUC | -0.35712567 | -1.005794 | 0.2915428 | 1 |
| Ebrahim Banitalebi | ATvsUC | -0.21793364 | -0.8990701 | 0.4632029 | 1 |
| Ebrahim Banitalebi | HPvsUC | -0.05211221 | -0.4073008 | 0.3030764 | 1 |
| Ebrahim Banitalebi | MCvsUC | 0.00293104 | -0.4274887 | 0.4333508 | 1 |
| Ebrahim Banitalebi | MC-NSvsUC | -0.01854874 | -0.4454403 | 0.4083428 | 1 |
| Ebrahim Banitalebi | NSvsUC | 0.06960395 | -0.2079006 | 0.3471085 | 1 |
| Ebrahim Banitalebi | RT-HPvsUC | -0.1603644 | -0.7932582 | 0.4725294 | 1 |
| Hung-Ting Chen | ATvsUC | -0.05212363 | -0.4603849 | 0.3561376 | 1 |
| Hung-Ting Chen | HPvsUC | -0.00009763 | -0.4838021 | 0.4836068 | 1 |
| Hung-Ting Chen | MCvsUC | -0.02157699 | -0.5021443 | 0.4589904 | 1 |
| Hung-Ting Chen | MC-NSvsUC | 0.06375323 | -0.258008 | 0.3855144 | 1 |
| Hung-Ting Chen | NSvsUC | -0.37854185 | -0.7702939 | 0.0132102 | 1 |
| Hung-Ting Chen | RTvsUC | -0.37721932 | -1.034043 | 0.2796041 | 1 |
| shih-Wei huanG | ATvsUC | -0.39359129 | -1.117923 | 0.3307405 | 1 |
| shih-Wei huanG | HPvsUC | -0.05212544 | -0.4688863 | 0.3646354 | 1 |
| shih-Wei huanG | MCvsUC | -0.00053771 | -0.4930725 | 0.4919971 | 1 |
| shih-Wei huanG | MC-NSvsUC | -0.02201701 | -0.5114713 | 0.4674373 | 1 |
| shih-Wei huanG | NSvsUC | 0.06289951 | -0.2654745 | 0.3912735 | 1 |
| shih-Wei huanG | RTvsUC | -0.37955579 | -0.796842 | 0.0377304 | 1 |
| shih-Wei huanG | RT-ATvsUC | -0.39359129 | -1.117923 | 0.3307405 | 1 |
| shih-Wei huanG | RT-HPvsUC | -0.37777419 | -1.04676 | 0.2912115 | 1 |
| Mathieu L | ATvsUC | -0.43094341 | -1.121012 | 0.259125 | 1 |
| Mathieu L | HPvsUC | -0.052116 | -0.4249006 | 0.3206686 | 1 |
| Mathieu L | MCvsUC | 0.00186819 | -0.4463629 | 0.4500993 | 1 |
| Mathieu L | MC-NSvsUC | -0.01961143 | -0.4644554 | 0.4252325 | 1 |
| Mathieu L | NSvsUC | 0.0675556 | -0.2281257 | 0.3632369 | 1 |
| Mathieu L | RTvsUC | -0.44003813 | -0.8100123 | -0.070064 | 1 |
| Mathieu L | RT-ATvsUC | -0.43094341 | -1.121012 | 0.259125 | 1 |
| Mathieu L | RT-HPvsUC | -0.55724867 | -1.421201 | 0.3067036 | 1 |
| espedita Muscariello | ATvsUC | -0.37385736 | -1.095431 | 0.347716 | 1 |
| espedita Muscariello | HPvsUC | -0.05238705 | -1.020278 | 0.9155037 | 1 |
| espedita Muscariello | MCvsUC | -0.00121699 | -0.507946 | 0.5055121 | 1 |
| espedita Muscariello | MC-NSvsUC | -0.02269621 | -0.5264316 | 0.4810392 | 1 |
| espedita Muscariello | NSvsUC | 0.06157992 | -0.2768703 | 0.4000301 | 1 |
| espedita Muscariello | RTvsUC | -0.34671446 | -0.7086323 | 0.0152034 | 1 |
| espedita Muscariello | RT-ATvsUC | -0.37385736 | -1.095431 | 0.347716 | 1 |
| espedita Muscariello | RT-HPvsUC | -0.35316148 | -1.009009 | 0.3026865 | 1 |
| Hellen C.G. Nabuco | ATvsUC | -0.38535931 | -1.092107 | 0.3213883 | 1 |
| Hellen C.G. Nabuco | HPvsUC | -0.05212331 | -0.4588703 | 0.3546237 | 1 |
| Hellen C.G. Nabuco | MCvsUC | -0.00001806 | -0.4821342 | 0.4820981 | 1 |
| Hellen C.G. Nabuco | MC-NSvsUC | -0.02149742 | -0.5004661 | 0.4574713 | 1 |
| Hellen C.G. Nabuco | NSvsUC | 0.0639075 | -0.2565308 | 0.3843458 | 1 |
| Hellen C.G. Nabuco | RTvsUC | -0.36528283 | -0.725398 | -0.0051677 | 1 |
| Hellen C.G. Nabuco | RT-ATvsUC | -0.38535931 | -1.092107 | 0.3213883 | 1 |
| Hellen C.G. Nabuco | RT-HPvsUC | -0.2366106 | -1.108066 | 0.6348452 | 1 |
| Rosa Sammarco | ATvsUC | -0.37385736 | -1.095431 | 0.3477161 | 1 |
| Rosa Sammarco | HPvsUC | -0.05206457 | -0.532459 | 0.4283299 | 1 |
| Rosa Sammarco | MCvsUC | -0.00121699 | -0.507946 | 0.5055121 | 1 |
| Rosa Sammarco | MC-NSvsUC | -0.02269621 | -0.5264316 | 0.4810392 | 1 |
| Rosa Sammarco | NSvsUC | 0.06157992 | -0.2768703 | 0.4000301 | 1 |
| Rosa Sammarco | RTvsUC | -0.34671446 | -0.7086325 | 0.0152036 | 1 |
| Rosa Sammarco | RT-ATvsUC | -0.37385736 | -1.095431 | 0.3477161 | 1 |
| Rosa Sammarco | RT-HPvsUC | -0.35316148 | -1.00901 | 0.3026866 | 1 |
| Hunkyung Kim PhD | ATvsUC | -0.36620043 | -1.114303 | 0.3819023 | 1 |
| Hunkyung Kim PhD | HPvsUC | -0.052136 | -0.5196641 | 0.4153921 | 1 |
| Hunkyung Kim PhD | MCvsUC | 0.08034545 | -0.3853646 | 0.5460555 | 1 |
| Hunkyung Kim PhD | MC-NSvsUC | -0.33487986 | -0.706121 | 0.0363613 | 1 |
| Hunkyung Kim PhD | NSvsUC | -0.36620043 | -1.114303 | 0.3819023 | 1 |
| Hunkyung Kim PhD | RTvsUC | -0.34364027 | -1.018483 | 0.3312028 | 1 |
| Cynthia El Hajj | ATvsUC | -0.36639882 | -1.113736 | 0.3809379 | 1 |
| Cynthia El Hajj | HPvsUC | -0.0521358 | -0.5186518 | 0.4143803 | 1 |
| Cynthia El Hajj | MCvsUC | -0.03400216 | -0.6102811 | 0.5422767 | 1 |
| Cynthia El Hajj | MC-NSvsUC | -0.05548005 | -0.6291262 | 0.5181661 | 1 |
| Cynthia El Hajj | NSvsUC | -0.00334442 | -0.5208222 | 0.5141333 | 1 |
| Cynthia El Hajj | RTvsUC | -0.33518822 | -0.7060968 | 0.0357203 | 1 |
| Cynthia El Hajj | RT-ATvsUC | -0.36639882 | -1.113736 | 0.3809379 | 1 |
| Cynthia El Hajj | RT-HPvsUC | -0.34388839 | -1.018168 | 0.3303914 | 1 |
| Xian Guo | ATvsUC | -0.44454671 | -1.116544 | 0.2274505 | 1 |
| Xian Guo | HPvsUC | -0.05211221 | -0.4073008 | 0.3030764 | 1 |
| Xian Guo | MCvsUC | 0.00293105 | -0.4274887 | 0.4333508 | 1 |
| Xian Guo | MC-NSvsUC | -0.01854873 | -0.4454403 | 0.4083429 | 1 |
| Xian Guo | NSvsUC | 0.06960396 | -0.2079006 | 0.3471085 | 1 |
| Xian Guo | RTvsUC | -0.46164811 | -0.796739 | -0.1265572 | 1 |
| Xian Guo | RT-ATvsUC | -0.44454671 | -1.116544 | 0.2274505 | 1 |
| Xian Guo | RT-HPvsUC | -0.44024883 | -1.05806 | 0.177562 | 1 |

Supplementary Table17.Sensitivity analysis for the PBF outcome in older adults with sarcopenic obesity

| **dropped_id** | **comparison** | **eff** | **lci** | **uci** | **connected** |
| --- | --- | --- | --- | --- | --- |
| Hung-Ting Chen | ATvsUC | -0.2249872 | -1.743665 | 1.29369 | 1 |
| Hung-Ting Chen | HPvsUC | -0.62732451 | -1.159908 | -0.0947409 | 1 |
| Hung-Ting Chen | MCvsUC | -0.28895231 | -1.401225 | 0.8233202 | 1 |
| Hung-Ting Chen | MC-NSvsUC | -0.07885902 | -0.9275203 | 0.7698023 | 1 |
| Hung-Ting Chen | NSvsUC | -0.66184869 | -1.0854 | -0.2382974 | 1 |
| Hung-Ting Chen | RTvsUC | -0.61393933 | -1.943923 | 0.716044 | 1 |
| Hung-Ting Chen | RT-ATvsUC | -0.90367475 | -2.394047 | 0.5866977 | 1 |
| shih-Wei huanG | ATvsUC | -0.62361701 | -1.798359 | 0.5511248 | 1 |
| shih-Wei huanG | HPvsUC | -0.2249872 | -1.710197 | 1.260223 | 1 |
| shih-Wei huanG | MCvsUC | -0.62323541 | -1.141577 | -0.1048938 | 1 |
| shih-Wei huanG | MC-NSvsUC | -0.28709302 | -1.365326 | 0.7911399 | 1 |
| shih-Wei huanG | NSvsUC | -0.0773208 | -0.8994423 | 0.7448007 | 1 |
| shih-Wei huanG | RTvsUC | -0.65780362 | -1.068285 | -0.2473219 | 1 |
| shih-Wei huanG | RT-ATvsUC | -0.62015607 | -1.504934 | 0.2646217 | 1 |
| shih-Wei huanG | RT-HPvsUC | -0.89964094 | -2.352161 | 0.5528787 | 1 |
| Chun-De Liao | ATvsUC | -0.64342669 | -1.791247 | 0.5043935 | 1 |
| Chun-De Liao | HPvsUC | -0.2249872 | -1.681064 | 1.231089 | 1 |
| Chun-De Liao | MCvsUC | -0.63561485 | -1.141845 | -0.1293851 | 1 |
| Chun-De Liao | MC-NSvsUC | -0.29226993 | -1.340649 | 0.7561088 | 1 |
| Chun-De Liao | NSvsUC | -0.08039399 | -0.8792194 | 0.7184314 | 1 |
| Chun-De Liao | RTvsUC | -0.70114314 | -1.10345 | -0.298836 | 1 |
| Chun-De Liao | RT-ATvsUC | -0.6328441 | -1.496189 | 0.2305009 | 1 |
| Chun-De Liao | RT-HPvsUC | -0.94297867 | -2.363369 | 0.477412 | 1 |
| Hellen C.G. Nabuco | ATvsUC | -0.61566616 | -1.760248 | 0.5289159 | 1 |
| Hellen C.G. Nabuco | HPvsUC | -0.2249872 | -1.67904 | 1.229066 | 1 |
| Hellen C.G. Nabuco | MCvsUC | -0.61411388 | -1.117643 | -0.1105845 | 1 |
| Hellen C.G. Nabuco | MC-NSvsUC | -0.28308196 | -1.329213 | 0.7630489 | 1 |
| Hellen C.G. Nabuco | RT-ATvsUC | -0.61504627 | -1.476154 | 0.2460617 | 1 |
| Jinkee Park | ATvsUC | -0.61585015 | -1.831611 | 0.5999103 | 1 |
| Jinkee Park | HPvsUC | -0.2249872 | -1.713419 | 1.263445 | 1 |
| Jinkee Park | MCvsUC | -0.61731692 | -1.135568 | -0.0990658 | 1 |
| Jinkee Park | MC-NSvsUC | -0.28457683 | -1.365971 | 0.7968177 | 1 |
| Jinkee Park | NSvsUC | -0.07570717 | -0.9003212 | 0.7489069 | 1 |
| Jinkee Park | RTvsUC | -0.63987745 | -1.036259 | -0.2434957 | 1 |
| Jinkee Park | RT-ATvsUC | -0.61585015 | -1.831611 | 0.5999103 | 1 |
| Jinkee Park | RT-HPvsUC | -0.88171836 | -2.333621 | 0.5701842 | 1 |
| Rosa Sammarco | ATvsUC | -0.61565325 | -1.76022 | 0.5289135 | 1 |
| Rosa Sammarco | HPvsUC | -0.61410237 | -1.117624 | -0.1105809 | 1 |
| Rosa Sammarco | MCvsUC | -0.28307698 | -1.329191 | 0.7630373 | 1 |
| Rosa Sammarco | MC-NSvsUC | -0.07436539 | -0.8714534 | 0.7227226 | 1 |
| Rosa Sammarco | NSvsUC | -0.63927558 | -1.021369 | -0.2571823 | 1 |
| Rosa Sammarco | RTvsUC | -0.61503799 | -1.476134 | 0.2460579 | 1 |
| Rosa Sammarco | RT-ATvsUC | -0.88112665 | -2.293829 | 0.5315755 | 1 |
| Anoop Balachandran | ATvsUC | -0.61486962 | -1.785812 | 0.5560728 | 1 |
| Anoop Balachandran | HPvsUC | -0.2249872 | -1.706946 | 1.256972 | 1 |
| Anoop Balachandran | MCvsUC | -0.61995233 | -1.157485 | -0.0824192 | 1 |
| Anoop Balachandran | MC-NSvsUC | -0.28567939 | -1.362431 | 0.7910723 | 1 |
| Anoop Balachandran | NSvsUC | -0.07636325 | -0.8969312 | 0.7442047 | 1 |
| Anoop Balachandran | RTvsUC | -0.63822105 | -1.036206 | -0.2402366 | 1 |
| Anoop Balachandran | RT-ATvsUC | -0.6145368 | -1.496491 | 0.2674171 | 1 |
| Anoop Balachandran | RT-HPvsUC | -0.88006428 | -2.325773 | 0.5656444 | 1 |
| Shu-Ching Chiu | ATvsUC | -0.57145744 | -1.578459 | 0.4355436 | 1 |
| Shu-Ching Chiu | HPvsUC | -0.2249872 | -1.532166 | 1.082192 | 1 |
| Shu-Ching Chiu | MCvsUC | -0.80717005 | -1.275593 | -0.338747 | 1 |
| Shu-Ching Chiu | MC-NSvsUC | -0.36461406 | -1.258128 | 0.5288994 | 1 |
| Shu-Ching Chiu | NSvsUC | -0.12502468 | -0.8021325 | 0.5520832 | 1 |
| Shu-Ching Chiu | RTvsUC | -0.5375677 | -0.8782243 | -0.1969111 | 1 |
| Shu-Ching Chiu | RT-ATvsUC | -0.58706543 | -1.337994 | 0.1638633 | 1 |
| Shu-Ching Chiu | RT-HPvsUC | -0.77947797 | -2.028632 | 0.4696757 | 1 |
| Hunkyung Kim PhD | ATvsUC | -0.62898904 | -1.800686 | 0.542708 | 1 |
| Hunkyung Kim PhD | HPvsUC | -0.2249872 | -1.708006 | 1.258032 | 1 |
| Hunkyung Kim PhD | MCvsUC | -0.76666102 | -1.340487 | -0.1928351 | 1 |
| Hunkyung Kim PhD | MC-NSvsUC | 0.18987922 | -1.023932 | 1.40369 | 1 |
| Hunkyung Kim PhD | NSvsUC | -0.6697257 | -1.064727 | -0.2747242 | 1 |
| Hunkyung Kim PhD | RTvsUC | -0.62360557 | -1.506215 | 0.2590036 | 1 |
| Hunkyung Kim PhD | RT-ATvsUC | -0.91156068 | -2.357538 | 0.5344167 | 1 |
| Chun-De Liao | ATvsUC | -0.63017884 | -1.800727 | 0.5403688 | 1 |
| Chun-De Liao | HPvsUC | -0.2249872 | -1.705579 | 1.255604 | 1 |
| Chun-De Liao | MCvsUC | -0.62785181 | -1.144354 | -0.11135 | 1 |
| Chun-De Liao | MC-NSvsUC | -0.28904788 | -1.362571 | 0.7844755 | 1 |
| Chun-De Liao | NSvsUC | -0.07855221 | -0.8969983 | 0.739894 | 1 |
| Chun-De Liao | RTvsUC | -0.67231506 | -1.082439 | -0.2621908 | 1 |
| Chun-De Liao | RT-ATvsUC | -0.62436811 | -1.505792 | 0.2570563 | 1 |
| Chun-De Liao | RT-HPvsUC | -0.91415013 | -2.361848 | 0.5335473 | 1 |
| Ebrahim Banitalebi | ATvsUC | -0.6471867 | -1.786308 | 0.4919348 | 1 |
| Ebrahim Banitalebi | HPvsUC | -0.2249872 | -1.671678 | 1.221703 | 1 |
| Ebrahim Banitalebi | MCvsUC | -0.63752107 | -1.139813 | -0.1352292 | 1 |
| Ebrahim Banitalebi | MC-NSvsUC | -0.293046 | -1.33175 | 0.7456582 | 1 |
| Ebrahim Banitalebi | NSvsUC | -0.08079326 | -0.8720659 | 0.7104794 | 1 |
| Ebrahim Banitalebi | RTvsUC | -0.70922742 | -1.108809 | -0.309646 | 1 |
| Ebrahim Banitalebi | RT-ATvsUC | -0.6352404 | -1.491653 | 0.2211722 | 1 |
| Ebrahim Banitalebi | RT-HPvsUC | -0.95106394 | -2.361059 | 0.4589316 | 1 |
| Paolo M. Cunha | ATvsUC | -0.63776301 | -1.795993 | 0.5204672 | 1 |
| Paolo M. Cunha | HPvsUC | -0.2249872 | -1.69234 | 1.242366 | 1 |
| Paolo M. Cunha | MCvsUC | -0.63240198 | -1.143326 | -0.1214777 | 1 |
| Paolo M. Cunha | MC-NSvsUC | -0.29094181 | -1.350905 | 0.7690219 | 1 |
| Paolo M. Cunha | NSvsUC | -0.07965065 | -0.8875186 | 0.7282172 | 1 |
| Paolo M. Cunha | RTvsUC | -0.68885649 | -1.094059 | -0.2836541 | 1 |
| Paolo M. Cunha | RT-ATvsUC | -0.62922499 | -1.500867 | 0.2424176 | 1 |
| Paolo M. Cunha | RT-HPvsUC | -0.93069152 | -2.363451 | 0.5020686 | 1 |
| Andr ́e Bonadias | ATvsUC | -0.58357273 | -1.721178 | 0.5540327 | 1 |
| Andr ́e Bonadias | HPvsUC | -0.2249872 | -1.669925 | 1.219951 | 1 |
| Andr ́e Bonadias | MCvsUC | -0.58859374 | -1.090736 | -0.0864518 | 1 |
| Andr ́e Bonadias | MC-NSvsUC | -0.2721506 | -1.3091 | 0.7647986 | 1 |
| Andr ́e Bonadias | NSvsUC | -0.06712761 | -0.8570225 | 0.7227673 | 1 |
| Andr ́e Bonadias | RTvsUC | -0.56769113 | -0.9687501 | -0.1666321 | 1 |
| Andr ́e Bonadias | RT-ATvsUC | -0.59448641 | -1.449663 | 0.2606905 | 1 |
| Andr ́e Bonadias | RT-HPvsUC | -0.80956178 | -2.218178 | 0.5990548 | 1 |
| Yu-Hao Lee | ATvsUC | -0.63349797 | -1.795371 | 0.5283751 | 1 |
| Yu-Hao Lee | HPvsUC | -0.2249872 | -1.696432 | 1.246458 | 1 |
| Yu-Hao Lee | MCvsUC | -0.62952623 | -1.142034 | -0.1170185 | 1 |
| Yu-Hao Lee | MC-NSvsUC | -0.28972926 | -1.353878 | 0.7744191 | 1 |
| Yu-Hao Lee | NSvsUC | -0.07890194 | -0.8900385 | 0.7322346 | 1 |
| Yu-Hao Lee | RTvsUC | -0.67946832 | -1.083921 | -0.2750153 | 1 |
| Yu-Hao Lee | RT-ATvsUC | -0.62649196 | -1.50107 | 0.2480859 | 1 |
| Yu-Hao Lee | RT-HPvsUC | -0.92130441 | -2.358042 | 0.5154335 | 1 |
| Elmoetez Magtouf | ATvsUC | -0.60542417 | -1.740007 | 0.5291588 | 1 |
| Elmoetez Magtouf | HPvsUC | -0.2249872 | -1.668128 | 1.218153 | 1 |
| Elmoetez Magtouf | MCvsUC | -0.4983639 | -1.042201 | 0.0454735 | 1 |
| Elmoetez Magtouf | MC-NSvsUC | -0.23362285 | -1.272617 | 0.8053709 | 1 |
| Elmoetez Magtouf | NSvsUC | -0.04195004 | -0.8325818 | 0.7486817 | 1 |
| Elmoetez Magtouf | RTvsUC | -0.61624627 | -0.9971045 | -0.235388 | 1 |
| Elmoetez Magtouf | RT-ATvsUC | -0.60848522 | -1.461595 | 0.2446246 | 1 |
| Elmoetez Magtouf | RT-HPvsUC | -0.8581059 | -2.259257 | 0.543045 | 1 |
| Hamza Ferhi | ATvsUC | -0.59616867 | -1.605871 | 0.4135337 | 1 |
| Hamza Ferhi | HPvsUC | -0.2249872 | -1.535495 | 1.08552 | 1 |
| Hamza Ferhi | MCvsUC | -0.36889922 | -0.8423089 | 0.1045105 | 1 |
| Hamza Ferhi | MC-NSvsUC | -0.17780161 | -1.075257 | 0.7196533 | 1 |
| Hamza Ferhi | NSvsUC | -0.00403564 | -0.6840922 | 0.6760209 | 1 |
| Hamza Ferhi | RTvsUC | -0.59205483 | -0.9267868 | -0.2573228 | 1 |
| Hamza Ferhi | RT-ATvsUC | -0.60269343 | -1.355923 | 0.1505359 | 1 |
| Hamza Ferhi | RT-HPvsUC | -0.83395405 | -2.084993 | 0.4170845 | 1 |
| Won-Sang Jung | ATvsUC | -0.60904211 | -1.763753 | 0.5456686 | 1 |
| Won-Sang Jung | HPvsUC | -0.2249872 | -1.689789 | 1.239815 | 1 |
| Won-Sang Jung | MCvsUC | -0.54190368 | -1.087496 | 0.0036887 | 1 |
| Won-Sang Jung | MC-NSvsUC | -0.25229022 | -1.312881 | 0.8083006 | 1 |
| Won-Sang Jung | NSvsUC | -0.05436158 | -0.8619984 | 0.7532753 | 1 |
| Won-Sang Jung | RTvsUC | -0.62482474 | -1.012545 | -0.2371048 | 1 |
| Won-Sang Jung | RT-ATvsUC | -0.61079774 | -1.479928 | 0.2583326 | 1 |
| Won-Sang Jung | RT-HPvsUC | -0.86667623 | -2.291972 | 0.5586196 | 1 |
| Cynthia El Hajj | ATvsUC | -0.6177462 | -1.787718 | 0.5522254 | 1 |
| Cynthia El Hajj | HPvsUC | -0.2249872 | -1.706358 | 1.256383 | 1 |
| Cynthia El Hajj | MCvsUC | -0.64117751 | -1.162633 | -0.1197218 | 1 |
| Cynthia El Hajj | MC-NSvsUC | -0.36686815 | -1.476436 | 0.7427 | 1 |
| Cynthia El Hajj | NSvsUC | -0.29837528 | -1.409303 | 0.8125525 | 1 |
| Cynthia El Hajj | RTvsUC | -0.64461964 | -1.036176 | -0.2530634 | 1 |
| Cynthia El Hajj | RT-ATvsUC | -0.61638417 | -1.497672 | 0.2649034 | 1 |
| Cynthia El Hajj | RT-HPvsUC | -0.88646143 | -2.329811 | 0.5568878 | 1 |
| Xian Guo | ATvsUC | -0.60280186 | -1.768667 | 0.5630634 | 1 |
| Xian Guo | HPvsUC | -0.2249872 | -1.700809 | 1.250835 | 1 |
| Xian Guo | MCvsUC | -0.60634755 | -1.120417 | -0.0922777 | 1 |
| Xian Guo | MC-NSvsUC | -0.27984762 | -1.348453 | 0.7887579 | 1 |
| Xian Guo | NSvsUC | -0.07248704 | -0.8871014 | 0.7421274 | 1 |
| Xian Guo | RTvsUC | -0.61117997 | -1.015979 | -0.2063808 | 1 |
| Xian Guo | RT-ATvsUC | -0.60678927 | -1.484558 | 0.2709792 | 1 |
| Xian Guo | RT-HPvsUC | -0.85303171 | -2.294349 | 0.5882852 | 1 |
| Luis Polo-Ferrero | ATvsUC | -0.59117613 | -1.77095 | 0.5885978 | 1 |
| Luis Polo-Ferrero | HPvsUC | -0.2249872 | -1.715727 | 1.265753 | 1 |
| Luis Polo-Ferrero | MCvsUC | -0.63508961 | -1.197879 | -0.0723001 | 1 |
| Luis Polo-Ferrero | MC-NSvsUC | -0.29217506 | -1.379888 | 0.7955375 | 1 |
| Luis Polo-Ferrero | NSvsUC | -0.08070097 | -0.9093896 | 0.7479876 | 1 |
| Luis Polo-Ferrero | RTvsUC | -0.58557991 | -0.9965099 | -0.1746499 | 1 |
| Luis Polo-Ferrero | RT-ATvsUC | -0.59931153 | -1.488109 | 0.2894861 | 1 |
| Luis Polo-Ferrero | RT-HPvsUC | -0.82743396 | -2.285733 | 0.6308647 | 1 |

Supplementary Table18.Sensitivity analysis for the SMI outcome in older adults with sarcopenic obesity

| **dropped_id** | **comparison** | **eff** | **lci** | **uci** | **connected** |
| --- | --- | --- | --- | --- | --- |
| shih-Wei huanG | ATvsUC | -0.20804349 | -0.6392767 | 0.2231897 | 1 |
| shih-Wei huanG | MC-NSvsUC | 0.01177624 | -0.4465162 | 0.4700687 | 1 |
| shih-Wei huanG | NSvsUC | -0.11629469 | -0.5781621 | 0.3455727 | 1 |
| shih-Wei huanG | RTvsUC | 0.09211316 | -0.3119934 | 0.4962198 | 1 |
| Chun-De Liao | ATvsUC | -0.22186448 | -0.6549695 | 0.2112405 | 1 |
| Chun-De Liao | MC-NSvsUC | 0.00479487 | -0.4539478 | 0.4635375 | 1 |
| Chun-De Liao | NSvsUC | -0.12330802 | -0.5856262 | 0.3390102 | 1 |
| Chun-De Liao | RTvsUC | 0.02229372 | -0.4300216 | 0.474609 | 1 |
| Anoop Balachandran | ATvsUC | -0.28213294 | -0.7553 | 0.1910342 | 1 |
| Anoop Balachandran | MC-NSvsUC | -0.02564845 | -0.4943795 | 0.4430826 | 1 |
| Anoop Balachandran | NSvsUC | -0.15389069 | -0.6262116 | 0.3184302 | 1 |
| Anoop Balachandran | RTvsUC | 0.15545224 | -0.2095156 | 0.5204201 | 1 |
| Yu-Hao Lee | ATvsUC | -0.19913486 | -0.6297739 | 0.2315041 | 1 |
| Yu-Hao Lee | MC-NSvsUC | 0.01627625 | -0.4418736 | 0.4744262 | 1 |
| Yu-Hao Lee | NSvsUC | -0.11177409 | -0.5734987 | 0.3499506 | 1 |
| Yu-Hao Lee | RTvsUC | 0.13711695 | -0.250481 | 0.5247149 | 1 |
| Hunkyung Kim PhD | ATvsUC | 0.15542959 | -0.8644549 | 1.175314 | 1 |
| Hunkyung Kim PhD | MC-NSvsUC | 0.15544686 | -0.2095147 | 0.5204084 | 1 |

Supplementary Table19.Meta-regression analysis of the BMI outcome with country as a moderator in older adults with sarcopenic obesity

| **Intervention** | **Covariate** | **Coefficient** | **Standard** Error | **Z-statistic** | **P>z** | **lower confidence interval** | **upper confidence interval** |
| --- | --- | --- | --- | --- | --- | --- | --- |
| ATvsHP | Intercept | 0.7030653 | 4.356864 | 0.16 | 0.872 | -7.836231 | 9.242362 |
| ATvsMC | Country | 0.3725878 | 1.506335 | 0.25 | 0.805 | -2.579774 | 3.324949 |
|  | Intercept | -4.060111 | 4.56448 | -0.89 | 0.374 | -13.00633 | 4.886104 |
| ATvsNS | Country | 0.065293 | 1.47779 | 0.04 | 0.965 | -2.831122 | 2.961708 |
|  | Intercept | 0.0632587 | 3.641442 | 0.02 | 0.986 | -7.073837 | 7.200354 |
| ATvsRT | Country | -0.1168985 | 1.468926 | -0.08 | 0.937 | -2.99594 | 2.762143 |
|  | Intercept | 0.5070941 | 3.465841 | 0.15 | 0.884 | -6.285829 | 7.300017 |
| ATvsRT-AT | Intercept | -0.1002977 | 1.278107 | -0.08 | 0.937 | -2.60534 | 2.404745 |
| ATvsRT-HP | Intercept | -0.1936178 | 2.524448 | -0.08 | 0.939 | -5.141444 | 4.754208 |
| ATvsUC | Country | 0.0186243 | 1.468562 | 0.01 | 0.99 | -2.859705 | 2.896954 |
|  | Intercept | 0.1099553 | 3.46245 | 0.03 | 0.975 | -6.676323 | 6.896233 |

Supplementary Table20.Meta-regression analysis of the BMI outcome with follow-up duration as a moderator in older adults with sarcopenic obesity

| **Intervention** | **Covariate** | **Coefficient** | **Standard Error** | **Z-statistic** | **P>z** | **lower confidence interval** | **upper confidence interval** |
| --- | --- | --- | --- | --- | --- | --- | --- |
| ATvsHP | Intercept | 0.817621 | 1.43601 | 0.57 | 0.569 | -1.996907 | 3.632149 |
| ATvsMC | Follow-up duration | -0.0006976 | 2.030078 | 0 | 1 | -3.979578 | 3.978183 |
|  | Intercept | -0.8319474 | 6.571881 | -0.13 | 0.899 | -13.7126 | 12.0487 |
| ATvsNS | Intercept | 0.395217 | 5.953546 | 0.07 | 0.947 | -11.27352 | 12.06395 |
| ATvsRT | Follow-up duration | -0.0943128 | 2.016994 | -0.05 | 0.963 | -4.047549 | 3.858923 |
|  | Intercept | 0.3720187 | 6.467818 | 0.06 | 0.954 | -12.30467 | 13.04871 |
| ATvsRT-AT | Intercept | -0.1000729 | 1.284244 | -0.08 | 0.938 | -2.617144 | 2.416999 |
| ATvsRT-HP | Intercept | -0.3229132 | 2.526495 | -0.13 | 0.898 | -5.274752 | 4.628925 |
| ATvsUC | Follow-up duration | -0.0386472 | 2.016633 | -0.02 | 0.985 | -3.991176 | 3.913881 |
|  | Intercept | 0.4335781 | 6.464487 | 0.07 | 0.947 | -12.23658 | 13.10374 |

Supplementary Table21.Meta-regression analysis of the BMI outcome with mean age as a moderator in older adults with sarcopenic obesity

| **Intervention** | **Covariate** | **Coefficient** | **Standard Error** | **Z-statistic** | **P>z** | **lower confidence interval** | **upper confidence interval** |
| --- | --- | --- | --- | --- | --- | --- | --- |
| ATvsHP | Intercept | 0.4555024 | 8.530627 | 0.05 | 0.957 | -16.26422 | 17.17522 |
| ATvsMC | Mean age | 0.47578 | 1.817622 | 0.26 | 0.794 | -3.086693 | 4.038253 |
|  | Intercept | -36.05353 | 128.005 | -0.28 | 0.778 | -286.9388 | 214.8317 |
| ATvsNS | Mean age | 0.126542 | 1.733105 | 0.07 | 0.942 | -3.270282 | 3.523366 |
|  | Intercept | -8.07365 | 121.0698 | -0.07 | 0.947 | -245.3661 | 229.2188 |
| ATvsRT | Mean age | -0.0042997 | 1.73117 | 0 | 0.998 | -3.397331 | 3.388732 |
|  | Intercept | 0.2433299 | 120.9577 | 0 | 0.998 | -236.8293 | 237.316 |
| ATvsRT-AT | Intercept | -0.1015267 | 1.243857 | -0.08 | 0.935 | -2.539442 | 2.336388 |
| ATvsRT-HP | Intercept | -0.4967575 | 8.613703 | -0.06 | 0.954 | -17.37931 | 16.38579 |
| ATvsUC | Mean age | 0.0985531 | 1.730748 | 0.06 | 0.955 | -3.293651 | 3.490757 |
|  | Intercept | -6.450446 | 120.9293 | -0.05 | 0.957 | -243.4676 | 230.5667 |

Supplementary Table22.Meta-regression analysis of the GRIP outcome with country as a moderator in older adults with sarcopenic obesity

| **Intervention** | **Covariate** | **Coefficient** | **Standard Error** | **Z-statistic** | **P>z** | **lower confidence interval** | **upper confidence interval** |
| --- | --- | --- | --- | --- | --- | --- | --- |
| ATvsHP | Intercept | 2.578665 | 9.216849 | 0.28 | 0.78 | -15.48603 | 20.64336 |
| ATvsMC | Country | -0.4116744 | 8.655237 | -0.05 | 0.962 | -17.37563 | 16.55228 |
|  | Intercept | 3.369997 | 10.28854 | 0.33 | 0.743 | -16.79517 | 23.53516 |
| ATvsMC-NS | Intercept | 1.056763 | 34.51796 | 0.03 | 0.976 | -66.59719 | 68.71072 |
| ATvsNS | Country | -0.7554317 | 8.68942 | -0.09 | 0.931 | -17.78638 | 16.27552 |
|  | Intercept | 4.933922 | 11.41003 | 0.43 | 0.665 | -17.42933 | 27.29717 |
| ATvsRT | Country | -0.7339602 | 8.634053 | -0.09 | 0.932 | -17.65639 | 16.18847 |
|  | Intercept | 6.12459 | 9.315499 | 0.66 | 0.511 | -12.13345 | 24.38263 |
| ATvsRT-AT | Country | 1.771717 | 8.779535 | 0.2 | 0.84 | -15.43586 | 18.97929 |
|  | Intercept | -1.280092 | 9.757005 | -0.13 | 0.896 | -20.40347 | 17.84329 |
| ATvsUC | Country | -0.4430745 | 8.632066 | -0.05 | 0.959 | -17.36161 | 16.47546 |
|  | Intercept | 1.664961 | 9.345932 | 0.18 | 0.859 | -16.65273 | 19.98265 |

Supplementary Table23.Meta-regression analysis of the GRIP outcome with follow-up duration as a moderator in older adults with sarcopenic obesity

| **Intervention** | **Covariate** | **Coefficient** | **Standard Error** | **Z-statistic** | **P>z** | **lower confidence interval** | **upper confidence interval** |
| --- | --- | --- | --- | --- | --- | --- | --- |
| ATvsHP | Intercept | 2.577034 | 3.660752 | 0.7 | 0.481 | -4.597907 | 9.751975 |
| ATvsMC | Follow-up duration | -0.7300553 | 13.29267 | -0.05 | 0.956 | -26.7832 | 25.32309 |
|  | Intercept | 5.048444 | 40.26825 | 0.13 | 0.9 | -73.87588 | 83.97277 |
| ATvsMC-NS | Intercept | 2.500157 | 3.604507 | 0.69 | 0.488 | -4.564546 | 9.564861 |
| ATvsNS | Follow-up duration | -1.051361 | 13.31846 | -0.08 | 0.937 | -27.15506 | 25.05234 |
|  | Intercept | 5.754241 | 40.42841 | 0.14 | 0.887 | -73.48399 | 84.99247 |
| ATvsRT | Follow-up duration | -1.298396 | 13.28505 | -0.1 | 0.922 | -27.33662 | 24.73983 |
|  | Intercept | 9.5129 | 40.16619 | 0.24 | 0.813 | -69.21139 | 88.23719 |
| ATvsRT-AT | Follow-up duration | 0.627012 | 13.30387 | 0.05 | 0.962 | -25.4481 | 26.70213 |
|  | Intercept | -1.386203 | 40.29182 | -0.03 | 0.973 | -80.35672 | 77.58432 |
| ATvsUC | Follow-up duration | -0.7003838 | 13.28569 | -0.05 | 0.958 | -26.73986 | 25.33909 |
|  | Intercept | 2.878319 | 40.19204 | 0.07 | 0.943 | -75.89663 | 81.65326 |

Supplementary Table24.Meta-regression analysis of the GRIP outcome with mean age as a moderator in older adults with sarcopenic obesity

| **Intervention** | **Covariate** | **Coefficient** | **Standard Error** | **Z-statistic** | **P>z** | **lower confidence interval** | **upper confidence interval** |
| --- | --- | --- | --- | --- | --- | --- | --- |
| ATvsHP | Intercept | 5.290977 | 27.02676 | 0.2 | 0.845 | -47.6805 | 58.26245 |
| ATvsMC | Mean age | -0.1128816 | 5.339352 | -0.02 | 0.983 | -10.57782 | 10.35206 |
|  | Intercept | 11.12895 | 373.9056 | 0.03 | 0.976 | -721.7125 | 743.9704 |
| ATvsMC-NS | Intercept | 3.223426 | 3.420891 | 0.94 | 0.346 | -3.481397 | 9.928249 |
| ATvsNS | Mean age | -0.5502002 | 5.410641 | -0.1 | 0.919 | -11.15486 | 10.05446 |
|  | Intercept | 41.83744 | 379.0765 | 0.11 | 0.912 | -701.1388 | 784.8136 |
| ATvsRT | Mean age | -0.1306871 | 5.336774 | -0.02 | 0.98 | -10.59057 | 10.3292 |
|  | Intercept | 14.25712 | 373.6946 | 0.04 | 0.97 | -718.1707 | 746.685 |
| ATvsRT-AT | Mean age | 0.9167006 | 5.388007 | 0.17 | 0.865 | -9.6436 | 11.477 |
|  | Intercept | -63.6811 | 377.4613 | -0.17 | 0.866 | -803.4917 | 676.1295 |
| ATvsUC | Mean age | -0.3235479 | 5.336844 | -0.06 | 0.952 | -10.78357 | 10.13647 |
|  | Intercept | 24.52189 | 373.7082 | 0.07 | 0.948 | -707.9327 | 756.9765 |

Supplementary Table25.Meta-regression analysis of the FM outcome with country as a moderator in older adults with sarcopenic obesity

| **Intervention** | **Covariate** | **Coefficient** | **Standard Error** | **Z-statistic** | **P>z** | **lower confidence interval** | **upper confidence interval** |
| --- | --- | --- | --- | --- | --- | --- | --- |
| ATvsHP | Intercept | 1.401285 | 28.26115 | 0.05 | 0.96 | -53.98955 | 56.79212 |
| ATvsMC | Intercept | 0.7543781 | 84.5831 | 0.01 | 0.993 | -165.0254 | 166.5342 |
| ATvsMC-NS | Intercept | 0.6543781 | 84.58361 | 0.01 | 0.994 | -165.1265 | 166.4352 |
| ATvsNS | Country | -0.2128821 | 28.19192 | -0.01 | 0.994 | -55.46804 | 55.04228 |
|  | Intercept | 2.346685 | 84.66735 | 0.03 | 0.978 | -163.5983 | 168.2916 |
| ATvsRT | Country | 0.3520031 | 28.2152 | 0.01 | 0.99 | -54.94877 | 55.65278 |
|  | Intercept | -0.7017182 | 84.66475 | -0.01 | 0.993 | -166.6416 | 165.2381 |
| ATvsRT-AT | Intercept | -0.0010006 | 2.118724 | 0 | 1 | -4.153622 | 4.151621 |
| ATvsRT-HP | Country | -0.1044401 | 28.22157 | 0 | 0.997 | -55.41769 | 55.20881 |
|  | Intercept | 0.8804351 | 84.71986 | 0.01 | 0.992 | -165.1674 | 166.9283 |
| ATvsUC | Country | -0.442288 | 28.189 | -0.02 | 0.987 | -55.69172 | 54.80714 |
|  | Intercept | 3.378758 | 84.60725 | 0.04 | 0.968 | -162.4484 | 169.2059 |

Supplementary Table26.Meta-regression analysis of the FM outcome with follow-up duration as a moderator in older adults with sarcopenic obesity

| **Intervention** | **Covariate** | **Coefficient** | **Standard Error** | **Z-statistic** | **P>z** | **lower confidence interval** | **upper confidence interval** |
| --- | --- | --- | --- | --- | --- | --- | --- |
| ATvsHP | Follow-up duration | -1.482172 | 40.12539 | -0.04 | 0.971 | -80.12649 | 77.16215 |
|  | Intercept | 6.711076 | 120.449 | 0.06 | 0.956 | -229.3647 | 242.7869 |
| ATvsMC | Intercept | 2.364532 | 2.29863 | 1.03 | 0.304 | -2.1407 | 6.869764 |
| ATvsMC-NS | Intercept | 2.264532 | 2.317446 | 0.98 | 0.328 | -2.277579 | 6.806644 |
| ATvsNS | Follow-up duration | -0.8928225 | 39.793 | -0.02 | 0.982 | -78.88567 | 77.10002 |
|  | Intercept | 5.243 | 119.4519 | 0.04 | 0.965 | -228.8784 | 239.3644 |
| ATvsRT | Follow-up duration | 0.4935802 | 39.80714 | 0.01 | 0.99 | -77.52698 | 78.51414 |
|  | Intercept | -1.512728 | 119.4789 | -0.01 | 0.99 | -235.6871 | 232.6616 |
| ATvsRT-AT | Intercept | -0.0009833 | 2.118812 | 0 | 1 | -4.153778 | 4.151811 |
| ATvsRT-HP | Intercept | -0.0356816 | 39.85084 | 0 | 0.999 | -78.14188 | 78.07052 |
| ATvsUC | Follow-up duration | -1.082115 | 39.79139 | -0.03 | 0.978 | -79.07181 | 76.90758 |
|  | Intercept | 5.710952 | 119.4313 | 0.05 | 0.962 | -228.37 | 239.7919 |

Supplementary Table27.Meta-regression analysis of the FM outcome with mean age as a moderator in older adults with sarcopenic obesity

| **Intervention** | **Covariate** | **Coefficient** | **Standard Error** | **Z-statistic** | **P>z** | **lower confidence interval** | **upper confidence interval** |
| --- | --- | --- | --- | --- | --- | --- | --- |
| ATvsHP | Mean age | 0.0830367 | 7.682046 | 0.01 | 0.991 | -14.9735 | 15.13957 |
|  | Intercept | -4.041576 | 537.5113 | -0.01 | 0.994 | -1057.544 | 1049.461 |
| ATvsMC | Intercept | 1.788158 | 2.353832 | 0.76 | 0.447 | -2.825268 | 6.401585 |
| ATvsMC-NS | Intercept | 1.688158 | 2.372211 | 0.71 | 0.477 | -2.961289 | 6.337606 |
| ATvsNS | Mean age | 0.1391438 | 7.667442 | 0.02 | 0.986 | -14.88877 | 15.16705 |
|  | Intercept | -7.647701 | 536.6601 | -0.01 | 0.989 | -1059.482 | 1044.187 |
| ATvsRT | Mean age | 0.2984187 | 7.666817 | 0.04 | 0.969 | -14.72827 | 15.3251 |
|  | Intercept | -20.27039 | 536.6122 | -0.04 | 0.97 | -1072.011 | 1031.47 |
| ATvsRT-AT | Intercept | -0.0005212 | 2.11893 | 0 | 1 | -4.153549 | 4.152506 |
| ATvsRT-HP | Mean age | 0.5743367 | 7.705907 | 0.07 | 0.941 | -14.52896 | 15.67764 |
|  | Intercept | -37.52522 | 538.735 | -0.07 | 0.944 | -1093.426 | 1018.376 |
| ATvsUC | Mean age | 0.0430317 | 7.665722 | 0.01 | 0.996 | -14.98151 | 15.06757 |
|  | Intercept | -1.241221 | 536.5386 | 0 | 0.998 | -1052.838 | 1050.355 |

Supplementary Table28.Meta-regression analysis of the PBF outcome with country as a moderator in older adults with sarcopenic obesity

| **Intervention** | **Covariate** | **Coefficient** | **Standard Error** | **Z-statistic** | **P>z** | **lower confidence interval** | **upper confidence interval** |
| --- | --- | --- | --- | --- | --- | --- | --- |
| ATvsHP | Intercept | 0.6115411 | 6.283142 | 0.1 | 0.922 | -11.70319 | 12.92627 |
| ATvsMC | Country | -0.8468018 | 1.808882 | -0.47 | 0.64 | -4.392146 | 2.698543 |
|  | Intercept | 2.656704 | 3.857052 | 0.69 | 0.491 | -4.902979 | 10.21639 |
| ATvsMC-NS | Intercept | -0.1946858 | 7.337211 | -0.03 | 0.979 | -14.57535 | 14.18598 |
| ATvsNS | Country | 0.2989202 | 1.893001 | 0.16 | 0.875 | -3.411294 | 4.009135 |
|  | Intercept | -1.389287 | 5.931938 | -0.23 | 0.815 | -13.01567 | 10.2371 |
| ATvsRT | Country | -0.3909245 | 1.788314 | -0.22 | 0.827 | -3.895956 | 3.114107 |
|  | Intercept | 1.306493 | 3.440432 | 0.38 | 0.704 | -5.43663 | 8.049615 |
| ATvsRT-AT | Country | -0.2276907 | 2.468553 | -0.09 | 0.927 | -5.065966 | 4.610584 |
|  | Intercept | 0.2215946 | 4.524105 | 0.05 | 0.961 | -8.645488 | 9.088677 |
| ATvsRT-HP | Intercept | -0.5753509 | 3.929804 | -0.15 | 0.884 | -8.277625 | 7.126923 |
| ATvsUC | Country | -0.1269058 | 1.787578 | -0.07 | 0.943 | -3.630495 | 3.376683 |
|  | Intercept | 2.319394 | 3.433683 | 0.68 | 0.499 | -4.4105 | 9.049288 |

Supplementary Table29.Meta-regression analysis of the PBF outcome with follow-up duration as a moderator in older adults with sarcopenic obesity

| **Intervention** | **Covariate** | **Coefficient** | **Standard Error** | **Z-statistic** | **P>z** | **lower confidence interval** | **upper confidence interval** |
| --- | --- | --- | --- | --- | --- | --- | --- |
| ATvsHP | Intercept | 0.8864237 | 5.259717 | 0.17 | 0.866 | -9.422432 | 11.19528 |
| ATvsMC | Follow-up duration | -1.32567 | 3.648471 | -0.36 | 0.716 | -8.476542 | 5.825201 |
|  | Intercept | 3.970304 | 12.09335 | 0.33 | 0.743 | -19.73222 | 27.67282 |
| ATvsMC-NS | Intercept | 1.00494 | 3.67514 | 0.27 | 0.785 | -6.198202 | 8.208082 |
| ATvsNS | Follow-up duration | 0.1846222 | 3.780336 | 0.05 | 0.961 | -7.224699 | 7.593944 |
|  | Intercept | 0.7510733 | 12.96698 | 0.06 | 0.954 | -24.66375 | 26.1659 |
| ATvsRT | Follow-up duration | -0.6268912 | 3.595341 | -0.17 | 0.862 | -7.67363 | 6.419848 |
|  | Intercept | 2.4996 | 11.78244 | 0.21 | 0.832 | -20.59357 | 25.59277 |
| ATvsRT-AT | Follow-up duration | -0.3621853 | 3.747429 | -0.1 | 0.923 | -7.707012 | 6.982642 |
|  | Intercept | 1.082533 | 12.49232 | 0.09 | 0.931 | -23.40197 | 25.56704 |
| ATvsRT-HP | Intercept | -1.107824 | 4.992583 | -0.22 | 0.824 | -10.89311 | 8.677458 |
| ATvsUC | Follow-up duration | -0.388649 | 3.594765 | -0.11 | 0.914 | -7.434259 | 6.656961 |
|  | Intercept | 3.641343 | 11.77725 | 0.31 | 0.757 | -19.44165 | 26.72433 |

Supplementary Table30.Meta-regression analysis of the PBF outcome with mean age as a moderator in older adults with sarcopenic obesity

| **Intervention** | **Covariate** | **Coefficient** | **Standard Error** | **Z-statistic** | **P>z** | **lower confidence interval** | **upper confidence interval** |
| --- | --- | --- | --- | --- | --- | --- | --- |
| ATvsHP | Intercept | -6.881323 | 35.17337 | -0.2 | 0.845 | -75.81986 | 62.05722 |
| ATvsMC | Mean age | 0.0193037 | 2.349309 | 0.01 | 0.993 | -4.585256 | 4.623864 |
|  | Intercept | -0.6730765 | 164.3194 | 0 | 0.997 | -322.7332 | 321.387 |
| ATvsMC-NS | Intercept | 1.550951 | 2.975121 | 0.52 | 0.602 | -4.28018 | 7.382081 |
| ATvsNS | Mean age | 1.108354 | 2.511258 | 0.44 | 0.659 | -3.813622 | 6.03033 |
|  | Intercept | -75.73382 | 176.0755 | -0.43 | 0.667 | -420.8355 | 269.3679 |
| ATvsRT | Mean age | 0.0270228 | 2.34501 | 0.01 | 0.991 | -4.569113 | 4.623159 |
|  | Intercept | -1.802988 | 163.9453 | -0.01 | 0.991 | -323.1298 | 319.5238 |
| ATvsRT-AT | Mean age | 0.7022557 | 2.496072 | 0.28 | 0.778 | -4.189955 | 5.594466 |
|  | Intercept | -49.18065 | 174.9979 | -0.28 | 0.779 | -392.1702 | 293.8089 |
| ATvsRT-HP | Intercept | -1.28149 | 23.4691 | -0.05 | 0.956 | -47.28007 | 44.71709 |
|  | Mean age | 0.5727463 | 2.346703 | 0.24 | 0.807 | -4.026706 | 5.172199 |
| ATvsUC | Intercept | -37.18337 | 164.0614 | -0.23 | 0.821 | -358.7378 | 284.3711 |

Supplementary Table31.Meta-regression analysis of the SMI outcome with country as a moderator in older adults with sarcopenic obesity

| **Intervention** | **Covariate** | **Coefficient** | **Standard Error** | **Z-statistic** | **P>z** | **lower confidence interval** | **upper confidence interval** |
| --- | --- | --- | --- | --- | --- | --- | --- |
| MCvsMC-NS | Intercept | 1 | 0.8602325 | 1.16 | 0.245 | -0.6860248 | 2.686025 |
| MCvsNS | Intercept | 0.5 | 0.9219523 | 0.54 | 0.588 | -1.306993 | 2.306993 |
| MCvsRT | Country | -0.2091627 | 50.9803 | 0 | 0.997 | -100.1287 | 99.71039 |
|  | Intercept | 0.4183255 | 101.9596 | 0 | 0.997 | -199.4189 | 200.2555 |
| MCvsUC | Country | 0.6681782 | 25.49551 | 0.03 | 0.979 | -49.30211 | 50.63846 |
|  | Intercept | -0.9045344 | 76.47176 | -0.01 | 0.991 | -150.7864 | 148.9774 |

Supplementary Table32.Meta-regression analysis of the SMI outcome with follow-up duration as a moderator in older adults with sarcopenic obesity

| **Intervention** | **Covariate** | **Coefficient** | **Standard Error** | **Z-statistic** | **P>z** | **lower confidence interval** | **upper confidence interval** |
| --- | --- | --- | --- | --- | --- | --- | --- |
| MCvsMC-NS | Intercept | 0.9573448 | 0.8515289 | 1.12 | 0.261 | -0.7116212 | 2.626311 |
| MCvsNS | Intercept | 0.4573448 | 0.9138367 | 0.5 | 0.617 | -1.333742 | 2.248432 |
| MCvsRT | Follow-up duration | -4.951204 | 5.016417 | -0.99 | 0.324 | -14.7832 | 4.880793 |
|  | Intercept | 16.09758 | 16.2577 | 0.99 | 0.322 | -15.76692 | 47.96207 |
| MCvsUC | Follow-up duration | -5.013621 | 4.945888 | -1.01 | 0.311 | -14.70738 | 4.680142 |
|  | Intercept | 16.0425 | 15.66492 | 1.02 | 0.306 | -14.66019 | 46.74518 |

Supplementary Table33.Meta-regression analysis of the SMI outcome with mean age as a moderator in older adults with sarcopenic obesity

| **Intervention** | **Covariate** | **Coefficient** | **Standard Error** | **Z-statistic** | **P>z** | **lower confidence interval** | **upper confidence interval** |
| --- | --- | --- | --- | --- | --- | --- | --- |
| MCvsMC-NS | Intercept | 0.999704 | 0.8601712 | 1.16 | 0.245 | -0.6862006 | 2.685609 |
| MCvsNS | Intercept | 0.499704 | 0.9218951 | 0.54 | 0.588 | -1.307177 | 2.306585 |
| MCvsRT | Mean age | 0.1517674 | 0.1213933 | 1.25 | 0.211 | -0.0861591 | 0.3896939 |
|  | Intercept | -9.106041 | 7.343629 | -1.24 | 0.215 | -23.49929 | 5.287208 |
| MCvsUC | Mean age | 0.2430859 | 0.2299651 | 1.06 | 0.29 | -0.2076374 | 0.6938092 |
|  | Intercept | -15.9167 | 15.63493 | -1.02 | 0.309 | -46.56059 | 14.7272 |

SMI


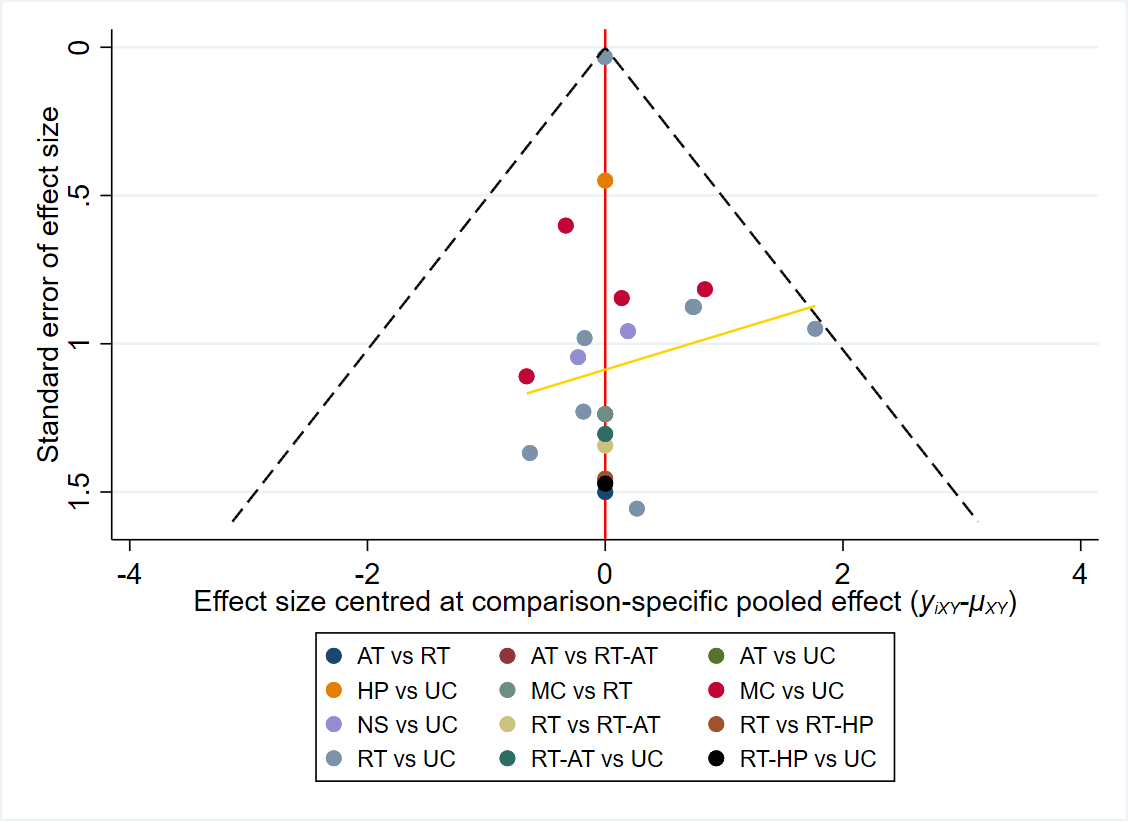


Supplementary Figure6.Comparison-adjusted funnel plot for the BMI outcome in older adults with sarcopenic obesity
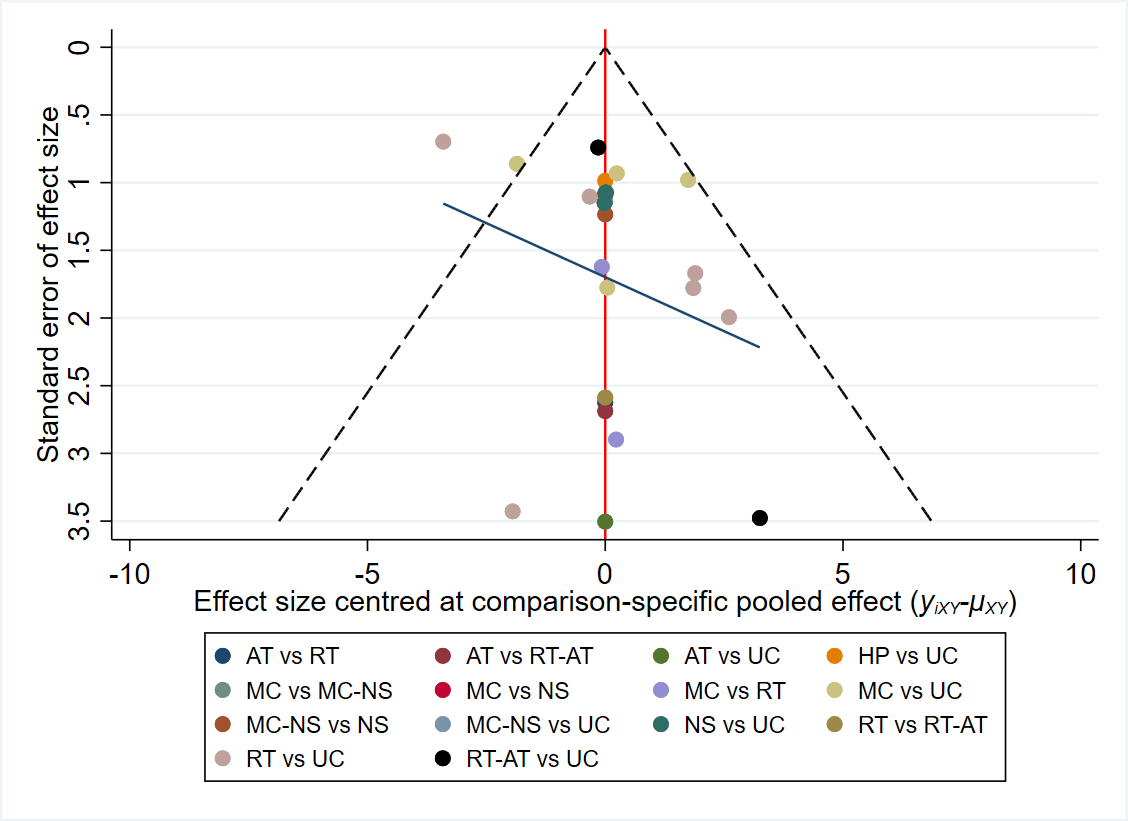
Supplementary Figure7.Comparison-adjusted funnel plot for the GRIP outcome in older adults with sarcopenic obesity


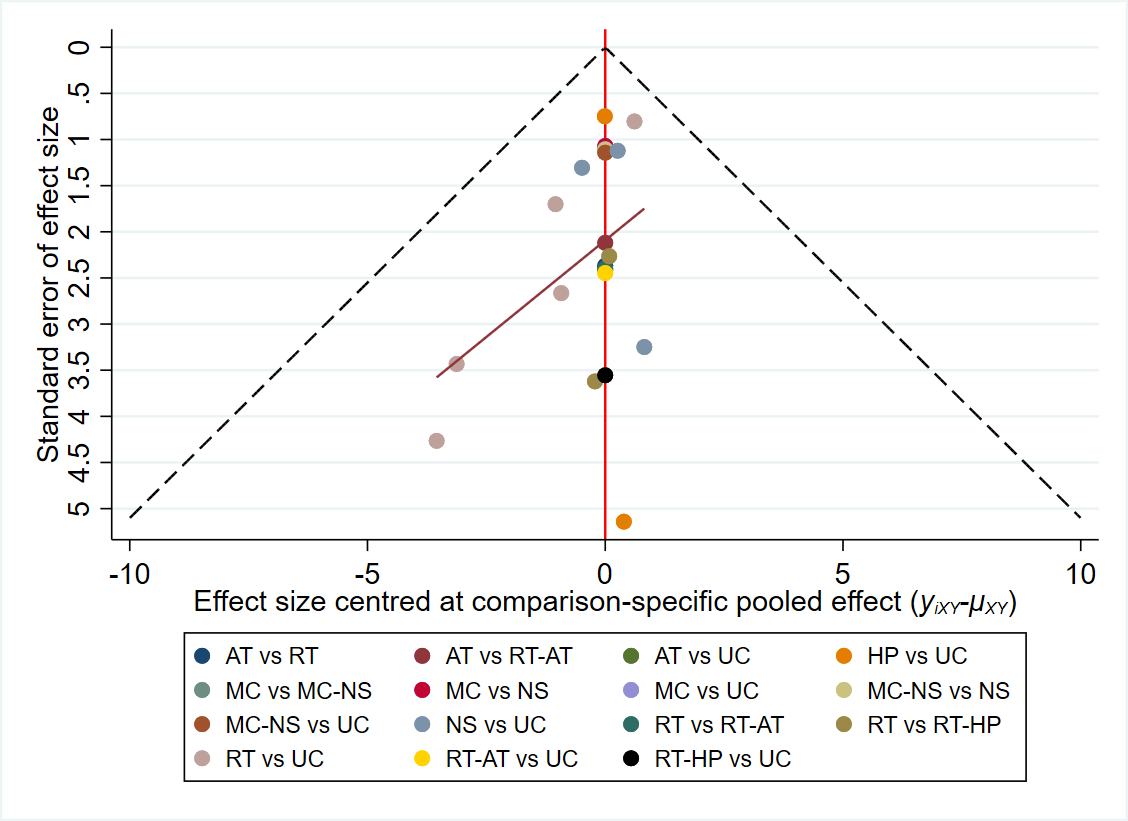


Supplementary Figure8.Comparison-adjusted funnel plot for the FM outcome in older adults with sarcopenic obesity


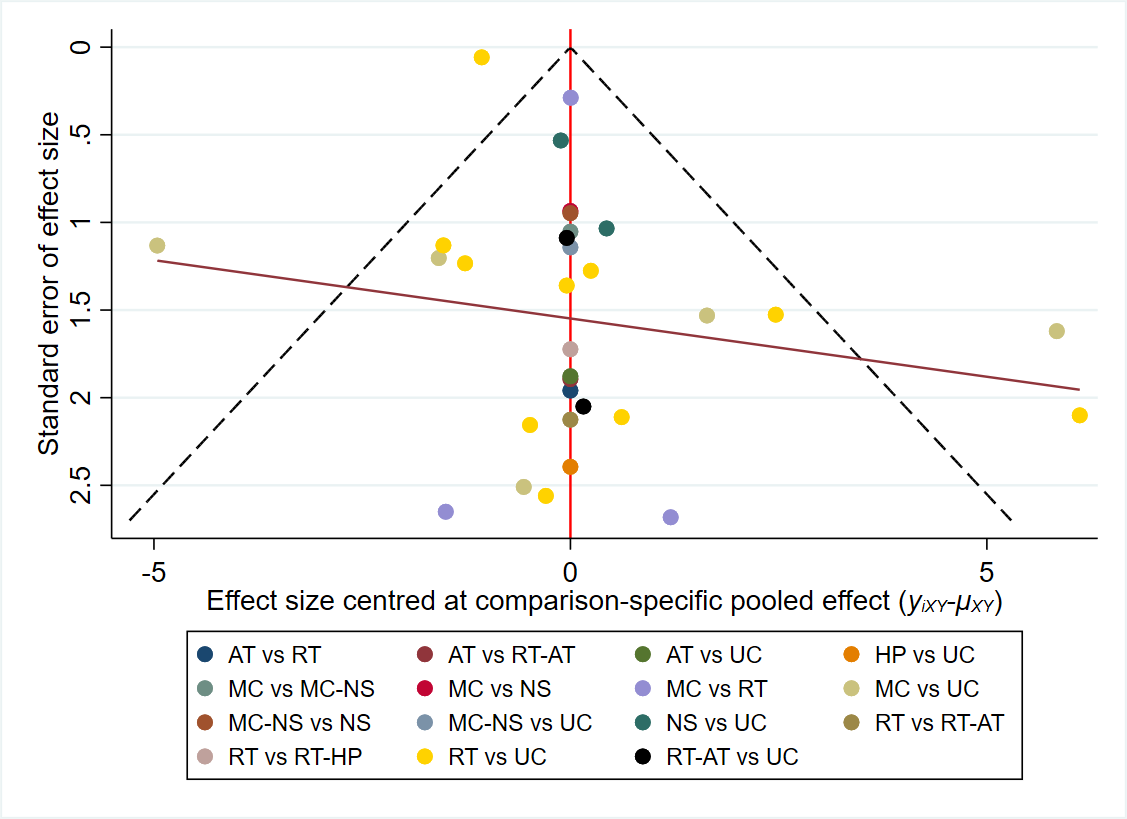


Supplementary Figure9.Comparison-adjusted funnel plot for the PBF outcome in older adults with sarcopenic obesity


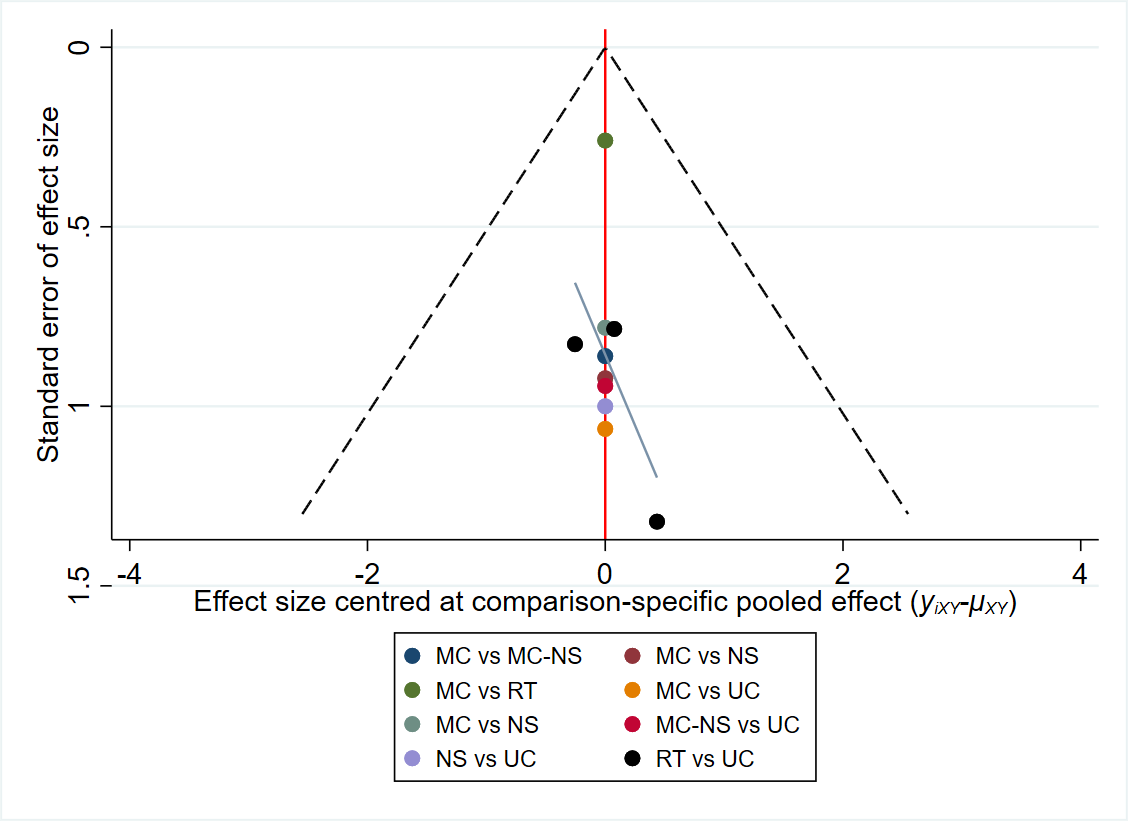


Supplementary Figure10.Comparison-adjusted funnel plot for the SMI outcome in older adults with sarcopenic obesity

Supplementary Table34.GRADE certainty of evidence for the BMI outcome in older adults with sarcopenic obesity

| **Comparison** | **Number of studies** | **Within-study bias** | **Reporting bias** | **Indirectness** | **Imprecision** | **Heterogeneity** | **Incoherence** | **Confidence rating** | **Reason**  **(s) for downgrading** |
| --- | --- | --- | --- | --- | --- | --- | --- | --- | --- |
| AT:RT | 1 | Some concerns | Low risk | No concerns | Some concerns | No concerns | No concerns | Low | ["Within-study bias","Imprecision"] |
| AT:RT-AT | 1 | Some concerns | Low risk | No concerns | Some concerns | No concerns | No concerns | Low | ["Within-study bias","Imprecision"] |
| AT:UC | 1 | Some concerns | Low risk | No concerns | Some concerns | No concerns | No concerns | Low | ["Within-study bias","Imprecision"] |
| HP:UC | 1 | No concerns | Low risk | No concerns | Some concerns | No concerns | No concerns | Moderate | ["Imprecision"] |
| MC:RT | 1 | Some concerns | Low risk | No concerns | No concerns | No concerns | No concerns | Moderate | ["Within-study bias"] |
| MC:UC | 4 | Some concerns | Low risk | No concerns | No concerns | No concerns | No concerns | Moderate | ["Within-study bias"] |
| NS:UC | 2 | No concerns | Low risk | No concerns | No concerns | No concerns | No concerns | High | [] |
| RT:RT-AT | 1 | Some concerns | Low risk | No concerns | Some concerns | No concerns | No concerns | Low | ["Within-study bias","Imprecision"] |
| RT:RT-HP | 1 | No concerns | Low risk | No concerns | Some concerns | No concerns | No concerns | Moderate | ["Imprecision"] |
| RT:UC | 8 | No concerns | Low risk | No concerns | No concerns | No concerns | No concerns | High | [] |
| RT-AT:UC | 1 | Some concerns | Low risk | No concerns | Some concerns | No concerns | No concerns | Low | ["Within-study bias","Imprecision"] |
| RT-HP:UC | 1 | No concerns | Low risk | No concerns | Some concerns | No concerns | No concerns | Moderate | ["Imprecision"] |
| AT:HP | 0 | Some concerns | Low risk | No concerns | Some concerns | No concerns | No concerns | Low | ["Within-study bias","Imprecision"] |
| AT:MC | 0 | Some concerns | Low risk | No concerns | Some concerns | No concerns | No concerns | Low | ["Within-study bias","Imprecision"] |
| AT:NS | 0 | Some concerns | Low risk | No concerns | Some concerns | Some concerns | No concerns | Very low | ["Within-study bias","Imprecision","Heterogeneity"] |
| AT:RT-HP | 0 | Some concerns | Low risk | No concerns | Some concerns | No concerns | No concerns | Low | ["Within-study bias","Imprecision"] |
| HP:MC | 0 | No concerns | Low risk | No concerns | No concerns | No concerns | No concerns | High | [] |
| HP:NS | 0 | No concerns | Low risk | No concerns | Some concerns | No concerns | No concerns | Moderate | ["Imprecision"] |
| HP:RT | 0 | No concerns | Low risk | No concerns | No concerns | No concerns | No concerns | High | [] |
| HP:RT-AT | 0 | Some concerns | Low risk | No concerns | Some concerns | No concerns | No concerns | Low | ["Within-study bias","Imprecision"] |
| HP:RT-HP | 0 | No concerns | Low risk | No concerns | Some concerns | Some concerns | No concerns | Low | ["Imprecision","Heterogeneity"] |
| MC:NS | 0 | No concerns | Low risk | No concerns | No concerns | Some concerns | No concerns | Moderate | ["Heterogeneity"] |
| MC:RT-AT | 0 | Some concerns | Low risk | No concerns | Some concerns | No concerns | No concerns | Low | ["Within-study bias","Imprecision"] |
| MC:RT-HP | 0 | No concerns | Low risk | No concerns | Some concerns | No concerns | No concerns | Moderate | ["Imprecision"] |
| NS:RT | 0 | No concerns | Low risk | No concerns | No concerns | Some concerns | No concerns | Moderate | ["Heterogeneity"] |
| NS:RT-AT | 0 | Some concerns | Low risk | No concerns | Some concerns | No concerns | No concerns | Low | ["Within-study bias","Imprecision"] |
| NS:RT-HP | 0 | No concerns | Low risk | No concerns | Some concerns | No concerns | No concerns | Moderate | ["Imprecision"] |
| RT-AT:RT-HP | 0 | Some concerns | Low risk | No concerns | Some concerns | No concerns | No concerns | Low | ["Within-study bias","Imprecision"] |

Supplementary Table35.GRADE certainty of evidence for the GRIP outcome in older adults with sarcopenic obesity

| **Comparison** | **Number of studies** | **Within-study bias** | **Reporting bias** | **Indirectness** | **Imprecision** | **Heterogeneity** | **Incoherence** | **Confidence rating** | **Reason**  **(s) for downgrading** |
| --- | --- | --- | --- | --- | --- | --- | --- | --- | --- |
| AT:RT | 1 | Some concerns | Low risk | No concerns | Some concerns | No concerns | No concerns | Low | ["Within-study bias","Imprecision"] |
| AT:RT-AT | 1 | Some concerns | Low risk | No concerns | Some concerns | No concerns | No concerns | Low | ["Within-study bias","Imprecision"] |
| AT:UC | 1 | Some concerns | Low risk | No concerns | Some concerns | No concerns | No concerns | Low | ["Within-study bias","Imprecision"] |
| HP:UC | 1 | No concerns | Low risk | No concerns | Some concerns | No concerns | No concerns | Moderate | ["Imprecision"] |
| MC:MC-NS | 1 | No concerns | Low risk | No concerns | Some concerns | No concerns | No concerns | Moderate | ["Imprecision"] |
| MC:NS | 1 | No concerns | Low risk | No concerns | Some concerns | No concerns | No concerns | Moderate | ["Imprecision"] |
| MC:RT | 2 | No concerns | Low risk | No concerns | Some concerns | Some concerns | No concerns | Low | ["Imprecision","Heterogeneity"] |
| MC:UC | 4 | No concerns | Low risk | No concerns | No concerns | Some concerns | No concerns | Moderate | ["Heterogeneity"] |
| MC-NS:NS | 1 | No concerns | Low risk | No concerns | Some concerns | No concerns | No concerns | Moderate | ["Imprecision"] |
| MC-NS:UC | 1 | No concerns | Low risk | No concerns | Some concerns | No concerns | No concerns | Moderate | ["Imprecision"] |
| NS:UC | 2 | No concerns | Low risk | No concerns | Some concerns | No concerns | No concerns | Moderate | ["Imprecision"] |
| RT:RT-AT | 1 | Some concerns | Low risk | No concerns | Some concerns | No concerns | No concerns | Low | ["Within-study bias","Imprecision"] |
| RT:UC | 6 | No concerns | Low risk | No concerns | No concerns | Some concerns | No concerns | Moderate | ["Heterogeneity"] |
| RT-AT:UC | 2 | Some concerns | Low risk | No concerns | Some concerns | Some concerns | No concerns | Very low | ["Within-study bias","Imprecision","Heterogeneity"] |
| AT:HP | 0 | No concerns | Low risk | No concerns | Some concerns | No concerns | No concerns | Moderate | ["Imprecision"] |
| AT:MC | 0 | Some concerns | Low risk | No concerns | Some concerns | No concerns | No concerns | Low | ["Within-study bias","Imprecision"] |
| AT:MC-NS | 0 | No concerns | Low risk | No concerns | Some concerns | No concerns | No concerns | Moderate | ["Imprecision"] |
| AT:NS | 0 | No concerns | Low risk | No concerns | Some concerns | No concerns | No concerns | Moderate | ["Imprecision"] |
| HP:MC | 0 | No concerns | Low risk | No concerns | Some concerns | No concerns | No concerns | Moderate | ["Imprecision"] |
| HP:MC-NS | 0 | No concerns | Low risk | No concerns | Some concerns | No concerns | No concerns | Moderate | ["Imprecision"] |
| HP:NS | 0 | No concerns | Low risk | No concerns | Some concerns | No concerns | No concerns | Moderate | ["Imprecision"] |
| HP:RT | 0 | No concerns | Low risk | No concerns | Some concerns | No concerns | No concerns | Moderate | ["Imprecision"] |
| HP:RT-AT | 0 | No concerns | Low risk | No concerns | Some concerns | No concerns | No concerns | Moderate | ["Imprecision"] |
| MC:RT-AT | 0 | Some concerns | Low risk | No concerns | Some concerns | No concerns | No concerns | Low | ["Within-study bias","Imprecision"] |
| MC-NS:RT | 0 | No concerns | Low risk | No concerns | Some concerns | No concerns | No concerns | Moderate | ["Imprecision"] |
| MC-NS:RT-AT | 0 | No concerns | Low risk | No concerns | Some concerns | No concerns | No concerns | Moderate | ["Imprecision"] |
| NS:RT | 0 | No concerns | Low risk | No concerns | Some concerns | Some concerns | No concerns | Low | ["Imprecision","Heterogeneity"] |
| NS:RT-AT | 0 | No concerns | Low risk | No concerns | Some concerns | No concerns | No concerns | Moderate | ["Imprecision"] |

Supplementary Table36.GRADE certainty of evidence for the FM outcome in older adults with sarcopenic obesity

| **Comparison** | **Number of studies** | **Within-study bias** | **Reporting bias** | **Indirectness** | **Imprecision** | **Heterogeneity** | **Incoherence** | **Confidence rating** | **Reason**  **(s) for downgrading** |
| --- | --- | --- | --- | --- | --- | --- | --- | --- | --- |
| AT:RT | 1 | Some concerns | Low risk | No concerns | Some concerns | No concerns | No concerns | Low | ["Within-study bias","Imprecision"] |
| AT:RT-AT | 1 | Some concerns | Low risk | No concerns | Some concerns | No concerns | No concerns | Low | ["Within-study bias","Imprecision"] |
| AT:UC | 1 | Some concerns | Low risk | No concerns | Some concerns | No concerns | No concerns | Low | ["Within-study bias","Imprecision"] |
| HP:UC | 2 | No concerns | Low risk | No concerns | No concerns | No concerns | No concerns | High | [] |
| MC:MC-NS | 1 | No concerns | Low risk | No concerns | No concerns | Some concerns | No concerns | Moderate | ["Heterogeneity"] |
| MC:NS | 1 | No concerns | Low risk | No concerns | No concerns | Some concerns | No concerns | Moderate | ["Heterogeneity"] |
| MC:UC | 1 | No concerns | Low risk | No concerns | No concerns | Some concerns | No concerns | Moderate | ["Heterogeneity"] |
| MC-NS:NS | 1 | No concerns | Low risk | No concerns | Some concerns | No concerns | No concerns | Moderate | ["Imprecision"] |
| MC-NS:UC | 1 | No concerns | Low risk | No concerns | No concerns | Some concerns | No concerns | Moderate | ["Heterogeneity"] |
| NS:UC | 3 | No concerns | Low risk | No concerns | No concerns | No concerns | No concerns | High | [] |
| RT:RT-AT | 1 | Some concerns | Low risk | No concerns | Some concerns | No concerns | No concerns | Low | ["Within-study bias","Imprecision"] |
| RT:RT-HP | 2 | No concerns | Low risk | No concerns | Some concerns | No concerns | No concerns | Moderate | ["Imprecision"] |
| RT:UC | 5 | Some concerns | Low risk | No concerns | No concerns | No concerns | No concerns | Moderate | ["Within-study bias"] |
| RT-AT:UC | 1 | Some concerns | Low risk | No concerns | Some concerns | No concerns | No concerns | Low | ["Within-study bias","Imprecision"] |
| RT-HP:UC | 1 | No concerns | Low risk | No concerns | Some concerns | No concerns | No concerns | Moderate | ["Imprecision"] |
| AT:HP | 0 | Some concerns | Low risk | No concerns | Some concerns | Some concerns | No concerns | Very low | ["Within-study bias","Imprecision","Heterogeneity"] |
| AT:MC | 0 | No concerns | Low risk | No concerns | Some concerns | Some concerns | No concerns | Low | ["Imprecision","Heterogeneity"] |
| AT:MC-NS | 0 | No concerns | Low risk | No concerns | Some concerns | Some concerns | No concerns | Low | ["Imprecision","Heterogeneity"] |
| AT:NS | 0 | Some concerns | Low risk | No concerns | Some concerns | No concerns | No concerns | Low | ["Within-study bias","Imprecision"] |
| AT:RT-HP | 0 | Some concerns | Low risk | No concerns | Some concerns | No concerns | No concerns | Low | ["Within-study bias","Imprecision"] |
| HP:MC | 0 | No concerns | Low risk | No concerns | Some concerns | No concerns | No concerns | Moderate | ["Imprecision"] |
| HP:MC-NS | 0 | No concerns | Low risk | No concerns | Some concerns | No concerns | No concerns | Moderate | ["Imprecision"] |
| HP:NS | 0 | No concerns | Low risk | No concerns | Some concerns | No concerns | No concerns | Moderate | ["Imprecision"] |
| HP:RT | 0 | No concerns | Low risk | No concerns | Some concerns | No concerns | No concerns | Moderate | ["Imprecision"] |
| HP:RT-AT | 0 | Some concerns | Low risk | No concerns | Some concerns | Some concerns | No concerns | Very low | ["Within-study bias","Imprecision","Heterogeneity"] |
| HP:RT-HP | 0 | No concerns | Low risk | No concerns | Some concerns | Some concerns | No concerns | Low | ["Imprecision","Heterogeneity"] |
| MC:RT | 0 | No concerns | Low risk | No concerns | Some concerns | No concerns | No concerns | Moderate | ["Imprecision"] |
| MC:RT-AT | 0 | No concerns | Low risk | No concerns | Some concerns | Some concerns | No concerns | Low | ["Imprecision","Heterogeneity"] |
| MC:RT-HP | 0 | No concerns | Low risk | No concerns | Some concerns | Some concerns | No concerns | Low | ["Imprecision","Heterogeneity"] |
| MC-NS:RT | 0 | No concerns | Low risk | No concerns | Some concerns | No concerns | No concerns | Moderate | ["Imprecision"] |
| MC-NS:RT-AT | 0 | No concerns | Low risk | No concerns | Some concerns | Some concerns | No concerns | Low | ["Imprecision","Heterogeneity"] |
| MC-NS:RT-HP | 0 | No concerns | Low risk | No concerns | Some concerns | Some concerns | No concerns | Low | ["Imprecision","Heterogeneity"] |
| NS:RT | 0 | No concerns | Low risk | No concerns | No concerns | Some concerns | No concerns | Moderate | ["Heterogeneity"] |
| NS:RT-AT | 0 | Some concerns | Low risk | No concerns | Some concerns | No concerns | No concerns | Low | ["Within-study bias","Imprecision"] |
| NS:RT-HP | 0 | No concerns | Low risk | No concerns | Some concerns | No concerns | No concerns | Moderate | ["Imprecision"] |
| RT-AT:RT-HP | 0 | Some concerns | Low risk | No concerns | Some concerns | No concerns | No concerns | Low | ["Within-study bias","Imprecision"] |

Supplementary Table37.GRADE certainty of evidence for the PBF outcome in older adults with sarcopenic obesity

| **Comparison** | **Number of studies** | **Within-study bias** | **Reporting bias** | **Indirectness** | **Imprecision** | **Heterogeneity** | **Incoherence** | **Confidence rating** | **Reason**  **(s) for downgrading** |
| --- | --- | --- | --- | --- | --- | --- | --- | --- | --- |
| AT:RT | 1 | Some concerns | Low risk | No concerns | Some concerns | No concerns | No concerns | Low | ["Within-study bias","Imprecision"] |
| AT:RT-AT | 1 | Some concerns | Low risk | No concerns | Some concerns | No concerns | No concerns | Low | ["Within-study bias","Imprecision"] |
| AT:UC | 1 | Some concerns | Low risk | No concerns | Some concerns | No concerns | No concerns | Low | ["Within-study bias","Imprecision"] |
| HP:UC | 1 | Some concerns | Low risk | No concerns | Some concerns | No concerns | Some concerns | Very low | ["Within-study bias","Imprecision","Incoherence"] |
| MC:MC-NS | 1 | No concerns | Low risk | No concerns | Some concerns | No concerns | No concerns | Moderate | ["Imprecision"] |
| MC:NS | 1 | No concerns | Low risk | No concerns | Some concerns | Some concerns | No concerns | Moderate | ["Imprecision","Heterogeneity"] |
| MC:RT | 3 | No concerns | Low risk | No concerns | Some concerns | No concerns | Major concerns | Very low | ["Imprecision","Incoherence"] |
| MC:UC | 5 | No concerns | Low risk | No concerns | No concerns | Some concerns | Some concerns | Low | ["Heterogeneity","Incoherence"] |
| MC-NS:NS | 1 | No concerns | Low risk | No concerns | Some concerns | No concerns | No concerns | Moderate | ["Imprecision"] |
| MC-NS:UC | 1 | No concerns | Low risk | No concerns | Some concerns | No concerns | No concerns | Moderate | ["Imprecision"] |
| NS:UC | 2 | No concerns | Low risk | No concerns | Some concerns | No concerns | No concerns | Moderate | ["Imprecision"] |
| RT:RT-AT | 1 | Some concerns | Low risk | No concerns | Some concerns | No concerns | No concerns | Low | ["Within-study bias","Imprecision"] |
| RT:RT-HP | 1 | No concerns | Low risk | No concerns | Some concerns | No concerns | Some concerns | Low | ["Imprecision","Incoherence"] |
| RT:UC | 10 | No concerns | Low risk | No concerns | No concerns | Some concerns | Some concerns | Low | ["Heterogeneity","Incoherence"] |
| RT-AT:UC | 2 | Some concerns | Low risk | No concerns | Some concerns | Some concerns | No concerns | Very low | ["Within-study bias","Imprecision","Heterogeneity"] |
| AT:HP | 0 | Some concerns | Low risk | No concerns | Some concerns | No concerns | Some concerns | Very low | ["Within-study bias","Imprecision","Incoherence"] |
| AT:MC | 0 | Some concerns | Low risk | No concerns | Some concerns | No concerns | Some concerns | Very low | ["Within-study bias","Imprecision","Incoherence"] |
| AT:MC-NS | 0 | No concerns | Low risk | No concerns | Some concerns | No concerns | Some concerns | Low | ["Imprecision","Incoherence"] |
| AT:NS | 0 | No concerns | Low risk | No concerns | Some concerns | No concerns | Some concerns | Low | ["Imprecision","Incoherence"] |
| AT:RT-HP | 0 | No concerns | Low risk | No concerns | Some concerns | No concerns | Some concerns | Low | ["Imprecision","Incoherence"] |
| HP:MC | 0 | Some concerns | Low risk | No concerns | Some concerns | No concerns | Some concerns | Very low | ["Within-study bias","Imprecision","Incoherence"] |
| HP:MC-NS | 0 | No concerns | Low risk | No concerns | Some concerns | No concerns | Some concerns | Low | ["Imprecision","Incoherence"] |
| HP:NS | 0 | No concerns | Low risk | No concerns | Some concerns | No concerns | Some concerns | Low | ["Imprecision","Incoherence"] |
| HP:RT | 0 | Some concerns | Low risk | No concerns | Some concerns | No concerns | Some concerns | Very low | ["Within-study bias","Imprecision","Incoherence"] |
| HP:RT-AT | 0 | Some concerns | Low risk | No concerns | Some concerns | No concerns | Some concerns | Very low | ["Within-study bias","Imprecision","Incoherence"] |
| HP:RT-HP | 0 | No concerns | Low risk | No concerns | Some concerns | No concerns | Some concerns | Low | ["Imprecision","Incoherence"] |
| MC:RT-AT | 0 | Some concerns | Low risk | No concerns | Some concerns | No concerns | Some concerns | Very low | ["Within-study bias","Imprecision","Incoherence"] |
| MC:RT-HP | 0 | No concerns | Low risk | No concerns | Some concerns | No concerns | Some concerns | Low | ["Imprecision","Incoherence"] |
| MC-NS:RT | 0 | No concerns | Low risk | No concerns | Some concerns | No concerns | Some concerns | Low | ["Imprecision","Incoherence"] |
| MC-NS:RT-AT | 0 | No concerns | Low risk | No concerns | Some concerns | No concerns | Some concerns | Low | ["Imprecision","Incoherence"] |
| MC-NS:RT-HP | 0 | No concerns | Low risk | No concerns | Some concerns | No concerns | Some concerns | Low | ["Imprecision","Incoherence"] |
| NS:RT | 0 | No concerns | Low risk | No concerns | Some concerns | Some concerns | Some concerns | Very low | ["Imprecision","Heterogeneity","Incoherence"] |
| NS:RT-AT | 0 | No concerns | Low risk | No concerns | Some concerns | No concerns | Some concerns | Low | ["Imprecision","Incoherence"] |
| NS:RT-HP | 0 | No concerns | Low risk | No concerns | Some concerns | No concerns | Some concerns | Low | ["Imprecision","Incoherence"] |
| RT-AT:RT-HP | 0 | No concerns | Low risk | No concerns | Some concerns | No concerns | Some concerns | Low | ["Imprecision","Incoherence"] |
| RT-HP:UC | 0 | No concerns | Low risk | No concerns | Some concerns | No concerns | Some concerns | Low | ["Imprecision","Incoherence"] |

Supplementary Table38.GRADE certainty of evidence for the SMI outcome in older adults with sarcopenic obesity

| **Comparison** | **Number of studies** | **Within-study bias** | **Reporting bias** | **Indirectness** | **Imprecision** | **Heterogeneity** | **Incoherence** | **Confidence rating** | **Reason**  **(s) for downgrading** |
| --- | --- | --- | --- | --- | --- | --- | --- | --- | --- |
| MC:MC-NS | 1 | No concerns | Low risk | No concerns | Some concerns | Some concerns | No concerns | Low | ["Imprecision","Heterogeneity"] |
| MC:NS | 1 | No concerns | Low risk | No concerns | Some concerns | Some concerns | No concerns | Low | ["Imprecision","Heterogeneity"] |
| MC:RT | 1 | No concerns | Low risk | No concerns | Some concerns | Some concerns | No concerns | Low | ["Imprecision","Heterogeneity"] |
| MC:UC | 1 | No concerns | Low risk | No concerns | Some concerns | Some concerns | No concerns | Low | ["Imprecision","Heterogeneity"] |
| MC-NS:NS | 1 | No concerns | Low risk | No concerns | Some concerns | Some concerns | No concerns | Low | ["Imprecision","Heterogeneity"] |
| MC-NS:UC | 1 | No concerns | Low risk | No concerns | No concerns | Some concerns | No concerns | Moderate | ["Heterogeneity"] |
| NS:UC | 1 | No concerns | Low risk | No concerns | Some concerns | Some concerns | No concerns | Low | ["Imprecision","Heterogeneity"] |
| RT:UC | 3 | No concerns | Low risk | No concerns | No concerns | Some concerns | No concerns | Moderate | ["Heterogeneity"] |
| MC-NS:RT | 0 | No concerns | Low risk | No concerns | Some concerns | Some concerns | No concerns | Low | ["Imprecision","Heterogeneity"] |
| NS:RT | 0 | No concerns | Low risk | No concerns | Some concerns | Some concerns | No concerns | Low | ["Imprecision","Heterogeneity"] |

Supplementary Table39.Exercise intensity and baseline BMI varied across the included studies in older adults with sarcopenic obesity

| **First Author** | **Year** | **Exercise intensity** | **Experimental group BMI** | **Control group BMI** |
| --- | --- | --- | --- | --- |
| M Aubertin-Leheudre | 2007 | NA | 29.7 ± 1.0 | 30.7 ± 2.0 |
| Ebrahim Banitalebi | 2021 | Moderate-intensity RT | 33.72 ± 3.15 | 32.53 ± 2.01 |
| Hung-Ting Chen | 2017 | RT：Moderate-intensity ；AT：Moderate-intensity ；AT-RT：Moderate-intensity | RT:28.3 ± 4.4; AT:26.8 ± 3.8; RT-AT:27.2 ± 2.9 | 29.0 ± 3.9 |
| shih-Wei huanG | 2017 | Moderate-intensity RT | 27.31 ± 3.74 | 28.96 ± 3.49 |
| Chun-De Liao | 2018 | Moderate-intensity RT | 27.27 ± 3.72 | 29.16 ± 3.62 |
| Mathieu L | 2016 | High-intensity RT | 27.27 ± 3.72 | 29.16 ± 3.62 |
| espedita Muscariello | 2016 | NA | 27.0 ± 1.4 | 27.6 ± 2.0 |
| Hellen C.G. Nabuco | 2019 | Moderate-intensity RT | 32.0 ± 2.3 | 31.1 ± 2.9 |
| Jinkee Park | 2017 | Moderate-intensity RT | 26.4 ± 3.0 | 27.4 ± 3.0 |
| Rosa Sammarco | 2017 | NA | 32.0 ± 2.3 | 31.1 ± 2.9 |
| Anoop Balachandran | 2014 | Moderate-intensity RT | 32.9 ± 6.8 | 32.4 ± 2.0 |
| Shu-Ching Chiu | 2018 | Moderate-intensity RT | 25.15 ± 3.75 | 24.85 ± 3.01 |
| Hunkyung Kim PhD | 2016 | Moderate-intensityMC | MC-HP:24.9 ± 3.0; MC:25.1 ± 2.5; HP:24.9 ± 2.5 | 25.3 ± 2.8 |
| Chun-De Liao | 2017 | Moderate-intensity RT | 27.32 ± 3.33 | 28.19 ± 3.27 |
| Ebrahim Banitalebi | 2020 | Moderate-intensity RT | 33.72 ± 3.15 | 32.53 ± 2.01 |
| Paolo M. Cunha | 2017 | Moderate-intensity RT | 27.1 ± 4.3 | 26.7 ± 4.6 |
| Andr ́e Bonadias | 2016 | Moderate-intensity RT | 27.10 ± 3.99 | 29.09 ± 5.08 |
| Yu-Hao Lee | 2021 | Moderate-intensity RT | 26.95 ± 3.31 | 28.93 ± 3.55 |
| Elmoetez Magtouf | 2023 | Moderate-to-vigorous intensity MC | 34.5 ± 4.0 | 34.7 ± 2.3 |
| Hamza Ferhi | 2023 | Moderate-to-vigorous intensity | 35.8 ± 2.1 | 35.8 ± 2.7 |
| Won-Sang Jung | 2022 | Vigorous intensity | 22.50 ± 1.75 | 22.58 ± 1.69 |
| Cynthia El Hajj | 2019 | NA | 30.18 ± 5.72 | 28.63 ± 5.22 |
| Luis Polo-Ferrero | 2025 | Moderate-intensity RT | RT:33.6 ±5.2; MC:32.5±3.1 | 32.6±4.8 |
| Xian Guo | 2025 | Moderate-intensity RT | 26.16 ± 2.48 | 25.71 ± 2.31 |
